# Assembly of Bleomycin Saccharide-Decorated Spherical Nucleic Acids

**DOI:** 10.1021/acs.bioconjchem.1c00539

**Published:** 2022-01-05

**Authors:** Ville Tähtinen, Vijay Gulumkar, Sajal K. Maity, Ann-Mari Yliperttula, Saara Siekkinen, Toni Laine, Ekaterina Lisitsyna, Iida Haapalehto, Tapani Viitala, Elina Vuorimaa-Laukkanen, Marjo Yliperttula, Pasi Virta

**Affiliations:** † Department of Chemistry, University of Turku, FI-20500 Turku, Finland; ‡ Division of Pharmaceutical Biosciences, Faculty of Pharmacy, University of Helsinki, FI-00014 Helsinki, Finland; § Faculty of Engineering and Natural Sciences, Tampere University, FI-33014 Tampere, Finland; ∥ Pharmaceutical Sciences, Faculty of Science and Engineering, Åbo Akademi University, 20520 Turku, Finland

## Abstract

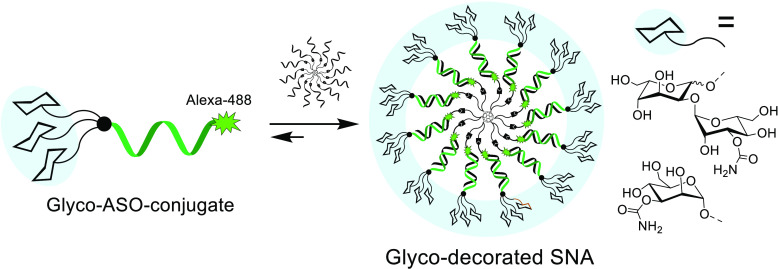

Glyco-decorated spherical nucleic acids (SNAs) may be attractive delivery vehicles, emphasizing the sugar-specific effect on the outer sphere of the construct and at the same time hiding unfavorable distribution properties of the loaded oligonucleotides. As examples of such nanoparticles, tripodal sugar constituents of bleomycin were synthesized and conjugated with a fluorescence-labeled antisense oligonucleotide (AON_ARV7_). Successive copper(I)-catalyzed azide-alkyne and strain-promoted alkyne-nitrone cycloadditions (SPANC) were utilized for the synthesis. Then, the glyco-AON_ARV7_ conjugates were hybridized with complementary strands of a C_60_-based molecular spherical nucleic acid (i.e., a hybridization-mediated carrier). The formation and stability of these assembled glyco-decorated SNAs were evaluated by polyacrylamide gel electrophoresis (PAGE), UV melting profile analysis, and time-resolved fluorescence spectroscopy. Association constants were extracted from time-resolved fluorescence data. Preliminary cellular uptake experiments of the glyco-AON_ARV7_ conjugates (120 nM solutions) and of the corresponding glyco-decorated SNAs (10 nM solutions) with human prostate cancer cells (PC3) showed an efficient uptake in each case. A marked variation in intracellular distribution was observed.

## Introduction

Spherical nucleic acids (SNAs) are nanostructures consisting of an appropriate core (gold, silica, liposomes, proteins) and a densely packed layer of oligonucleotides.^1−9^ Compared to the poor cellular delivery of linear oligonucleotides,^10−13^ SNAs are efficiently taken up by cells via class A scavenger receptor-mediated endocytosis.^14,15^ Furthermore, SNAs are more resistant toward nuclease degradation^16^ and they elicit low innate immune response.^17^ In addition to these beneficial properties of naked SNAs, the radial formulation may be utilized together with the covalent conjugation strategy.^18^ The effect of potential cell/tissue-specific ligands may be emphasized on the outer sphere, which at the same time hide unfavorable distribution properties of the loaded oligonucleotide content. This approach may be particularly useful for weakly interacting ligands, the sufficient cell-targeted delivery potential of which requires multivalent binding to the cell membrane receptors. Carbohydrate–lectin binding is a typical example of such multivalent interaction.^19^

The present article describes the assembly of glyco-decorated and hybridization-mediated SNAs, which consisted of bleomycin (BLM) saccharides as a decoration sphere and a known splice-switching oligonucleotide (AON_ARV7_) payload that is known to suppress prostate tumor cell survival by inhibiting the mRNA synthesis of AR-V7 (an androgen receptor variant).^20^ These nanoparticles may be classified as hybrid structures between fullerene C_60_-based molecular SNAs and “glyco-superballs” that have gained marked interest as virus-like synthetic macromolecules.^21−24^ Bleomycin saccharides (i.e., 2-*O*-(3-carbamoyl-α-d-mannopyranosyl)-l-gulose and 3-carbamoyl-α-d-mannose) are constituents of bleomycin (BLM), which is a glycopeptoid-derived natural compound used in clinics as an antitumor agent against squamous cell carcinomas and malignant lymphomas.^25−30^ The peptoid moiety of bleomycin is responsible for the cytotoxicity (oxidative cleavage of DNA and RNA),^31−37^ whereas the tumor cell selectivity derives from the disaccharide moiety.^38−41^ The reason for the selective uptake by tumor cells is still not fully understood, but data suggest that bleomycins are transported into cells by cell surface receptors involved in glucose transport, which is upregulated in tumor cells.^41^ The position and substitution of the carbamoyl group in the disaccharide are important for the uptake by cancer cells, and the cell uptake could be even improved by varying the site of the carbamoyl group.^42^ The carbamoyl mannose moiety alone also mediates selective tumor cell targeting.^40^ Furthermore, clusters consisting of bleomycin disaccharide or carbamoyl mannose units are taken up by cells more efficiently, indicating that the binding of bleomycin disaccharide to cell surface receptors is multivalent.^40,42,43^ Hence, the bleomycin saccharides may be potential targeting agents that would deliver oligonucleotide payloads to cancer cells,^39,44^ but the efficient enough targeting may need high multivalency.

**Scheme 1:**
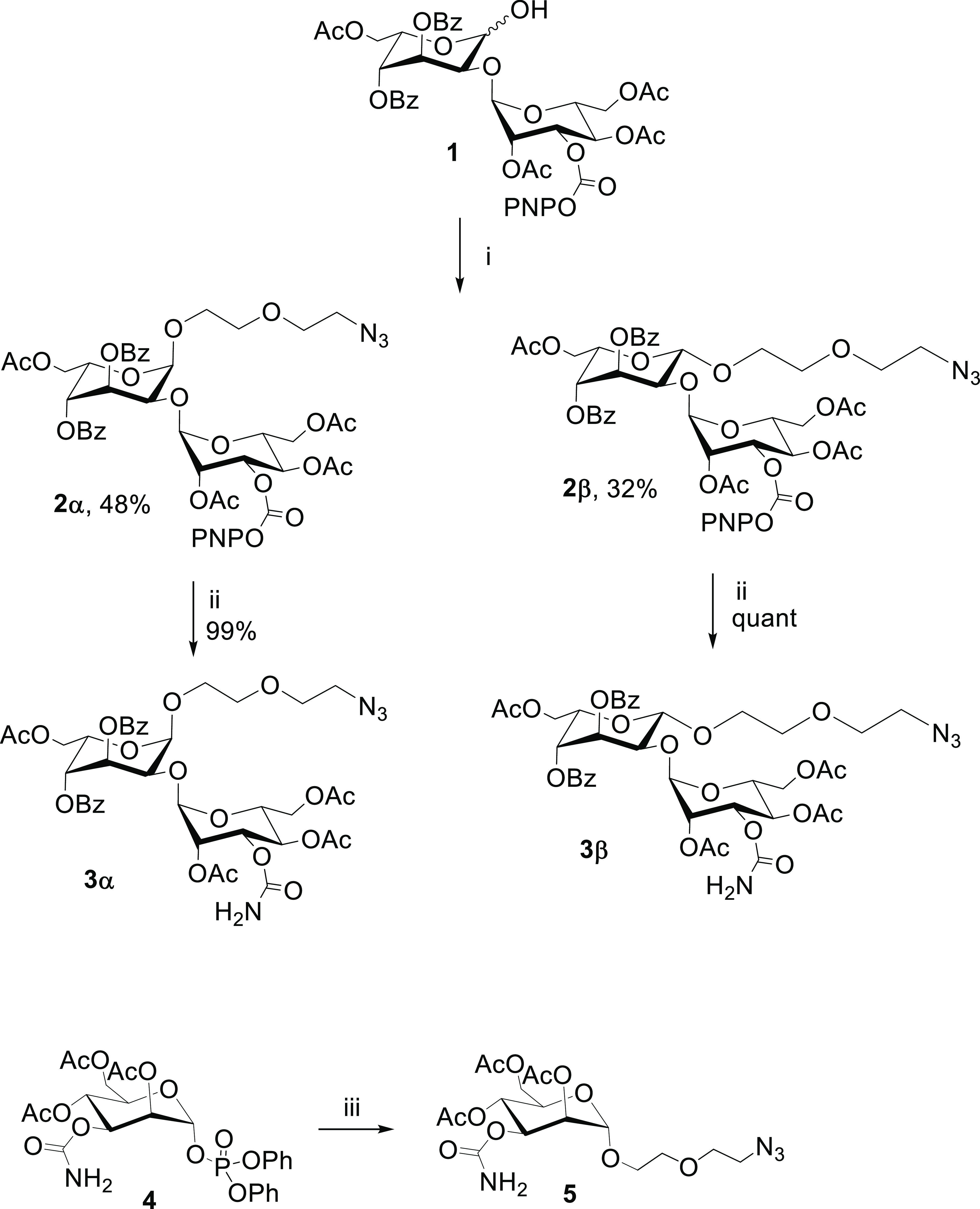
Synthesis of Azide-Modified Carbamoyl Mannose and Bleomycin Disaccharide Precursors Reagents and conditions: (i) Ph_2_SO, Tf_2_O, 2,4,6-tri-*tert*-butyl pyrimidine, 2-(2-azidoethoxy)ethanol, CH_2_Cl_2_, −60 °C to room temperature (rt); (ii) 0.4 N NH_3_ in tetrahydrofuran (THF), CH_2_Cl_2_; (iii) 2-(2-azidoethoxy)ethanol, TMSOTf, 0 °C.

**Scheme 2:**
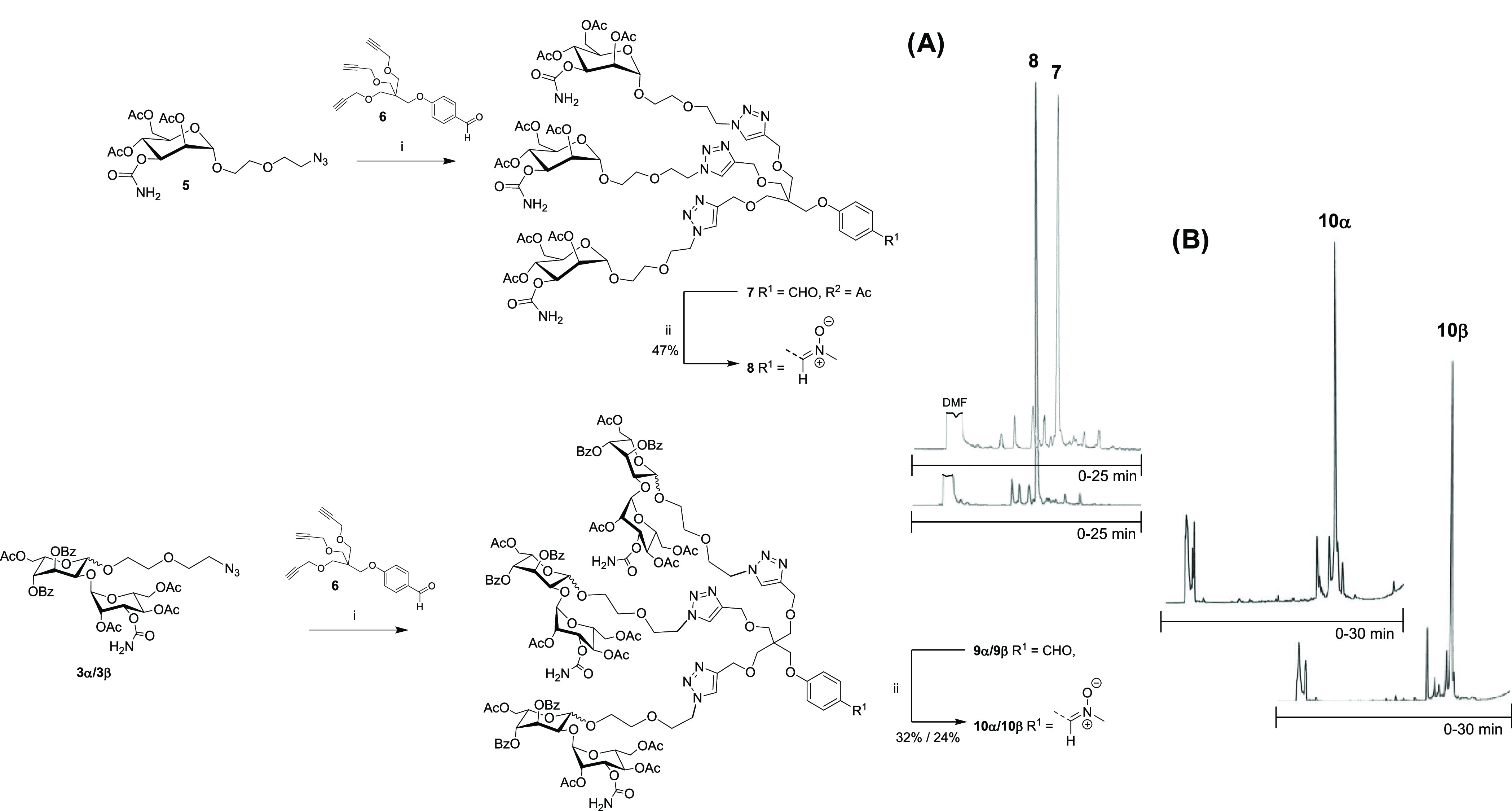
Synthesis of Trivalent Clusters of Carbamoyl Mannose and Bleomycin Disaccharide (A) Crude reversed-phase high-performance liquid chromatography (RP-HPLC) profiles of compounds **7** and **8**. (B) Crude RP-HPLC profiles of compounds **10α** and **10β**. Reagents and conditions: (i) sodium ascorbate, CuSO_4_, dioxane/H_2_O, overnight at 55 °C; (ii) *N*(Me)hydroxylamine hydrochloride, NaHCO_3_, dimethylformamide (DMF), 1 h at room temperature; RP-HPLC conditions: an analytical C-18 column (250 × 4.6 mm, 5 μm), detection at λ = 260 nm, flow rate: 1.0 mL min^–1^, a gradient elution from 25 to 100% MeCN in 0.01 mol L^–1^ aqueous triethylammonium acetate over 30 min.

**Scheme 3:**
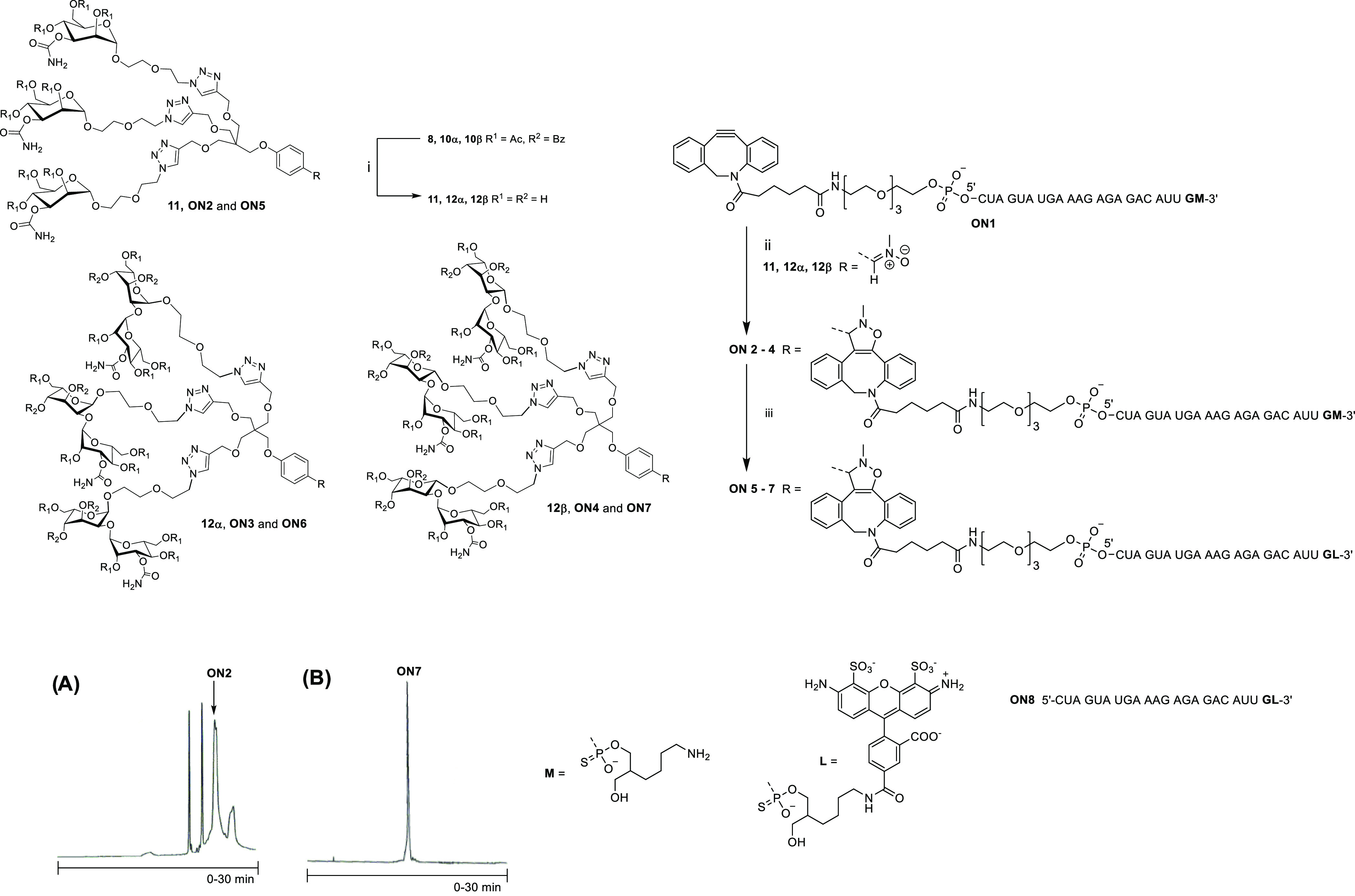
Synthesis of AF488-Labeled Glycocluster–Oligonucleotide Conjugates **ON5**–**7** The oligonucleotides are 2′-OMe-ribonucleotide phosphorothioates. (A) Crude RP-HPLC profile of **ON2**. (B) RP-HPLC profile of purified **ON7**. Reagents and conditions: (i) 7N NH_3_ in MeOH, 2 days at rt; (ii) **ON1** + **11**, **12α**, or **12β** (2 equiv), H_2_O, overnight at rt; (iii) AF488 *N*-hydroxysuccinimide ester (20 equiv in dimethyl sulfoxide, DMSO), 0.1 M sodium borate (aq, pH 8.5), overnight at room temperature. RP-HPLC conditions: (A) an analytical C-18 column (250 × 4.6 mm, 5 μm), detection at λ = 260 nm, gradient elution (0–25 min) from 0 to 50% MeCN in 0.1 M aqueous triethylammonium acetate, flow rate 1.0 mL min^–1^; (B) same as A, except gradient elution (0–25 min) from 5 to 95% MeCN in 0.1 M aqueous triethylammonium acetate.

**Scheme 4:**
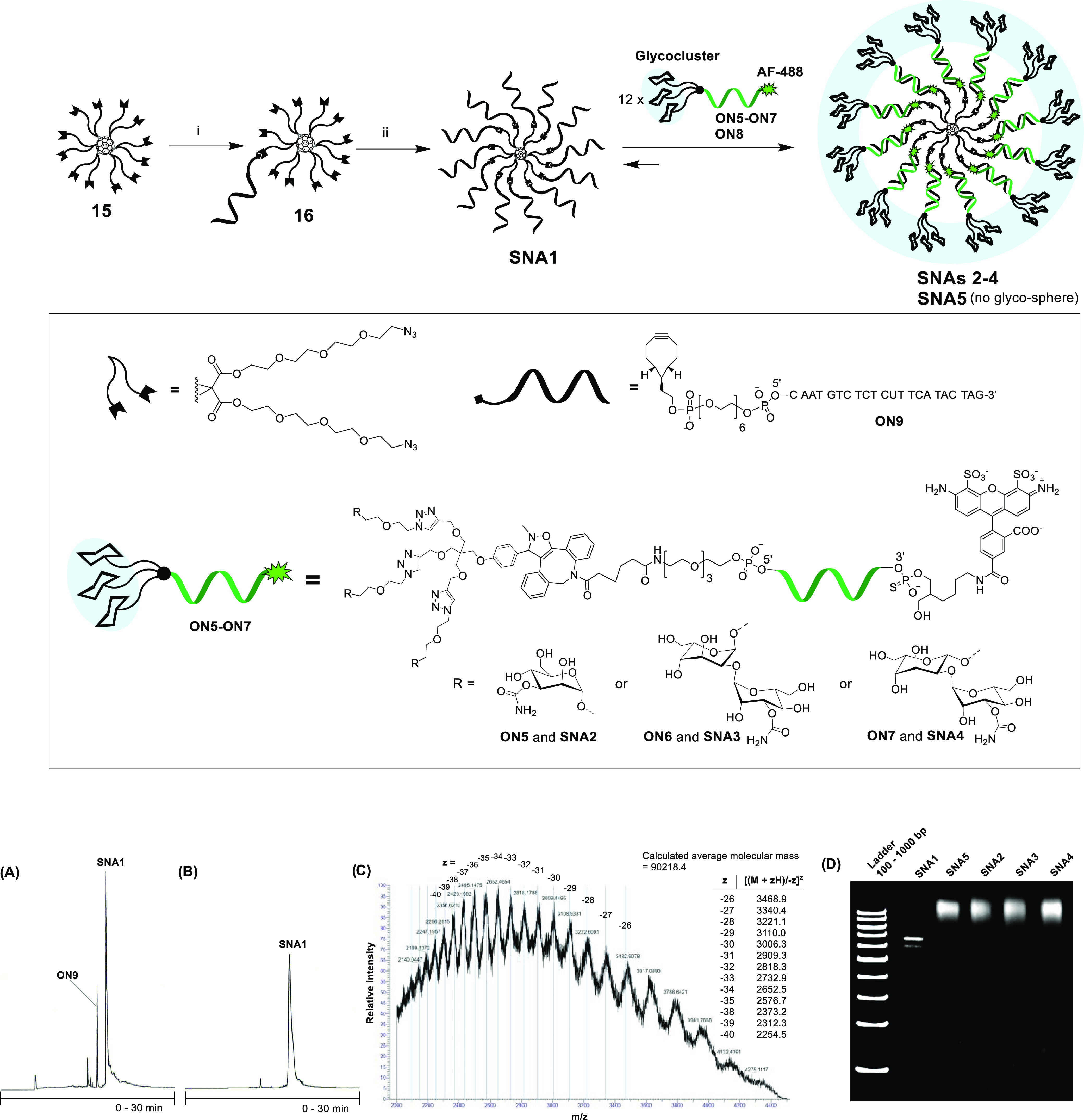
SNA Synthesis and Hybridization of AF488-Labeled Glycocluster–Oligonucleotide Conjugates **ON5**–**ON7** with Complementary **SNA1** (A) Crude RP-HPLC profile of **SNA1**. (B) RP-HPLC profile of purified **SNA1**. (C) Electrospray ionization mass spectrometry (MS-ESI) of **SNA1** (a spectrometer equipped with a hybrid quadrupole orbitrap and nano-ESI ionization). (D) Polyacrylamide gel electrophoresis (PAGE) of **SNAs 1**–**5**. Conditions: (i) **ON9** (0.3 equiv/compound **15**), DMSO/H_2_O 9:1 v/v, overnight at room temperature; (ii) **ON9** (1.2 equiv/azide arm of **16**), 1.5 M NaCl (aq), 3 days at room temperature; (A and B) An analytical RP-HPLC column Phenomenex, Aeris 3.6 μm WIDEPORE XB-C18 200 Å, 150 × 4.6 mm, linear gradient from 5 to 45% MeCN in 50 mmol L^–1^ triethylammonium acetate over 30 min, a flow rate of 1.0 mL min^–1^, detection at 260 nm; (D) for conditions, see the Experimental Section.

For the assembly of the glyco-decorated SNAs, azide-modified bleomycin disaccharide precursors (**3α**, **3β**, and **5**, Scheme 1) were synthesized and attached to an aldehyde-functionalized branching unit (**6**, Scheme 2) by Cu(I)-catalyzed 1,3-dipolar cycloaddition (“click” reaction) to obtain trivalent bleomycin disaccharide and carbamoyl mannose clusters (**8**, **10α**, and **10β**, Scheme 2). The aldehyde moiety of the clusters was converted into the reactive nitrone functional group and the acetyl and benzoyl groups of the sugar moieties were removed. The resulting nitrone-modified glycoclusters (**11**, **12α**, and **12β**, Scheme 3) were conjugated with 5′-dibenzo-bicyclo-octyne (DBCO)-modified 2′-O-methylated oligoribonucleotide AON_ARV7_ (**ON1**, Scheme 3) by strain-promoted alkyne-nitrone cycloaddition (SPANC).^45^ The conjugates were additionally labeled with a fluorescent dye (AF488, **ON5**–**ON7**, Scheme 3). A C_60_-based SNA^46^ bearing DNA strands with complementary sequence to AON_ARV7_ (**SNA1**, Scheme 4) was synthesized and hybridized with the glyco-AON_AR7_ conjugates (1:12 stoichiometry) to obtain the glyco-decorated SNAs (**SNA2**–**SNA4**, Scheme 4). The formation and stability of these glyco-decorated SNAs were evaluated by polyacrylamide gel electrophoresis (PAGE), UV melting profile analysis, and time-resolved fluorescence spectroscopy. The preliminary uptake of the glycocluster–oligonucleotide conjugates **ON5**–**ON7** and the corresponding glyco-decorated SNAs (**SNA2**–**4**) to human prostate cancer cells (PC3) was evaluated by confocal microscopy.

## Results and Discussion

### Synthesis of Azide-Modified Bleomycin Disaccharide Precursors

For the synthesis of azide-modified bleomycin disaccharide precursors **3α** and **3β** (Scheme 1), the previously reported 2-*O*-[2,4,6-tri-*O*-acetyl-3-*O*-(*p*-nitrophenylformyl)-α-d-mannopyranosyl]-3,4-di-*O*-benzoyl-6-*O*-acetyl-l-gulopyranose (**1**)^44^ was glycosylated with 2-(2-azidoethoxy)ethanol using *in situ* formation of a sulfoxide donor in the presence of trifluoromethanesulfonic anhydride, diphenyl sulfoxide, and 2,4,6-tri-*tert*-butyl pyridine. Compound **2** was obtained as pure α- and β-anomers in 48 and 32% yields, respectively (overall yield 80%). Finally, compounds **2α** and **2β** were quantitatively converted into **3α** and **3β** using 0.4 N ammonia in tetrahydrofurane, followed by filtration through a short silica column. The azide-modified carbamoyl mannose precursor **5**, on the other hand, was synthesized from the previously reported^47^ diphenylphosphate-activated carbamoyl mannose **4** by treatment with 2-(2-azidoethoxy)ethanol in the presence of trimethylsilyl trifluoromethanesulfonate.

### Synthesis of Nitrone-Modified Trivalent Clusters of Carbamoyl Mannose and Bleomycin Disaccharide

For the synthesis of trivalent glycoclusters, the azide-modified carbamoyl mannose and bleomycin disaccharide precursors **3α**, **3β**, and **5** were connected to the branching unit **6** by Cu(I)-catalyzed 1,3-dipolar cycloaddition (5 equiv sugar azide vs **6** in dioxane-H_2_O, overnight at 55 °C, Scheme 2). The aldehyde moieties of the resulting compounds **7**, **9α**, and **9β** were then converted *in situ* into reactive nitrone functional groups by treatment with *N*-methylhydroxylamine hydrochloride. The resulting nitrone-modified glycoclusters **8**, **10α**, and **10β** were purified by RP-HPLC in 47, 32, and 24% isolated yields, respectively. The authenticity of the products was confirmed by mass and NMR spectroscopy.

### Synthesis of the Bleomycin Saccharide-Oligonucleotide Conjugates

Prior to conjugation with AON_ARV7_, the glycoclusters were globally deprotected by 7N ammonia in methanol (Scheme 3). The authenticity of the crude products (**11**, **12α**, and **12β**) was verified by MS, and their applicability for strain-promoted alkyne-nitrone cycloaddition (SPANC) was confirmed with a commercially available dibenzobicyclooctyne (DBCO)-modified rhodamine dye. After this successful small-molecule trial (see compounds **13**, **14α**, and **14β** in the Supporting Information), 5′-DBCO- and 3′-amino-modified 2′-*O*-methylated phosphorothioate oligoribonucleotide AON_ARV7_ (**ON1**) was synthesized by an automated synthesizer and exposed to SPANC with **11**, **12α**, and **12β** (2 equiv each vs **ON1**, overnight at rt). The product mixtures were purified by RP-HPLC (Scheme 3A) to yield the glycocluster–AON_ARV7_ conjugates **ON2**, **ON3**, and **ON4** in 17, 11, and 15% isolated yields, respectively. The resulting glycocluster–AON_ARV7_ conjugates **ON2**–**ON4** were then labeled with Alexa (AF488) fluorescent dye NHS ester using a well-established procedure^48^ to yield the labeled conjugates **ON5**, **ON6**, and **ON7** in ca. 20% isolated yields. The authenticity of the products was verified by MS (orbitrap) spectroscopy.

### SNA Synthesis

Fullerene C_60_-based **SNA1** with twelve 2′-deoxy oligoribonucleotide sequences, complementary to AON_ARV7_ was synthesized using strain-promoted alkyne-azide cycloaddition (SPAAC) and following the procedure reported previously by our group.^49^ Due to the solubility issues, the azide-functionalized C_60_ core **15**(46,49) was first monosubstituted using a substoichiometric amount (0.3 equiv) of bicyclo[6,1,0]nonyne (BCN)-modified oligonucleotide (**ON9**) in DMSO (Scheme 4). The monosubstituted product (**16**) was then exposed to an excess of the same oligonucleotide in aqueous medium and high salt concentration. This two-step process alleviated the solubility issues and yielded a more homogeneous product. The homogeneity of **SNA1** was confirmed by RP-HPLC and PAGE, and its authenticity was confirmed by MS (a spectrometer equipped with a hybrid quadrupole orbitrap and nano-ESI ionization; Scheme 4A–D). The particle size of **SNA1** (in 100 μL aqueous 10 mmol L^–1^ phosphate-buffered saline (PBS), 1.1 mmol L^–1^ M KCl, 0.154 mol L^–1^ NaCl, pH 7) was evaluated by dynamic light scattering (DLS) that showed a hydrodynamic diameter of 10.6 ± 0.2 nm.

### Formation and Stability of Hybridization-Mediated **SNAs 2**–**5**

On the gel electrophoresis, **SNA1** alone resulted in a distinct and relatively sharp band (Scheme 4D). A trace of a faster eluting side product was also observed (may refer to an 11-armed SNA),^49^ but the overall purity of **SNA1** after single RP-HPLC purification proved high. Mixtures of **SNA1** with **ON5**–**8** [12 equiv, samples prepared in phosphate-buffered saline (PBS) at pH 7.4] resulted in slower eluting broad bands on the gel representing the formation of the hybridization-mediated SNAs: **SNA2**–**SNA5**. We also evaluated the particle size of the hybridization-mediated SNAs by DLS in 100 μL of aqueous 10 mmol L^–1^ PBS, 1.1 mmol L^–1^ M KCl, 0.154 mol L^–1^ NaCl, pH 7.4. Hydrodynamic diameters with relatively large error limits were obtained: **SNA5**: 11.5 ± 1.1 nm, **SNAs 2**–**4**: 13.8 ± 1.7 nm (no marked difference between the different glyco decorations).

The UV thermal melting temperatures were next measured using 0.083 μmol L^–1^**SNA1** and 12 equiv (1.0 μmol L^–1^) of **ON5**–**8** in 10 mmol L^–1^ sodium cacodylate buffer (pH 7.0) with 0.1 mol L^–1^ NaCl (Table 1). The UV melting curves showed inflection points at 56–57 °C. Compared to nonconjugated oligonucleotide **ON8**, the glycocluster moieties of **ON5**–**7** decreased the melting temperature of the SNA/oligonucleotide duplex slightly: 1.1–1.6 °C.

**Table 1:** UV Thermal Melting Temperatures of the SNA1/AON_ARV7_ Complexes^a^

	*T*_m_/°C
**SNA1** + 12 equiv **ON8**	57.8 ± 0.9
**SNA1** + 12 equiv **ON5**	56.2 ± 0.6 (−1.6)
**SNA1** + 12 equiv **ON6**	56.3 ± 0.6 (−1.5)
**SNA1** + 12 equiv **ON7**	56.7 ± 1.2 (−1.1)

a Conditions: 0.083 μmol L^–1^ SNA + 12 equiv (1.0 μmol L^–1^) of the AON_ARV7_ conjugates **ON5**–**8**, 10 mmol L^–1^ sodium cacodylate (pH 7.0), 0.1 mol L^–1^ NaCl in H_2_O. Detection wavelength 260 nm. Δ*T*_m_ values in parentheses are compared to SNA + 12 equiv **ON8**.

The hybridization between **SNA1** and the AF488-labeled oligonucleotides **ON5**–**8** was studied in more detail by fluorescence spectroscopy. The fluorescence properties of the **ON5**–**8** and **SNA1** complexes are presented in Table S3. The presence of the glycocluster moieties in **ON5**–**7** clearly influenced the spectral properties of AF488. For **ON6**–**7**, the absorption maximum was shifted 3 nm to the red compared with that of **ON5** and **ON8**. Thus, it seems that AF488 interacts with α- and β-bleomycin moieties in the ground state. The fluorescence intensity increased during complexation (Figures S62 and S63), but so did the absorbance as the amount of AF488 in the samples increased. Determining the change in absorption for the 50 μL samples especially at low oligonucleotide amounts resulted in large errors. However, the fluorescence lifetimes do not depend on the dye concentration and thus the association constants were determined from the time-resolved data. For all glycocluster derivatives **ON5**–**7**, the fluorescence decay curves were one-exponential, whereas for **ON8**, they were two-exponential (Figure S64). In the presence of **SNA1**, the decays of **ON5**–**7** became two-exponential due to the complex formation with **SNA1**. The average fluorescence lifetime ⟨τ⟩ (Table S2) decreased during complexation for all oligonucleotides (Figure S65). For **ON8** and **ON5**, the decrease was due to the increase in the proportion of the short-living component, whereas for **ON6**–**7**, both the lifetime and the proportion of the short-living component changed during complexation. The association constants were determined by plotting the ⟨τ⟩ as a function of inverse oligonucleotide concentration (Figure S65). For carbamoyl mannose conjugate **ON5**, the association constant was nearly equal to that obtained for **ON8**, whereas for α- and β-bleomycin disaccharide conjugates **ON6** and **ON7**, the association constant was about half of that for **ON5** and **ON8** (Table 2). For all of the oligonucleotides, the complexation seemed to be complete at a 12:1 oligonucleotide/SNA ratio. For the association constants determined from the time-resolved data, only ratios 4–12:1 or 8–12:1 could be used. This could indicate that the oligonucleotides first bind to different parts of the SNA. Only when the oligonucleotides start to bind to the adjacent strands of already filled positions, the AF488 fluorescence is quenched, and this can be observed as the decrease in the ⟨τ⟩. For **ON6**–**7**, the quenching starts at higher ratios than for **ON8** and **ON5**, due to the stronger interaction of AF488 with **ON6**–**7** sugar moieties.

**Table 2:** Association Constants for the SNA1/AON_ARV7_ Complexes^a^

	*K*_assoc_/M^–1^ (equivalents of ON/SNA)
**ON8**	5.18 × 10^6^ (4–12)
**ON5**	4.64 × 10^6^ (4–12)
**ON6**	2.05 × 10^6^ (8–12)
**ON7**	2.24 × 10^6^ (8–12)

a Conditions: 0.1 μM **SNA1** in Dulbecco’s phosphate-buffered saline (DPBS) was titrated with 10 μM **ON5**–**ON8** solutions. λ_ex_ = 483 nm and decays were monitored at 500–570 nm. Numbers in parentheses refer to oligonucleotide-SNA ratios used to extract the association constants.

**Figure 1:**
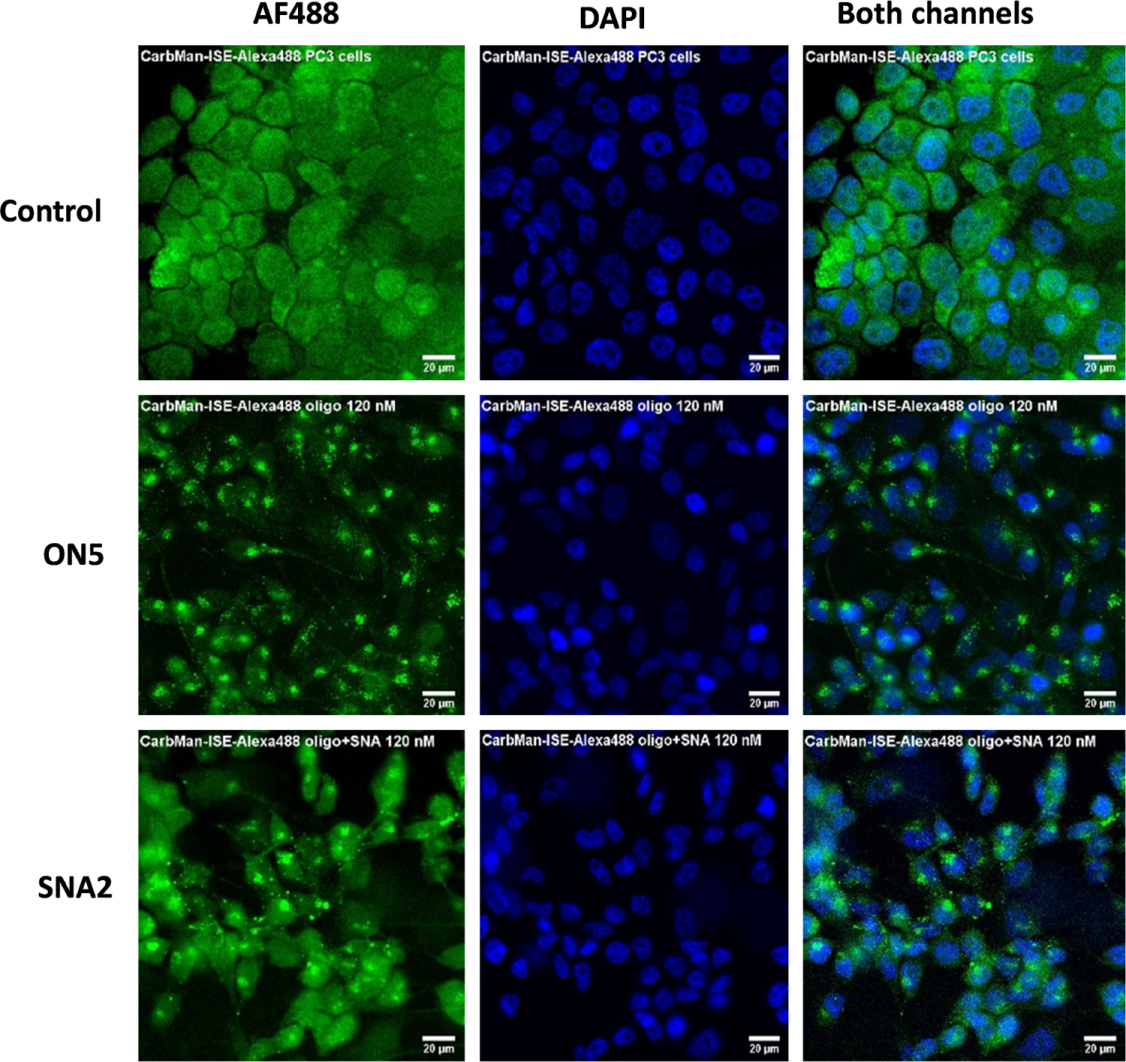
Uptake of AF488-labeled carbamoyl mannose conjugate **ON5** and **SNA2** by PC3 cells (Note: the scale bar is 20 μm). The cells were incubated (37 °C with 5% CO_2_) with 120 nM oligonucleotide and 10 nM SNA concentrations or in PBS (controls) for 4 h. After incubation, the cells were washed three times with PBS and fixed using 4% paraformaldehyde solution (diluted in PBS). The intracellular delivery of oligonucleotides and SNAs was quantified via wide-field microscopy on a Nikon Eclipse Ti2-E microscope using Alexa 488 (475 nm) and 4′,6-diamidino-2-phenylindole (DAPI) (395 nm) channels and normalized based on the intensity of the untreated control cell.

### Cell Uptake Studies

The cell uptake studies of all of **ON5**–**8** (120 nM) and the corresponding **SNAs 2**–**5** (10 nM, i.e., the total oligonucleotide concentration was the same in each experiment) were carried out with PC3 cells, and the obtained data were compared with the untreated PC3 cells as controls. The incubation time of **ON5**–**ON8** and **SNAs 2**–**5** with the PC3 cells was about 4 h, after which the cells were labeled and fixed for the confocal and wide-field microscopy examination. Figure 1 presents, as an example, the obtained cell uptake data of **ON5** and **SNA2**, and the others are shown in Figures S67–S69. Based on these data, it is clearly shown that **ON5**–**8** and the corresponding SNAs are taken up by the PC3. It also could be stated that the oligonucleotides with the **SNA1** carrier are taken up by the cells more effectively than those without the carrier. In this primarily synthetic technical report, PC3 cell line was selected for the preliminary cell uptake study. A more detailed mechanistic study of the internalization and intracellular trafficking in 22RV1 prostate cancer cells (overexpressing AR-V7), together with PNT2 health prostate cells as controls, is underway in our laboratory.

## Conclusions

Tripodal clusters of bleomycin disaccharide and carbamoyl mannose were synthesized and conjugated with an antisense oligonucleotide. The oligonucleotide conjugates were labeled with a fluorescent dye and hybridized with complementary strands of a C_60_-based molecular SNA to form SNA structures surrounded by a carbohydrate sphere. The formation and stability of these hybridization-mediated glyco-decorated SNAs were evaluated by PAGE, UV melting profile analysis, and time-resolved fluorescence spectroscopy. The melting temperatures (ca. 56 °C) and the association constants (2.1–4.6 × 10^6^ L mol^–1^), extracted from the time-resolved fluorescence spectroscopy, confirmed that the complex formation of these hybridization-mediated SNAs was favored under physiological conditions. Preliminary cell uptake experiments showed that the glyco-oligonucleotide conjugates (120 nM solutions) and the corresponding glyco-decorated SNAs (10 nM solutions) were efficiently taken up by prostate cancer cells (PC3). The results indicated that the glyco-decorated SNAs were taken up by the cells more efficiently than the corresponding glyco-oligonucleotide conjugates. Furthermore, variation in the intracellular distribution was noticed. A more detailed mechanistic study of internalization and intracellular trafficking is currently underway in our laboratory.

## Experimental Section

### General Remarks

RP-HPLC analysis and purification of the oligonucleotides and oligonucleotide conjugates were performed using Thermo ODS Hypersil C18 (250 × 4.6 mm, 5 μm) analytical column. The mass spectra were recorded using either an MS (orbitrap) or MS electrospray ionization time-of-flight (ESI-TOF) spectrometer. The isolated yields of the oligonucleotide conjugates were determined according to their UV absorbance at 260 nm and 495 nm (AF488-labeled oligonucleotides).

#### 2-(2-Azidoethoxy)ethyl-2-*O*-{2,4,6-tri-*O*-acetyl-3-O-[(*p*-nitrophenyl)formyl]α-d-mannopyranosyl}-3,4-di-*O*-benzoyl-6-*O*-acetyl-α-l-gulopyranoside (**2α**) and 2-(2-Azidoethoxy)ethyl-2-*O*-{2,4,6-tri-*O*-acetyl-3-O-[(*p*-nitrophenyl)formyl]α-d-mannopyranosyl}-3,4-di-*O*-benzoyl-6-*O*-acetyl-β-l-gulopyranoside (**2β**)

Trifluoromethanesulfonic anhydride (76 μL, 0.45 mmol) was added to a suspension of compound 1 (280 mg, 0.32 mmol), diphenyl sulfoxide (180 mg, 0.90 mmol), 2,4,6-tri-*tert*-butyl pyrimidine (240 mg, 2.7 mmol), and 4 Å molecular sieves in dichloromethane (10 mL) at −60 °C under argon. The reaction mixture was stirred at this temperature for 5 min and then at −40 °C for 1.5 h. 2-(2-Azidoethoxy)ethanol (84 mg, 0.64 mmol) was added at −40 °C. The solution was stirred at this temperature for 1 h, and then the temperature was allowed to increase slowly to room temperature for 3 h. The reaction was quenched with excess triethylamine and filtered through a Celite bed, and the bed was washed with dichloromethane (20 mL). The filtrate was washed sequentially with saturated aqueous sodium bicarbonate solution (50 mL) and saturated aqueous sodium chloride solution (50 mL). The organic layer was dried over sodium sulfate and concentrated, and the residue was purified by silica gel flash column chromatography (30% EtOAc-toluene) to afford **2α** (150 mg, 48%) and **2β** (100 mg, 32%) as colorless syrups.

**2α**: ^1^H NMR (500 MHz, CDCl_3_) δ 2.01 (s, 3 H, COC*H*_3_), 2.07 (s, 3 H, COC*H*_3_), 2.11 (s, 3 H, COC*H*_3_), 2.14 (s, 3 H, COC*H*_3_), 3.20–3.30 (m, 2 H, CH_2_N_3_), 3.54–3.58 (m, 1 H, OCH_2_), 3.66–3.75 (m, 3 H, OCH_2_), 3.79–3.84 (m, 1 H, OCH_2_), 4.01–4.06 (m, 1 H), 4.09–4.13 (m, 2 H, H-5′, H-6), 4.20–4.24 (m, 2 H, H-2, H-6′), 4.26–4.33 (m, 2 H, H-6′), 4.73 (m, 1 H, H-5), 5.05 (dd, *J* = 3.5, 10.0 Hz, 1 H, H-3′), 5.10 (d, *J* = 4.0 Hz, 1 H, H-1), 5.15 (d, *J* = 1.5 Hz, 1 H, H-1′), 5.33 (t, *J* = 10.0 Hz, 1 H, H-4′), 5.38 (dd, *J* = 1.5, 3.0 Hz, 1 H, H-2′), 5.44 (d, *J* = 3.0 Hz, 1 H, H-4), 5.70 (t, *J* = 3.5 Hz, 1 H, H-3), 7.31–7.34 (m, 2 H, PNP), 7.43–7.50 (m, 4 H, Bz), 7.57–7.64 (m, 2 H, Bz), 8.06–8.07 (m, 2 H, Bz), 8.13–8.17 (m, 2 H, Bz), 8.24–8.27 (m, 2 H, PNP); ^13^C NMR (125 MHz, CDCl_3_) δ 20.72, 20.74, 20.8, 50.6, 62.3, 64.2, 65.6, 66.0, 68.2, 68.4, 69.10, 69.14, 70.3, 71.0, 74.2, 97.1 (C-1′, *J*_C–H_ = 174.4 Hz), 97.4 (C-1, *J*_C–H_ = 170.1 Hz), 121.9, 125.2, 128.3, 128.7, 128.9, 129.5, 129.7, 129.9, 130.3, 133.4, 133.9, 145.5, 151.2,155.3, 165.0, 165.4, 169.6, 169.8,170.5, 170.6; HRMS calcd for C_45_H_48_N_4_O_22_Na (M + Na)^+^: 1019.2658, found: 1019.2634.

**2β**: ^1^H NMR (500 MHz, CDCl_3_) δ 2.03 (s, 3 H, COC*H*_3_), 2.08 (s, 3 H, COC*H*_3_), 2.11 (s, 3 H, COC*H*_3_), 2.16 (s, 3 H, COC*H*_3_), 3.24–3.33 (m, 2 H, CH_2_N_3_), 3.60–3.74 (m, 4 H, OCH_2_), 3.83–3.87 (m, 1 H, OCH_2_), 4.03–4.07 (m, 2 H, H-2 and OCH_2_), 4.20 (dd, *J* = 2.5, 12.5 Hz, 1 H, H-5′), 4.23–4.32 (m, 4 H, H-6 and H-6′), 4.41 (m, 1 H, H-5′), 4.47 (m, 1 H, H-5), 4.97 (d, *J* = 7.5 Hz, 1 H, H-1), 5.04 (dd, *J* = 3.5, 10.0 Hz, 1 H, H-3′), 5.11 (d, *J* = 1.0 Hz, 1 H, H-1′), 5.32 (dd, *J* = 2.0, 3.5 Hz, 1 H, H-2′), 5.34 (t, *J* = 10.0 Hz, 1 H, H-4′), 5.39 (dd, *J* = 1.5, 4.0 Hz, 1 H, H-4), 5.80 (t, *J* = 3.5 Hz, 1 H, H-3), 7.29–7.34 (m, 2 H, PNP), 7.46–7.50 (m, 4 H, Bz), 7.60–7.65 (m, 2 H, Bz), 8.07–8.08 (m, 4 H, Bz), 8.24–8.29 (m, 2 H, PNP); ^13^C NMR (125 MHz, CDCl_3_) δ 20.85, 20.89, 20.9, 20.94,50.6, 61.9, 62.2, 65.5, 66.5, 68.2, 68.6, 68.7, 69.0, 70.1, 70.8, 70.9,71.4, 74.8, 95.4 (C-1′, *J*_C–H_ = 175.7 Hz), 99.5 (C-1, *J*_C–H_ = 163.2 Hz), 122.0, 125.3, 128.7, 128.8, 129.0, 130.1, 130.2,132.0, 133.9, 134.0, 145.6, 151.5, 155.5, 165.2, 165.3, 169.7, 170.0, 170.6, 170.9.

#### 2-(2-Azidoethoxy)ethyl-2-*O*-{2,4,6-tri-*O*-acetyl-3-*O*-carbamoyl-α-d-mannopyranosyl}-3,4-di-*O*-benzoyl-6-*O*-acetyl-α-l-gulopyranoside (**3α**)

To a stirred solution of 2-(2-azidoethoxy)ethyl-2-*O*-{2,4,6-tri-*O*-Acetyl-3-O-[(*p*-nitrophenyl)formyl]α-d-mannopyranosyl}-3,4-di-*O*-benzoyl-6-*O*-acetyl-α-l-gulopyranoside (54 mg, 0.054 mmol) in dry CH_2_Cl_2_ (2 mL) was added 0.4 N solution of ammonia in tetrahydrofuran (2 mL). After stirring for 2 h at room temperature, the solvents were evaporated *in vacuo*. Flash column chromatographic purification of the residue yielded **3α** (47 mg, 99%) as a colorless syrup; ^1^H NMR (500 MHz, CDCl_3_) δ 2.00 (s, 3 H, COC*H*_3_), 2.03 (s, 3 H, COC*H*_3_), 2.09 (s, 3 H, COC*H*_3_), 2.10 (s, 3 H, COC*H*_3_),3.25–3.29 (m, 1 H, CH_2_N_3_), 3.33–3.38 (m, 1 H, CH_2_N_3_), 3.59–3.63 (m, 1 H, OCH_2_), 3.71–3.78 (m, 3 H, OCH_2_), 3.82–3.86 (m, 1 H, OCH_2_), 4.01–4.30 (m, 6 H, H-5′, H-6, H-6′ and H-2), 4.58 (bs, 2 H, NH_2_), 4.72 (t, 1 H, *J* = 6.0 Hz, H-5), 5.10–5.13 (m, 3 H, H-1′, H-1 and H-3′), 5.18 (m, 1 H, H2′), 5.22 (t, 1 H, *J* = 10.0 Hz, H4′), 5.44 (m, 1 H, H-4), 5.68 (t, 1 H, *J* = 4.0 Hz, H-3), 7.48 (m, 4 H, Bz), 7.59–7.63 (m, 2 H, Bz), 8.06 (m, 2 H, Bz), 8.16 (m, 2 H, Bz); ^13^C NMR (125 MHz, CDCl_3_) δ 20.7, 20.9, 50.7, 62.4, 62.5, 64.2, 66.1, 66.3, 68.3, 69.1, 69.2, 69.6, 69.7, 70.3, 70.7, 96.9, 97.5, 128.4, 128.7, 129.0, 129.6, 129.9, 130.3, 133.4, 133.8, 154.8, 165.0, 165.3, 169.5, 169.9, 170.5, 170.6; HRMS calcd for C_39_H_46_N_4_O_19_Na (M + Na)^+^: 897.2654, found: 897.2642.

#### 2-(2-Azidoethoxy)ethyl-2-*O*-{2,4,6-tri-*O*-acetyl-3-*O*-carbamoyl-α-d-mannopyranosyl}-3,4-di-*O*-benzoyl-6-*O*-acetyl-β-l-gulopyranoside (**3β**)

To a stirred solution of 2-(2-azidoethoxy)ethyl-2-*O*-{2,4,6-tri-*O*-acetyl-3-O-[(*p*-nitrophenyl)formyl]α-d-mannopyranosyl}-3,4-di-*O*-benzoyl-6-*O*-acetyl-β-l-gulopyranoside (100 mg, 0.10 mmol) in dry CH_2_Cl_2_ (5 mL) was added 0.4 N solution of ammonia in tetrahydrofuran (5 mL). After stirring for 2 h at room temperature, the solvents were evaporated *in vacuo*. Flash column chromatographic purification of the residue yielded **3β** (89 mg, 99%) as a colorless syrup; ^1^H NMR (500 MHz, CDCl_3_) δ 2.02 (s, 3 H, COC*H*_3_), 2.03 (s, 3 H, COC*H*_3_), 2.08 (s, 3 H, COC*H*_3_), 2.12 (s, 3 H, COC*H*_3_), 3.26–3.36 (m, 2 H, CH_2_N_3_), 3.61–3.66 (m, 1 H, OCH_2_), 3.68–3.72 (m, 3 H, OCH_2_), 3.85–3.89 (m, 1 H, OCH_2_), 4.03–4.07 (m, 2 H, OCH_2_ and H-2), 4.15–4.30 (m, 4 H, H-6, H-6′), 4.38–4.41 (m, 1 H, H-5′), 4.45 (t, 1 H, *J* = 6.5 Hz, H-5), 4.63 (bs, 2 H, NH_2_), 5.00 (d, 1 H, *J* = 8.0 Hz, H-1), 5.06 (bs, 1 H, H-1′), 5.09–5.11 (m, 2 H, H-2′ and H-3′), 5.23 (t, 1 H, *J* = 10.0 Hz, H-4′), 5.37 (d, 1 H, *J* = 3.0 Hz, H-4), 5.77 (t, 1 H, *J* = 4.0 Hz, H-3), 7.45–7.52 (m, 4 H, Bz), 7.60–7.64 (m, 2 H, Bz), 8.07 (m, 4 H, Bz); ^13^C NMR (125 MHz, CDCl_3_) δ 20.7, 20.8, 20.9, 50.6, 62.0, 62.2, 66.1, 66.2, 68.0, 68.62, 68.65, 68.73,70.0, 70.1, 70.7, 70.8, 70.9, 95.1, 99.5, 128.6, 128.7, 128.8, 129.0, 129.04, 130.0, 133.7, 133.9, 155.0, 165.0, 165.04, 169.4, 169.9, 170.5, 170.7. HRMS calcd for C_39_H_46_N_4_O_19_Na (M + Na)^+^: 897.2654, found: 897.2642.

#### 2-(2-Azidoethoxy)ethyl-2,4,6-tri-*O*-acetyl-3-*O*-carbamoyl-α-d-mannopyranoside (**5**)

A solution of compound **4** (370 mg, 0.64 mmol) and 2-(2-azidoethoxy)ethanol (100 mg, 0.77 mmol) in dry CH_2_Cl_2_ (5 mL) was treated with TMSOTf (230 μL, 1.3 mmol) at 0 °C. The reaction mixture was stirred at 0 °C for 10 min and then poured into a two-phase solution of EtOAc (25 mL) and saturated aqueous NaHCO_3_ (50 mL) with vigorous stirring. The organic layer was washed with saturated aqueous NaCl, dried over anhydrous Na_2_SO_4_, and concentrated in *vacuo*. Flash chromatography over silica gel using 50% EtOAc-hexane gave **5** (220 mg, 75%) as a syrup; ^1^H NMR (500 MHz, CDCl_3_) δ 2.05 (s, 3 H, COC*H*_3_), 2.09 (s, 3 H, COC*H*_3_), 2.14 (s, 3 H, COC*H*_3_), 3.38 (m, 2 H, CH_2_N_3_), 3.65–3.66 (m, 5 H, OCH_2_), 3.81 (m, 1 H, OCH_2_), 4.07–4.10 (m, 2 H, H-5 and H-6), 4.28 (dd, 1 H, *J* = 5.0, 12.5 Hz, H-6), 4.84 (bs, 2 H, NH_2_), 4.88 (s, 1 H, H-1), 5.25–5.27 (m, 3 H, H-2, H-3 and H-4); ^13^C NMR (125 MHz, CDCl_3_) δ 20.8, 21.0, 50.7, 62.4, 66.2, 67.3, 68.4, 69.97, 69.98, 70.0, 70.2, 97.7 (*J*_C–H_ = 174.5 Hz), 155.4, 170.0, 170.7; HRMS calcd for C_17_H_26_N_4_O_11_K (M + K)^+^: 501.1235, found: 501.1242.

### Synthesis of Nitrone-Modified Trivalent Clusters of Carbamoyl Mannose and Bleomycin Disaccharide (Compounds **8**, **10α**, and **10β**)

Compounds **5** (21 mg, 45 μmol) and **6** (3.0 mg, 8.5 μmol) were dissolved in dioxane (60 μL) and mixtures of 0.1 mol L^–1^ sodium ascorbate (40 μL, 4.0 μmol) and 0.1 mol L^–1^ CuSO_4_ (2.0 μL, 0.20 μmol) were added. The mixture was mixed overnight at 55 °C, poured to saturated NaHCO_3_ (100 μL), and extracted with EtOAc (3 × 300 μL). The organic layers, containing the product, were combined, dried over Na_2_SO_4_, and evaporated to dryness. RP-HPLC analysis (sample dissolved in DMF, a gradient elution from 25 to 100% MeCN in 0.01 mol L^–1^ triethylammonium acetate over 25 min, an analytical RP C-18 column) confirmed that relatively pure crude product **7** (23 mg) was obtained (Scheme 2A). HRMS (ESI-TOF): *m*/*z* C_72_H_101_N_12_O_38_ [M + H]^+^ requires 1741.63, found 1741.64. The crude product (**7**, 23 mg), *N*(Me)hydroxylamine hydrochloride (1.8 mg, 22 μmol), and NaHCO_3_ (3.0 mg, 35 μmol) were dissolved in DMF (100 μL). The mixture was mixed for 1 h at rt and purified by RP-HPLC (Scheme 2A, the same eluent system as used above). The product fractions were lyophilized to give 7.0 mg (4.0 μmol, 47% overall yield from **6**) of the homogenized oxime product **8** as white foam. ^1^H NMR (500 MHz, CD_3_OD) δ 1.96 (s, 9 H, COCH_3_), 1.97 (s, 9 H, COCH_3_), 2.05 (s, 9 H, COCH_3_), 3.49 (s, 6 H, CH_2_, pentaerythritol), 3.54 (b, 9 H, OCH_2_), 3.69–3.72 (m, 3 H, OCH_2_), 3.75 (s, 3 H, NCH3), 3.81 (m, 6 H, OCH_2_), 3.88 (s, 2 H, CH2OPh), 3.94 (m, 3 H, H-5), 4.00 (dd, 3 H, *J* = 2.3 Hz, 12.2 Hz, H-6), 4.13 (dd, 3 H, *J* = 5.1 Hz, 12.3 Hz, H-6), 4.47 (b, 12H), 4.76 (s, 3 H, H-1), 5.05 (dd, 3 H, *J* = 3.5 Hz, 10.2 Hz, H-3), 5.13–5.17 (m, 6 H, H-2 and H-4), 6.86 (d, 2 H, *J* = 8.9 Hz, Ph), 7.68 (s, 1 H, CHN), 7.83 (s, 3 H, triazol), 8.14 (d, 2 H, *J* = 8.9 Hz, Ph); ^13^C NMR (125 MHz, CD_3_OD) δ 44.8, 50.0, 52.1, 62.3, 63.8, 66.3, 66.5, 66.9, 68.0, 68.5, 69.0, 69.3, 69.7, 69.8, 97.5, 114.2, 122.9, 124.3, 131.3, 138.3, 14.5, 156.7, 161.4, 170.1, 170.3, 171.0; HRMS (ESI-TOF): *m*/*z* C_73_H_104_N_13_O_38_ [M + H]^+^ requires 1770.66, found 1770.63. The NMR spectra of compound **8** are presented in Figures S34–S37.

Compounds **10α** and **10β** were synthesized following the same procedure described for compound **8**. The NMR spectra of compounds **10α** and **10β** are presented in Figures S38–S56. **10α**: 8.2 mg (2.7 μmol, 32% overall yield from **6**). HRMS (ESI-TOF): *m*/*z* C_139_H_164_N_13_KO_62_^2+^ [(M + H + K)/2]^2+^ requires 1533.9763, found 1533.9573. **10β**: 6.1 mg (2.0 μmol, 24% overall yield from **6**). HRMS (ESI-TOF): *m*/*z* C_139_H_164_N_13_KO_62_^2+^ [(M + H + K)/2]^2+^ requires 1533.9763, found 1533.9710.

### Global Deprotection of Glycoclusters (Removal of Acetyl and Benzoyl Protections to Yield Compounds **11**, **12α**, and **12β**)

Compound **8** (5.0 mg, 2.8 μmol) was dissolved in 7 N NH_3_ in MeOH (200 μL). The reaction mixture was stirred for 2 days at room temperature and evaporated to dryness. HRMS (ESI-TOF) confirmed the global deprotection and formation of compound **11**. The crude product was dissolved in water and used as such for strain-promoted alkyne-nitrone cycloaddition (SPANC) reactions. Compounds **12α** and **12β** were synthesized following the same procedure described for compound **11**. The applicability of the nitrone-modified glycoclusters **11**, **12α**, and **12β** for SPANC ligation was first tested by labeling the glycoclusters with dibenzylcyclooctyne-PEG4-5/6-carboxyrhodamine dye (compounds **13**, **14α**, and **14β**, Supporting Information). **11**: HRMS (ESI-TOF): *m*/*z* C_55_H_85_N_13_NaO_29_^+^ [M + Na]^+^ requires 1414.55, found 1414.56. **12α**: HRMS (ESI-TOF): *m*/*z* C_73_H_117_N_13_O_44_^2+^ [(M + 2 H)/2]^2+^ requires 939.8653, found 939.8671. **12β**: HRMS (ESI-TOF): *m*/*z* C_73_H_117_N_13_O_44_^2+^ [(M + 2 H)/2]^2+^ requires 939.8653, found 939.8680.

### Synthesis of the AF488-Labeled Glycocluster–Oligonucleotide Conjugates **ON5**–**7**

The DBCO-modified oligonucleotide **ON1** was synthesized on a 1.0 μmol scale using an automatic DNA/RNA synthesizer and commercially available (2-dimethoxytrityloxymethyl-6-fluorenylmethoxycarbonylamino-hexane-1-succinoyl) long-chain alkylamino-CPG solid support. Commercially available building blocks of 2′-OMe nucleoside phosphoramidites and 5′-DBCO-triethyleneglycol phosphoramidite were used for the chain elongation. The oligonucleotide was a full phosphorothioate except for one phosphodiester bond connecting the 5′-DBCO-triethyleneglycol unit to the oligonucleotide. To prevent loss of DBCO in the 5′-DBCO-triethyleneglycol phosphoramidite coupling step, (1*S*)-(+)-(10-camphorsulfonyl)-oxaziridine was used as an oxidizer, as recommended by the manufacturer. The oligonucleotide was released from the support by the usual ammonolysis protocol for DBCO-modified oligonucleotides (concentrated ammonium hydroxide, 2 h at 65 °C), homogenized by RP-HPLC, and lyophilized. The DBCO–oligonucleotide was then dissolved in water and incubated with aqueous solutions of nitrone-modified glycoclusters **11**, **12α**, and **12β** (2 equiv) overnight at room temperature to give glycocluster–oligonucleotide conjugates. The resulting glycocluster–oligonucleotide conjugates **ON2**–**ON4** were homogenized by RP-HPLC (Scheme 3A) and lyophilized to dryness. The isolated yields 17, 11, and 15% of the conjugates **ON2**, **ON3**, and **ON4**, in this order, were determined according to UV absorbance at λ = 260 nm. The authenticity of the products was verified by MS (ESI-TOF) spectroscopy, and they were then exposed to the AF488 labeling, following an established protocol: The glycocluster–oligonucleotide conjugates were dissolved in 0.1 M sodium borate (aq, pH 8.5), AF488 NHS ester (purchased from Lumiprobe, 20 equiv in DMSO) was added, and the reaction mixtures were incubated overnight at room temperature. The resulting AF488-labeled glycocluster–oligonucleotide conjugates were purified by RP-HPLC and lyophilized to dryness to yield **ON5**–**ON7** in ca. 20% yields. The homogeneity of the products was confirmed by RP-HPLC, and the authenticity of the products was verified by MS (orbitrap) spectroscopy (Scheme 3B, Figures S57–S60, and Table S1).

### SNA Synthesis

To a solution of C_60_ Buckminsterfullerene core **15** (60 nmol in 65 μL DMSO), BCN-modified oligonucleotide **ON9** (20 nmol in 10 μL H_2_O) was added. The reaction mixture was gently shaken overnight at room temperature and the monosubstituted product was purified by RP-HPLC (Figure S61, an analytical C-18 column (250 × 4.6 mm, 5 μm), detection at λ = 260 nm, gradient elution (0–20 min) from 40 to 100% MeCN in 50 mM aqueous triethylammonium acetate, flow rate 1.0 mL min^–1^). The product fractions were collected, lyophilized to dryness, and the authenticity of the monosubstituted compound **16** was verified by MS (ESI-TOF, Figure S61). The isolated yield of the product (9.6 nmol, 48%) was determined by UV absorbance at 260 nm. The monosubstituted product **16** (7.7 nmol) was mixed with **ON9** (110 nmol, 1.2 equiv of **ON9**/azide arm) in 1.5 M aqueous NaCl solution, and the reaction mixture was gently shaken for 72 h at room temperature. The resulting **SNA1** was purified by RP-HPLC (Scheme 4A, an analytical C-18 column Phenomenex, Aeris 3.6 μm WIDEPORE XB-C18 200 Å, 150 × 4.6 mm, detection at λ = 260 nm, gradient elution (0–30 min) from 5 to 45% MeCN in 50 mM aqueous triethylammonium acetate, flow rate 1.0 mL min^–1^). The product fractions were collected and lyophilized to dryness. The isolated yield of the product (4.0 nmol, 52%) was determined by UV absorbance at 260 nm. The homogeneity of **SNA1** was confirmed by RP-HPLC, size exclusion chromatography (SEC), and PAGE, and the authenticity of **SNA1** was confirmed by MS (a spectrometer equipped with a hybrid quadrupole orbitrap and nano-ESI ionization; Scheme 4A–D).

### UV Thermal Melting Studies

The thermal melting curves of the hybridized SNA/oligonucleotide complexes were measured at 260 nm with a PerkinElmer Lambda 35 UV–vis spectrometer equipped with a multiple cell holder and a Peltier temperature controller. Additionally, an internal thermometer was used. The temperature was changed between 10 and 80 °C at the rate of 0.5 °C min^–1^. Each *T*_m_ value was determined from the maximum of the first derivative of the melting curve (average of three heating and three cooling curves). The measurements were performed using 0.083 μmol L^–1^ SNA and 12 equiv (1.0 μmol L^–1^) of the oligonucleotide in 10 mmol L^–1^ sodium cacodylate buffer (pH 7.0) with 0.1 mol L^–1^ NaCl.

### PAGE Analysis of SNAs

Native 6% Tris base, boric acid, ethylenediaminetetraacetic acid (EDTA), and acrylamide (TBE) gel were used for confirming the purity of **SNA1** and the hybridization of the glycocluster-modified oligonucleotides **ON5**–**7** with the complementary SNA. A pre-cast gel cover (10 cm × 10 cm in size, Thermo Fisher Scientific) was fixed into a vertical electrophoresis chamber, and the running buffer (90 mM Tris, 90 mM borate, and 2 mM EDTA, 8.3 pH) was filled into the chamber. SNA samples were prepared by mixing 5 μL of 0.05 μM **SNA1** in phosphate-buffered saline (PBS), pH 7.4, with 5 μL TBE sample buffer. Hybridized SNA/oligonucleotide samples were prepared by adding 12 equiv of oligonucleotides **ON5**–**8** in the mixture. The SNA samples and a DNA ladder (100 bp, note: the ladder is just used to confirm the quality and comparability of the runs, and it cannot be used for size evaluation of the SNAs) were loaded and electrophoresed at 200 V constant (45 mA) for approximately 30 min. After completion of electrophoresis, gel was removed from the chamber and the SNA bands were monitored after staining by SYBRTM Gold Nucleic Acid Stain (Thermo Fisher Scientific).

### DLS Experiments

The size of the SNAs was measured at room temperature using a Zetasizer Nano ZS90 (Malvern Instruments Ltd., U.K.). Settings and conditions for the measurements were: material Protein (RI: 1.450; Absorption: 0.001), dispersant water (Viscosity: 0,8872 cP; RI: 1.330) temperature at 20 °C, and equilibration time was 60 s. Each sample (10 μg SNA in 100 μL aqueous 10 mmol L^–1^ PBS, 1.1 mmol L^–1^ M KCl, 0.154 mol L^–1^ NaCl, pH 7.4) was measured three times.

### Spectroscopic Measurements

The binding constants were measured by stepwise addition of **ON5**–**8** to **SNA1** solution starting with an ON:SNA ratio of 2:1 and finishing at a 14:1 ratio. DPBS was used as the solvent. The fluorescence and excitation spectra were recorded with an FLS-1000 spectrofluorometer (Edinburgh Instruments, U.K.). The fluorescence spectra were corrected according to the wavelength sensitivity of the detector and the excitation source intensity.

Time-resolved fluorescence was measured using a time-correlated single photon counting (TCSPC) system (Pico-Quant GmBH, Chaussee, Germany) consisting of a PicoHarp 300 controller and a PDL 800-B driver. The samples were excited with the pulsed diode laser head LDH-P-C-485 at 483 nm at a time resolution of 130 ps. The signals were detected with a microchannel plate photomultiplier tube (Hamamatsu R2809U). The influence of the scattered excitation light was reduced with a cutoff filter (transmission > 490 nm) in front of the monitoring monochromator. Fluorescence decays were collected with a constant accumulation time in the 500–570 nm wavelength range with steps of 10–20 nm. The instrumental response function (IRF) was measured separately, and the decays were deconvoluted and fitted globally by applying the iterative least-squares method to the sum of 2 exponents (eq 1).
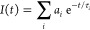
1In this eq 1, τ*_i_* is the global lifetime and *a_i_* is the local amplitude (preexponential factor). The average lifetimes ⟨τ⟩ were calculated using eq 2
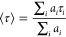
2

### Materials for Cell Uptake Studies

All cell reagents were purchased via CityLab Helsinki/Turku/Finland. RPMI 1640 Medium (Gibco, catalog no: 21875034). 10× DPBS (DPBS, 10×, no calcium, no magnesium, catalog number 1400-067) was diluted to autoclaved Milli-Q water to make 1× DPBS (osmolality in 10×: 2630–3000 mOsm kg^–1^, after dilution osmolality in 1×: 263–300 mOsm kg^–1^). TrypLE Express enzyme 1× without phenol red (Gibco, catalog number 12604021). PBS (Lonza, catalog number:BE17-516F) 6.7 mM (PO_4_), 50 mM, pH 7.4 without calcium, magnesium, and phenol red. Live dead kit (Invitrogen LIVE/DEADT Viability/Cytotoxicity Kit, for mammalian cells catalog number: L3224). Paraformaldehyde (4%) was obtained from the University of Helsinki. Fetal bovine serum (FBS) (Gibco, catalog no: 10270-106). Antibiotic, penicillin–streptomycin (Gibco, catalog no: 15140-122). DAPI + mountant [Prolong Diamond antifade mountant with DAPI (Invitrogen by Thermo Fisher Scientific)]; 8-well chamber slides [Nunc Lab-Tek 2 Chamber slide, 8-well (Thermo Scientific)].

### Cell Culture

PC3 cells were purchased from American Tissue Culture Collection (ATCC) and grown in RPMI 1640 medium (Invitrogen) supplemented with 10% fetal bovine serum (FBS), and 1% penicillin–streptomycin. During subculturing, the cells were washed with DPBS and detached from the T25 flask using TrypLe Express. After aliquoting the cells to new flasks (in 1:3–1:6 ratio, v/v), the cell culture was maintained at 37 °C with 5% CO_2_. For upcoming cell uptake experiments, the cells were plated in eight-well chamber slides at 30% confluency 24 h prior to the treatment. Cell passage numbers during the assays in question ranged from p46 to p53. During subculturing, the cells were examined through a Nikon TMS inverted microscope using 10× Ph1 objective.

### Cell Uptake Experiments

The PC3 cell uptake studies of the oligonucleotides and SNAs were compared to untreated PC3 cells as control. For the experiments, the cells were seeded at 30% confluency in an eight-well chamber slide [Nunc Lab-Tek 2 Chamber slide, 8-well (Thermo Scientific)] 24 h before experiments. With oligonucleotides and SNAs, the used cell confluency was about 90%. The cell uptake studies were carried out at the concentration of 120 nM of oligonucleotides and 10 nM SNA. After that, the cells were incubated (37 °C with 5% CO_2_) with the studied compounds or in PBS (controls) for 4 h. After uptake, the cells were washed three times with PBS, each wash taking 5 min. The cells were then fixed using 4% paraformaldehyde solution (diluted in PBS). The intracellular delivery of oligonucleotides and SNAs was quantified via wide-field microscopy on a Nikon Eclipse Ti2-E microscope using the Alexa 488 (475 nm) and DAPI (395 nm) channels and normalized based on the intensity of the untreated control cells.

### Confocal and Wide-Field Microscopy

PC3 cells were cultured on the eight-well chamber slides and incubated for 24 h before being treated with the appropriate oligonucleotide formulation (final oligonucleotide concentration = 120 nM). The treated cells were then fixed using 4% perfluoroalkoxy alkanes (PFA) and washed three times with PBS. The well was removed, and the nuclei of the cells were stained and mounted on the slides using Prolong Diamond antifade mountant with DAPI. Imaging of the cells was performed using a Nikon Eclipse Ti2-E wide-field inverted microscope using Nikon NIS Elements AR 5.11.01 64-bit acquisition software. The objective, used as dry, was a 20× Nikon CFI Plan Apo Lambda 0,75 NA with a 1 mm working distance. Excitation wavelengths were for DAPI channel: 395/25 nm and emission wavelength: 435/26 nm (DAPI sPx), and for Alexa 488-channel: 475/28 nm and emission wavelength: 515/30 nm (GFP sPx). Images were acquired using a Hamamatsu ORCA-Flash 4.0 v3 sCMOS camera with a pixel size of 6.5 μm. Images were 12-bit with pixel dimensions 600 × 600 px. Wide-field microscopy was carried out with a Nikon Eclipse Ti2-E wide-field microscope. Imaging was performed with Nikon NIS Elements 4.11 acquisition software, and the picture format of the images is 2048 × 2044 px. The picture format of cropped images is 600 × 600 px. The images were edited with the Fiji-ImageJ software (2.1.0) by adjusting minimum and maximum, brightness and contrast, sharpening, adding scale bar, and adding text bar. During subculturing, the cells were examined through a Nikon TMS inverted microscope with a 10× Ph1 objective.

## Supporting Information Available

NMR data of the carbohydrates synthesized, experimental details for small-molecule trials of the SPANC conjugation, MS and HPLC data of the oligonucleotide conjugates, additional fluorescence data, and cell uptake images (PDF)
