# Supplementary material for: Assembly of Bleomycin Saccharide-Decorated Spherical Nucleic Acids
Source: Bioconjug Chem. 2022 Jan 5;33(1):206–18. doi: 10.1021/acs.bioconjchem.1c00539 (PMC8778632; doi:10.1021/acs.bioconjchem.1c00539)
Supplement: Supplementary file 1 — bc1c00539_si_001.pdf [file bc1c00539_si_001.pdf]

Supporting Information to:

Assembly of Bleomycin Saccharide Decorated Spherical Nucleic Acids

Ville Tähtinen,*[a] Vijay Gulumkar,[a] Sajal K. Maity,[a] Ann-Mari Yliperttula,[a,b] Saara Siekkinen,[a,b] Toni Laine[a], Ekaterina Lisitsyna,[c] Iida Haapalehto[c], Tapani Viitala[b,d], Elina Vuorimaa-Laukkanen,[c] Marjo Yliperttula[b] and Pasi Virta*[a]

[a]Department of Chemistry, University of Turku, FI-20500 Turku, Finland

[b]Division of Pharmaceutical Biosciences, Faculty of Pharmacy, University of Helsinki, FI-00014 Helsinki, Finland

[c]Faculty of Engineering and Natural Sciences, Tampere University, FI-33014 Tampere, Finland

[d] Pharmaceutical Sciences, Faculty of Science and Engineering, Åbo Akademi University, 20520 Turku, Finland

Email: pamavi@utu.fi (the corresponding author)

Contents

Figure S1. ^{1}H NMR (500 MHz, $CDCl_3$) spectrum of compound **2α**. 4

Figure S2. ^{1}H NMR (500 MHz, $CDCl_3$) spectrum of compound **2α**. 4

Figure S3. ^{1}H NMR (500 MHz, $CDCl_3$) spectrum of compound **2α**. 5

Figure S4. ^{1}H NMR (500 MHz, $CDCl_3$) spectrum of compound **2α**. 5

Figure S5. ^{13}C NMR (125 MHz, $CDCl_3$) spectrum of compound **2α**. 6

Figure S6. ^{13}C NMR (125 MHz, $CDCl_3$) spectrum of compound **2α**. 6

Figure S7. ^{13}C NMR (125 MHz, $CDCl_3$) spectrum of compound **2α**. 7

Figure S8. ^{1}H NMR (500 MHz, $CDCl_3$) spectrum of compound **2β**. 7

Figure S9. ^{1}H NMR (500 MHz, $CDCl_3$) spectrum of compound **2β**. 8

Figure S10. ^{1}H NMR (500 MHz, $CDCl_3$) spectrum of compound **2β**. 8

Figure S11. ^{1}H NMR (500 MHz, $CDCl_3$) spectrum of compound **2β**. 9

Figure S12. ^{13}C NMR (125 MHz, $CDCl_3$) spectrum of compound **2β**. 9

Figure S13. ^{13}C NMR (125 MHz, $CDCl_3$) spectrum of compound **2β**. 10

Figure S14. ^{13}C NMR (125 MHz, $CDCl_3$) spectrum of compound **2β**. 10

Figure S15. ^{1}H NMR (500 MHz, $CDCl_3$) spectrum of compound **3α**. 11

Figure S16. ^{1}H NMR (500 MHz, $CDCl_3$) spectrum of compound **3α**. 11

Figure S17. ^{1}H NMR (500 MHz, $CDCl_3$) spectrum of compound **3α**. 12

Figure S18. ^{1}H NMR (500 MHz, $CDCl_3$) spectrum of compound **3α**. 12

Figure S19. ^{13}C NMR (125 MHz, $CDCl_3$) spectrum of compound **3α**. 13
Figure S20. ^{13}C NMR (125 MHz, $CDCl_3$) spectrum of compound **3α**. 13
Figure S21. ^{13}C NMR (125 MHz, $CDCl_3$) spectrum of compound **3α**. 14
Figure S22. ^{1}H NMR (500 MHz, $CDCl_3$) spectrum of compound **3β**. 14
Figure S23. ^{1}H NMR (500 MHz, $CDCl_3$) spectrum of compound **3β**. 15
Figure S24. ^{1}H NMR (500 MHz, $CDCl_3$) spectrum of compound **3β**. 15
Figure S25. ^{1}H NMR (500 MHz, $CDCl_3$) spectrum of compound **3β**. 16
Figure S26. ^{13}C NMR (125 MHz, $CDCl_3$) spectrum of compound **3β**. 16
Figure S27. ^{13}C NMR (125 MHz, $CDCl_3$) spectrum of compound **3β**. 17
Figure S28. ^{13}C NMR (125 MHz, $CDCl_3$) spectrum of compound **3β**. 17
Figure S29. ^{1}H NMR (500 MHz, $CDCl_3$) spectrum of compound **5**. 18
Figure S30. ^{1}H NMR (500 MHz, $CDCl_3$) spectrum of compound **5**. 18
Figure S31. ^{1}H NMR (500 MHz, $CDCl_3$) spectrum of compound **5**. 19
Figure S32. ^{13}C NMR (125 MHz, $CDCl_3$) spectrum of compound **5**. 19
Figure S33. ^{13}C NMR (125 MHz, $CDCl_3$) spectrum of compound **5**. 20
Figure S34. ^{1}H NMR (500 MHz, CD3OD) spectrum of compound **8**....................20
Figure S35. ^{1}H NMR (500 MHz, CD_3OD) spectrum of compound **8**. 21
Figure S36. ^{13}C NMR (125 MHz, CD_3OD) spectrum of compound **8**. 21
Figure S37. ^{13}C NMR (125 MHz, CD_3OD) spectrum of compound **8**. 22
Figure S38. ^{1}H NMR (500 MHz, CD_3OD) spectrum of compound **10α**. 22
Figure S39. ^{1}H NMR (500 MHz, CD_3OD) spectrum of compound **10α**. 23
Figure S40. ^{1}H NMR (500 MHz, CD_3OD) spectrum of compound **10α**. 23
Figure S41. ^{1}H NMR (500 MHz, CD_3OD) spectrum of compound **10α**. 24
Figure S42. ^{1}H NMR (500 MHz, CD_3OD) spectrum of compound **10α**. 24
Figure S43. ^{13}C NMR (125 MHz, CD_3OD) spectrum of compound **10α**. 25
Figure S44. ^{13}C NMR (125 MHz, CD_3OD) spectrum of compound **10α**. 26
Figure S45. ^{13}C NMR (125 MHz, CD_3OD) spectrum of compound **10α**. 26
Figure S46. ^{13}C NMR (125 MHz, CD_3OD) spectrum of compound **10α**. 27
Figure S47. ^{13}C NMR (125 MHz, CD_3OD) spectrum of compound **10α**. 27
Figure S48. ^{1}H NMR (500 MHz, CD_3OD) spectrum of compound **10β**. 28
Figure S49. ^{1}H NMR (500 MHz, CD_3OD) spectrum of compound **10β**. 28
Figure S50. ^{1}H NMR (500 MHz, CD_3OD) spectrum of compound **10β**. 29
Figure S51. ^{1}H NMR (500 MHz, CD_3OD) spectrum of compound **10β**. 29
Figure S52. ^{13}C NMR (125 MHz, CD_3OD) spectrum of compound **10β**. 30

Figure S53. ^{13}C NMR (125 MHz, CD_3OD) spectrum of compound **10β**. 30
Figure S54. ^{13}C NMR (125 MHz, CD_3OD) spectrum of compound **10β**. 31
Figure S55. ^{13}C NMR (125 MHz, CD_3OD) spectrum of compound **10β**. 31
Figure S56. ^{13}C NMR (125 MHz, CD_3OD) spectrum of compound **10β**. 32
SPANC Labeling of Nitrone-Modified Glycoclusters 11, 12α and 12β with Rhodamine Dye (compounds 13, 14α and 14β)..32
Scheme S1. SPANC labeling of nitrone-modified glycoclusters with a rhodamine dye.......................33
Figure S57. A) RP HPLC profile and B) MS (ESI-TOF) spectrum of **ON8**. 34
Figure S58. A) RP HPLC profile and B) MS (orbitrap) spectrum of **ON5**.. 35
Figure S59. A) RP HPLC profile and B) MS (orbitrap) spectrum of **ON6**. 36
Figure S60. A) RP HPLC profile and B) MS (orbitrap) spectrum of **ON7**. 37
Table S1. MS data of AF488-labeled oligoribonucleotides. 37
Figure S61. A) Crude RP HPLC profile of the monosubstituted SNA (compound **16**). B) MS (ESI-TOF) spectrum of homogenized monosubstituted SNA. 38
Table S2. Fluorescence properties of **ON5–ON8** in the absence and presence of **SNA1**: maximum wavelengths from excitation ($\lambda_{max,ex}$) and fluorescence ($\lambda_{max,FL}$) spectra, lifetime of the short-living component (τ_1), long-living component (τ_2) and the average fluorescence lifetime ($\langle\tau\rangle$). 38
Figure S62. Fluorescence (solid lines) and excitation (dashed lines) spectra of **ON6/SNA1** complexes. 39
Figure S63. Area under the fluorescence spectra for **ON5-8/SNA1** complexes as a function of ON concentration. 39
Figure S64. Fluorescence decays for **ON8** and **ON5** in the absence and presence of **SNA1**. 40
Figure S65. Plots of the average fluorescence lifetime $\langle\tau\rangle$ of **ON5-8:SNA1** complexes as a function of a) ON concentration and b) inverse ON concentration. 41
Figure S66. Uptake of AF488-labeled carbamoyl mannose conjugate **ON5** and **SNA2** by PC3 cells. ..42
Figure S67. Uptake of AF488-labeled α-bleomycin disaccharide conjugate **ON6** and **SNA3** by PC3 cells. 42
Figure S68. Uptake of AF488-labeled β-bleomycin disaccharide conjugate **ON7** and **SNA4** by PC3 cells. 43
Figure S69. Uptake of non-conjugated reference oligonucleotide **ON8** and **SNA5** by PC3 cells. 43
References..44

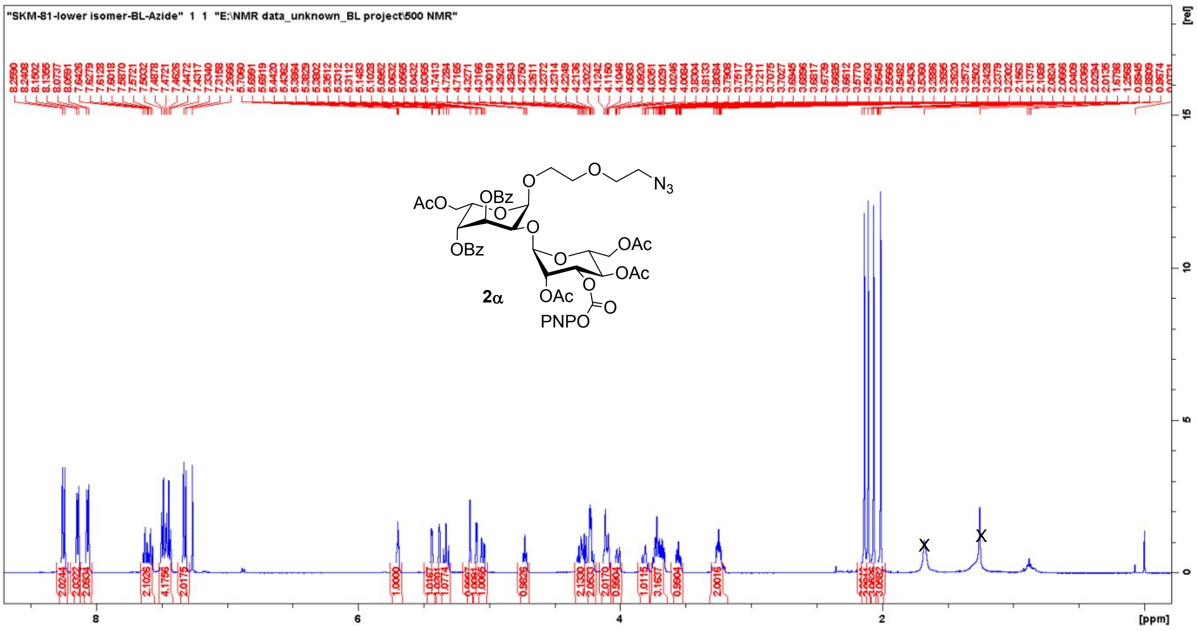

Figure S1. ^{1}H NMR (500 MHz, $CDCl_3$) spectrum of compound **2α**.

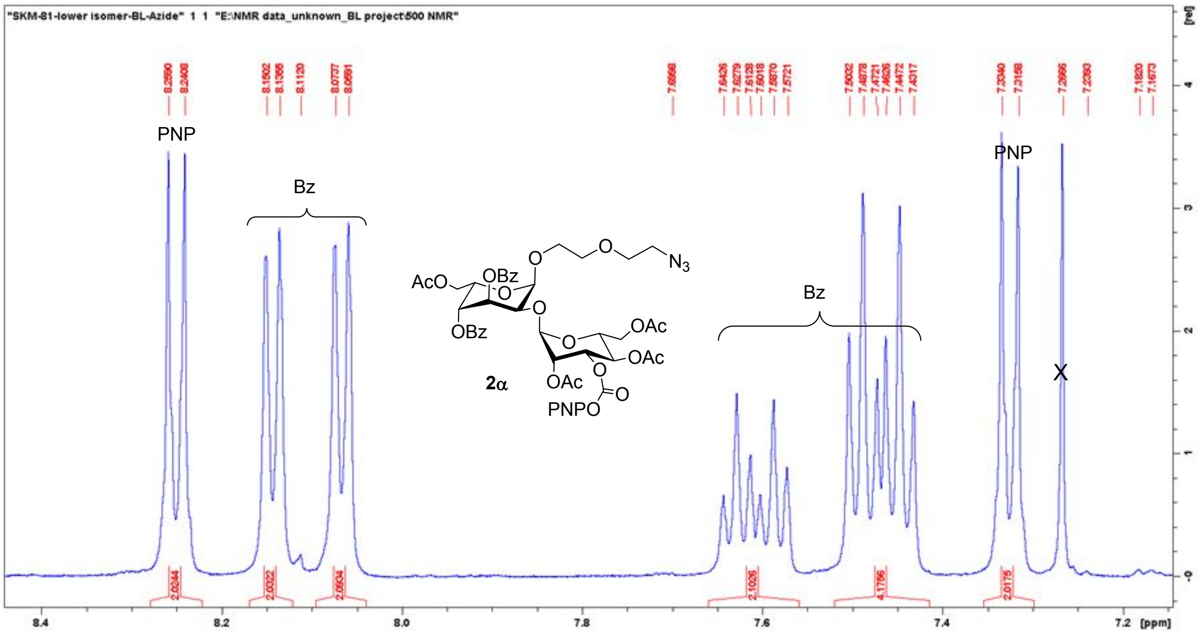

Figure S2. ^{1}H NMR (500 MHz, $CDCl_3$) spectrum of compound **2α**.

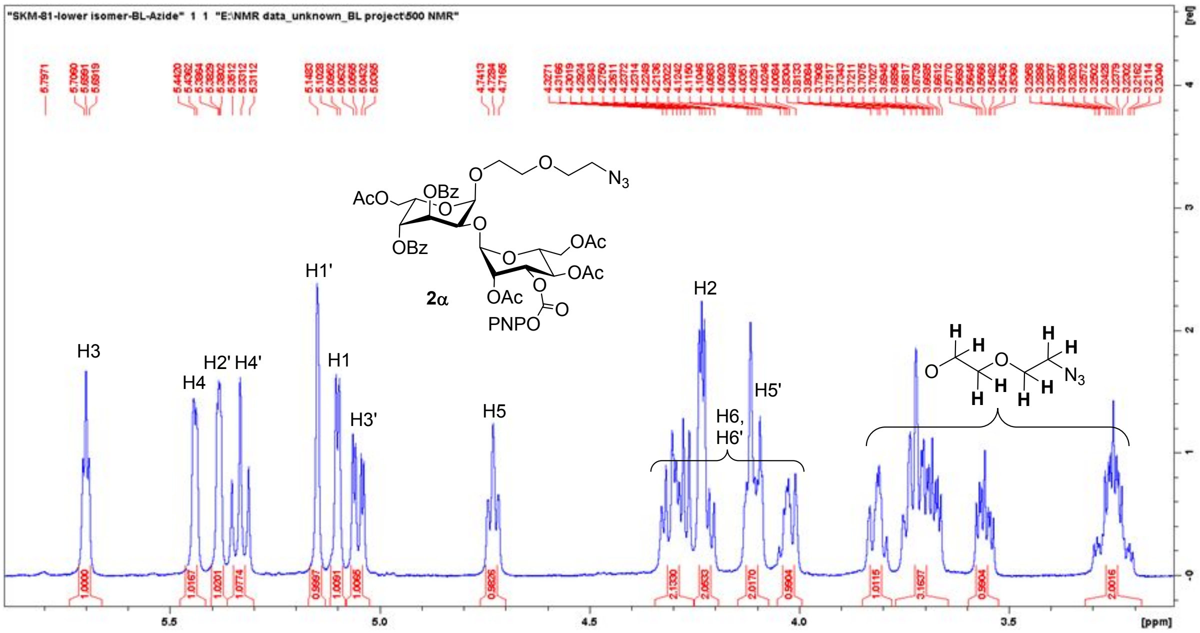

Figure S3. ^{1}H NMR (500 MHz, $CDCl_3$) spectrum of compound **2α**.

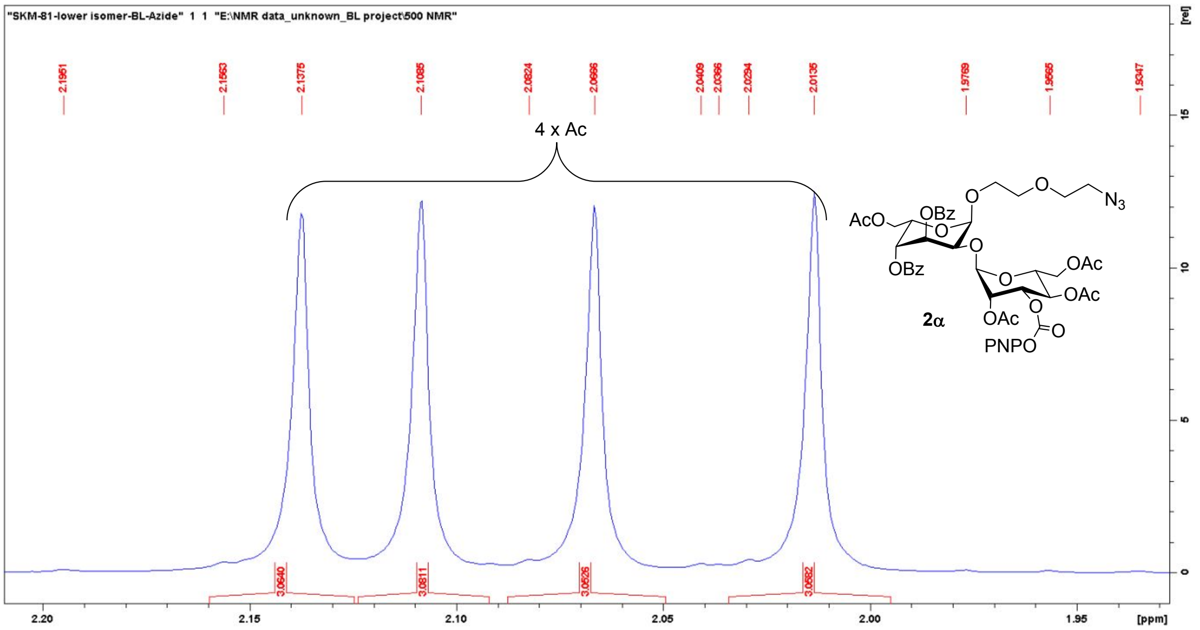

Figure S4. ^{1}H NMR (500 MHz, $CDCl_3$) spectrum of compound **2α**.

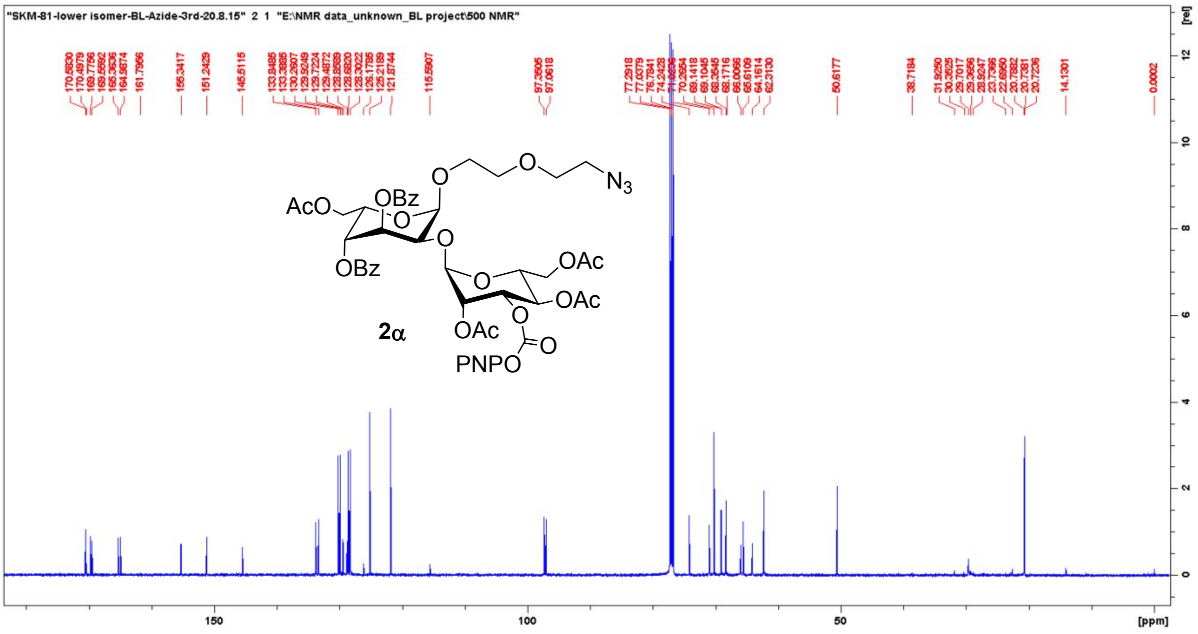

Figure S5. ^{13}C NMR (125 MHz, $CDCl_3$) spectrum of compound **2α**.

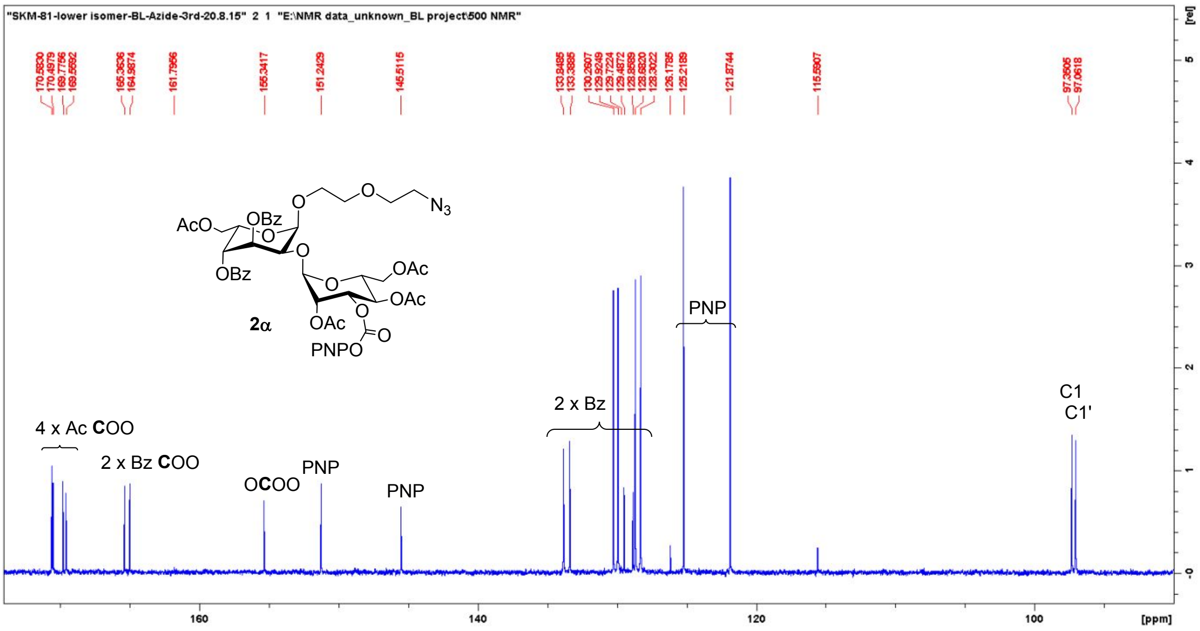

Figure S6. ^{13}C NMR (125 MHz, $CDCl_3$) spectrum of compound **2α**.

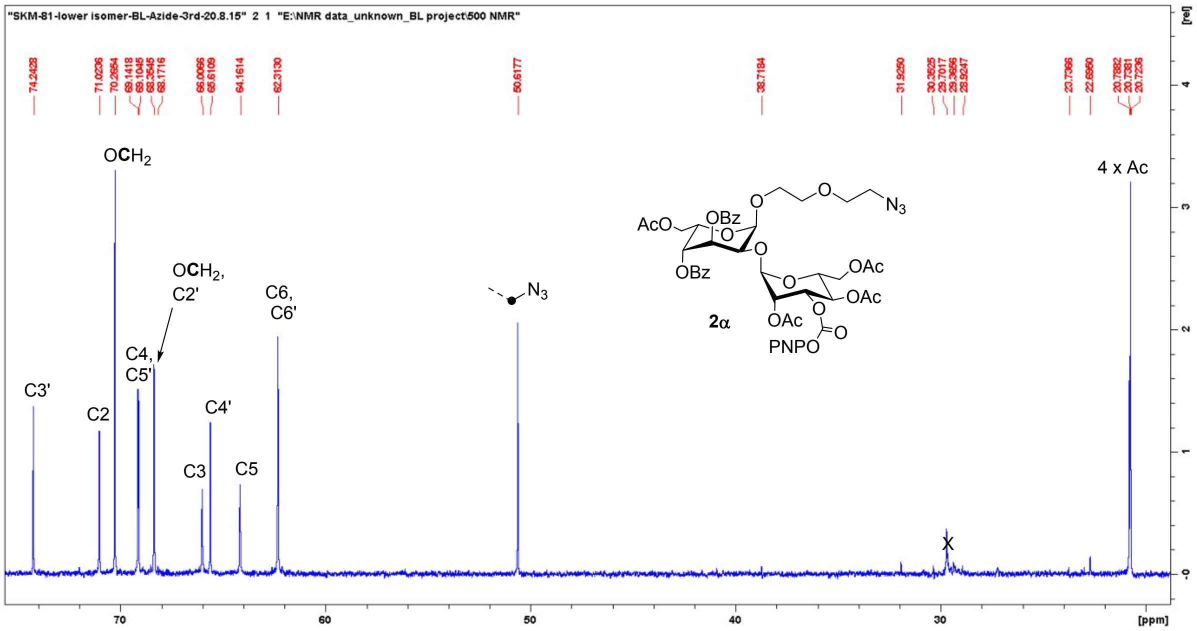

Figure S7. ^{13}C NMR (125 MHz, $CDCl_3$) spectrum of compound **2α**.

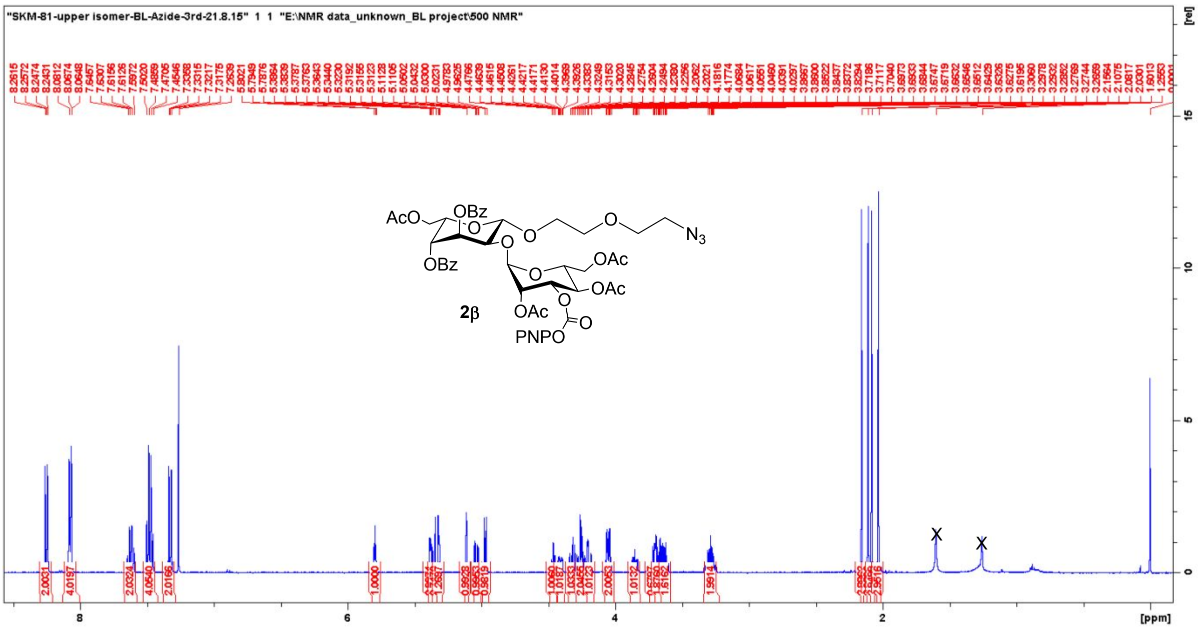

Figure S8. ^{1}H NMR (500 MHz, $CDCl_3$) spectrum of compound **2β**.

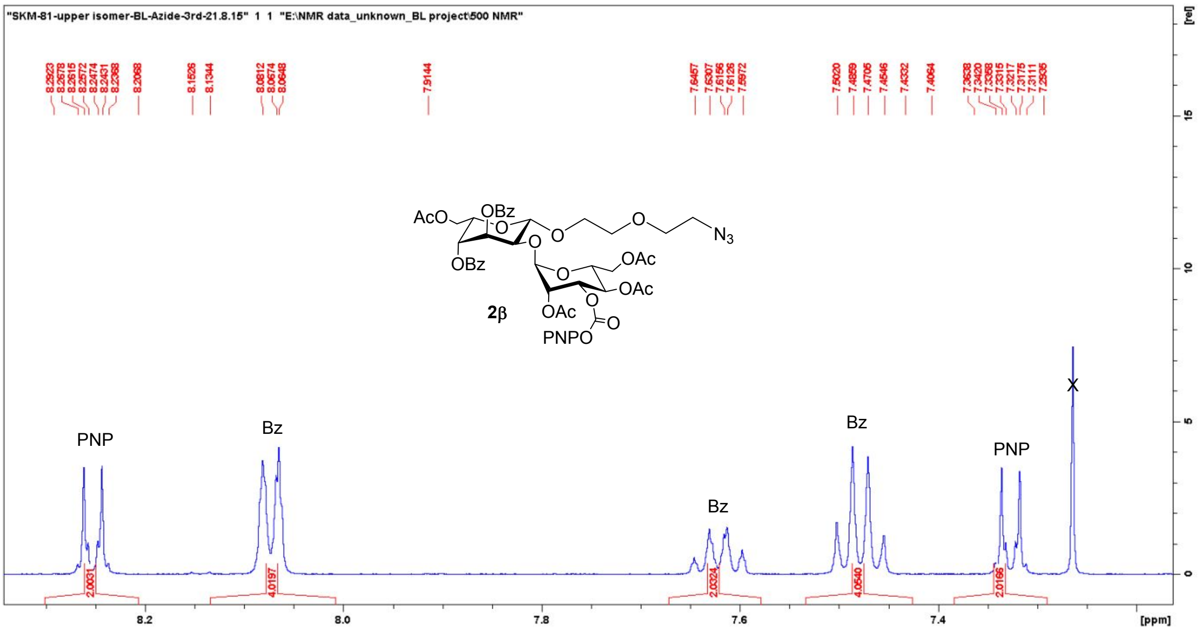

Figure S9. ^{1}H NMR (500 MHz, $CDCl_3$) spectrum of compound **2β**.

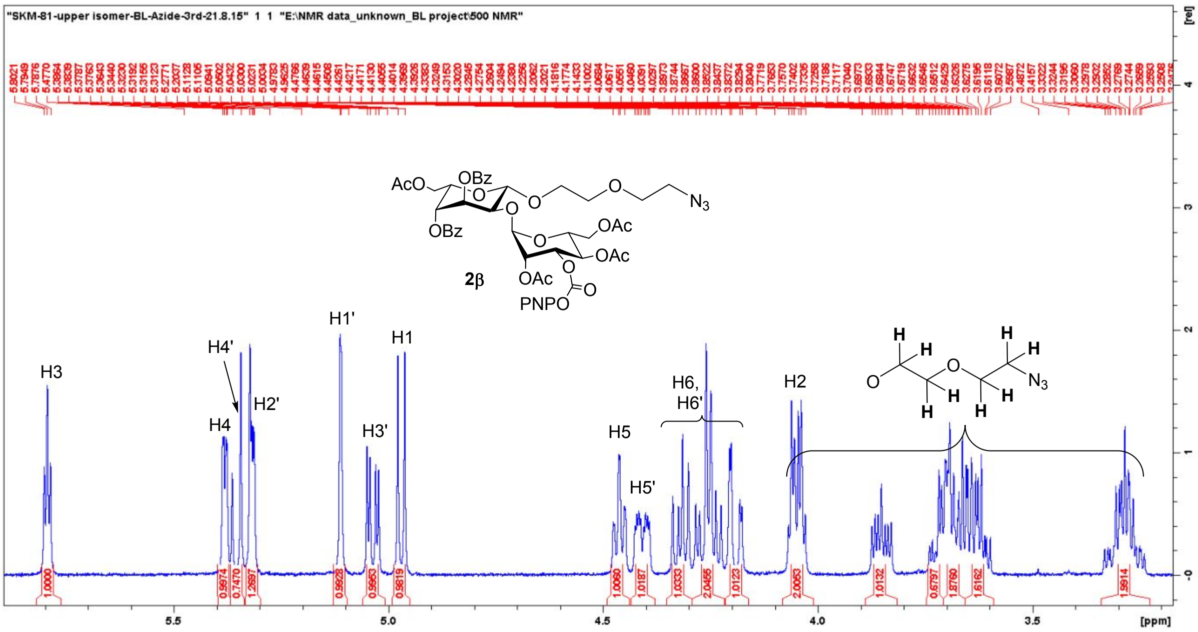

Figure S10. ^{1}H NMR (500 MHz, $CDCl_3$) spectrum of compound **2β**.

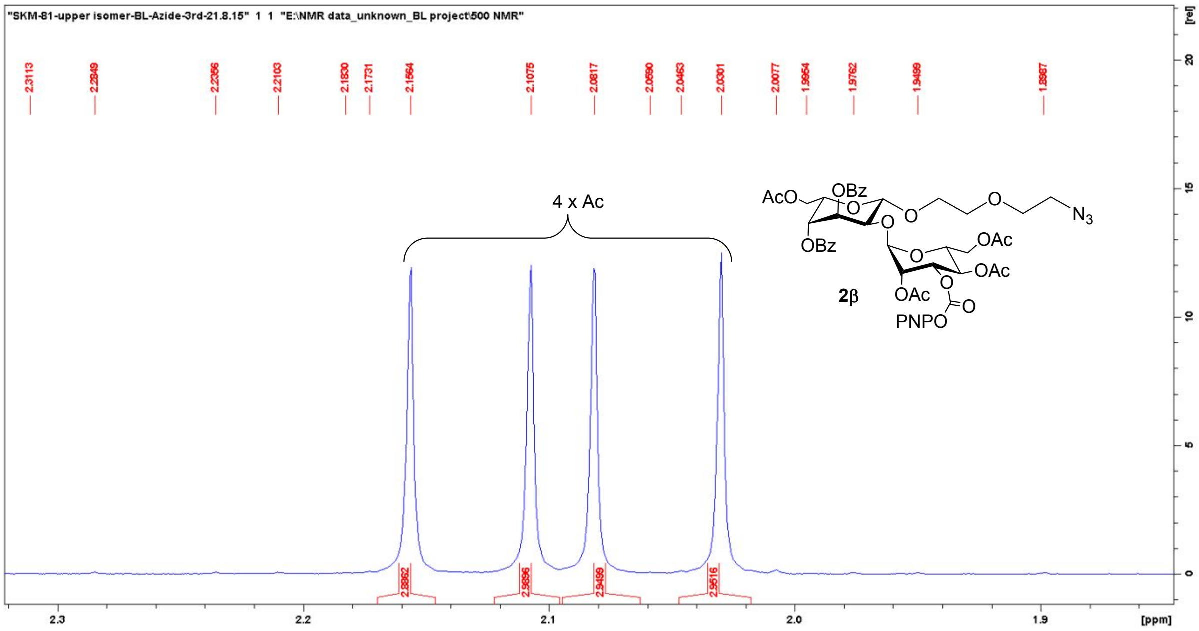

Figure S11. ^{1}H NMR (500 MHz, $CDCl_3$) spectrum of compound **2β**.

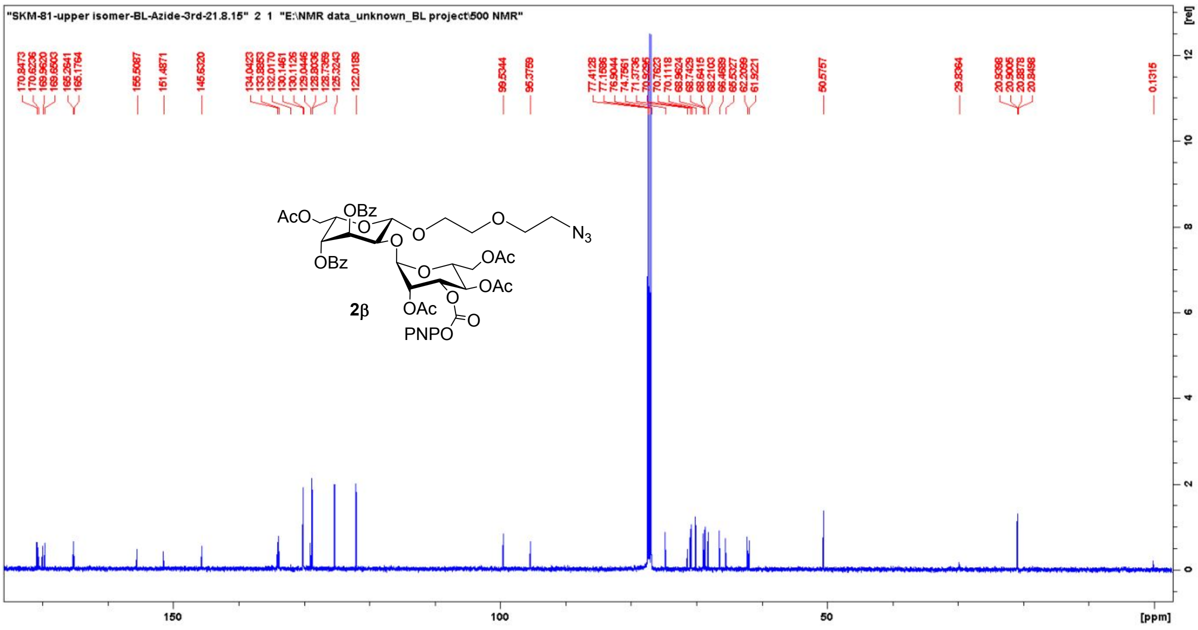

Figure S12. ^{13}C NMR (125 MHz, $CDCl_3$) spectrum of compound **2β**.

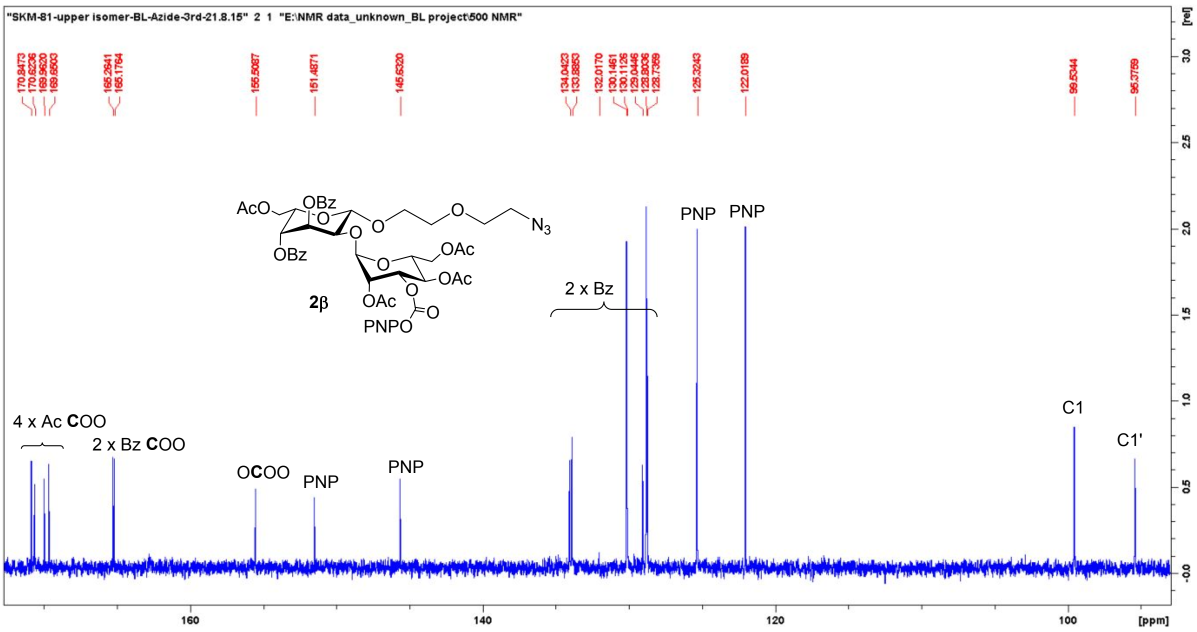

Figure S13. ^{13}C NMR (125 MHz, $CDCl_3$) spectrum of compound **2β**.

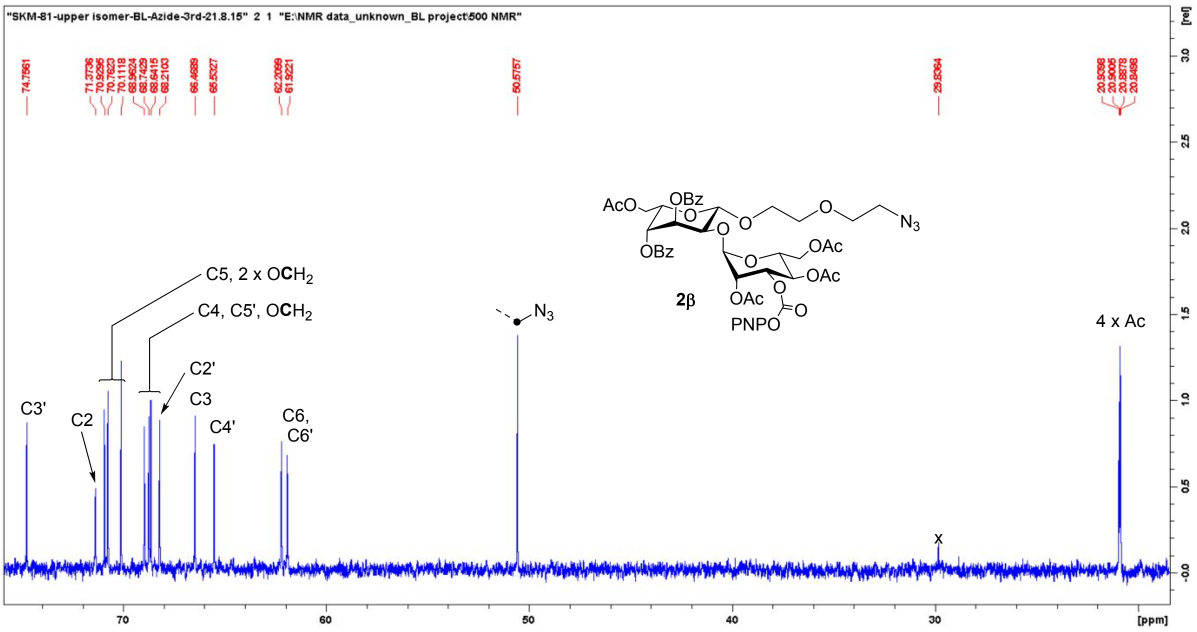

Figure S14. ^{13}C NMR (125 MHz, $CDCl_3$) spectrum of compound **2β**.

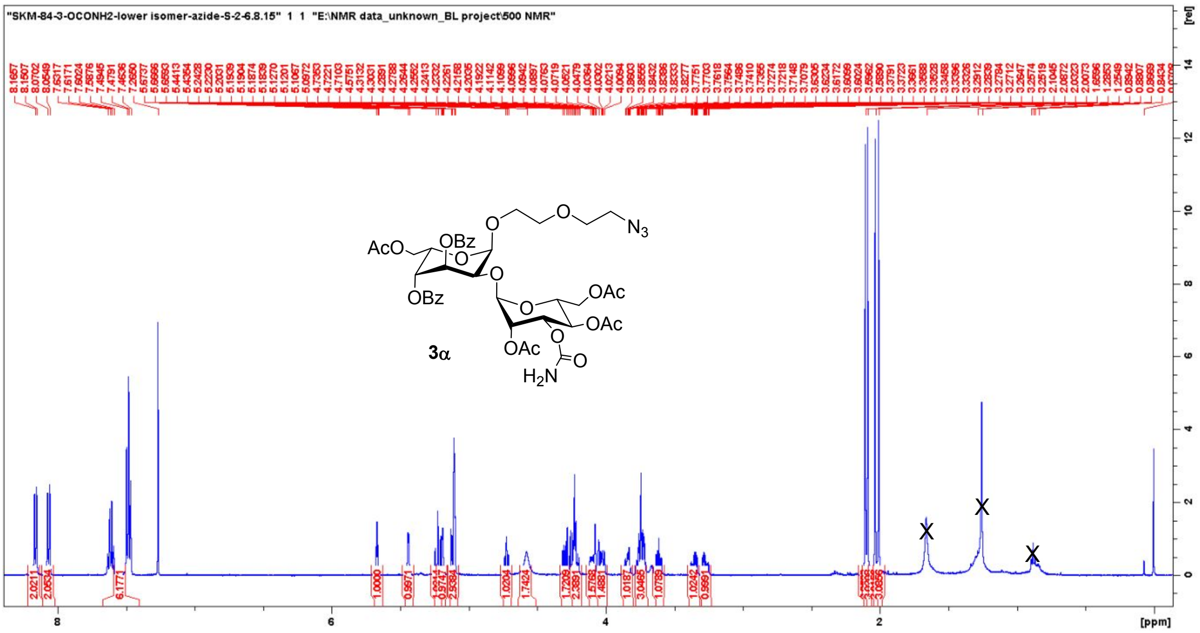

Figure S15. ^{1}H NMR (500 MHz, $CDCl_3$) spectrum of compound **3α**.

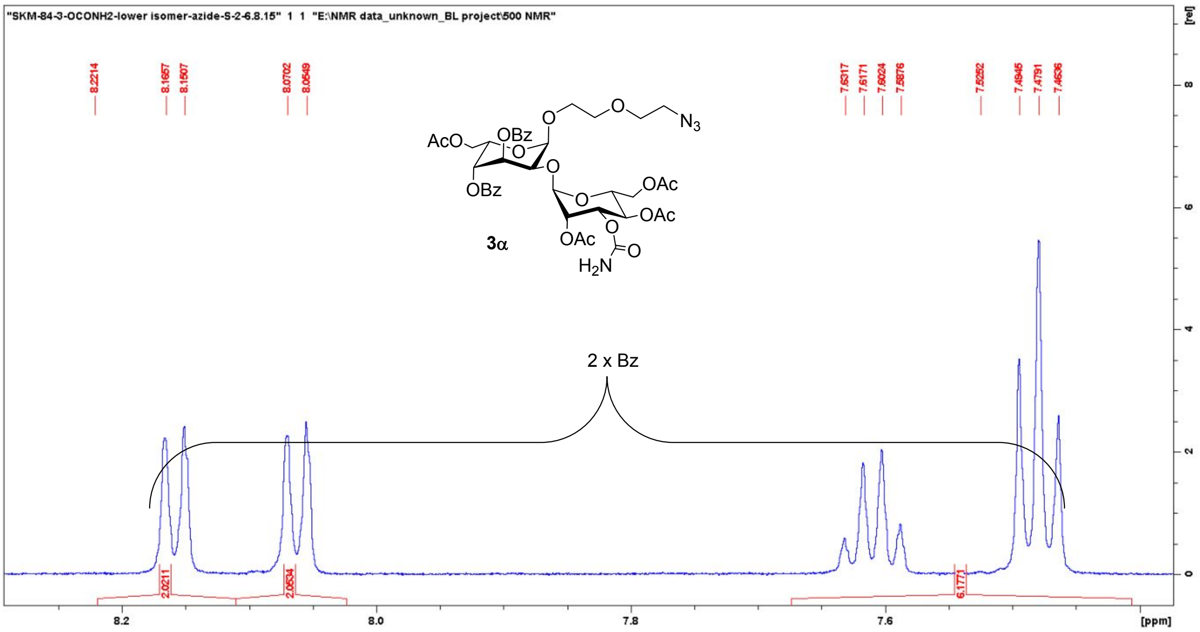

Figure S16. ^{1}H NMR (500 MHz, $CDCl_3$) spectrum of compound **3α**.

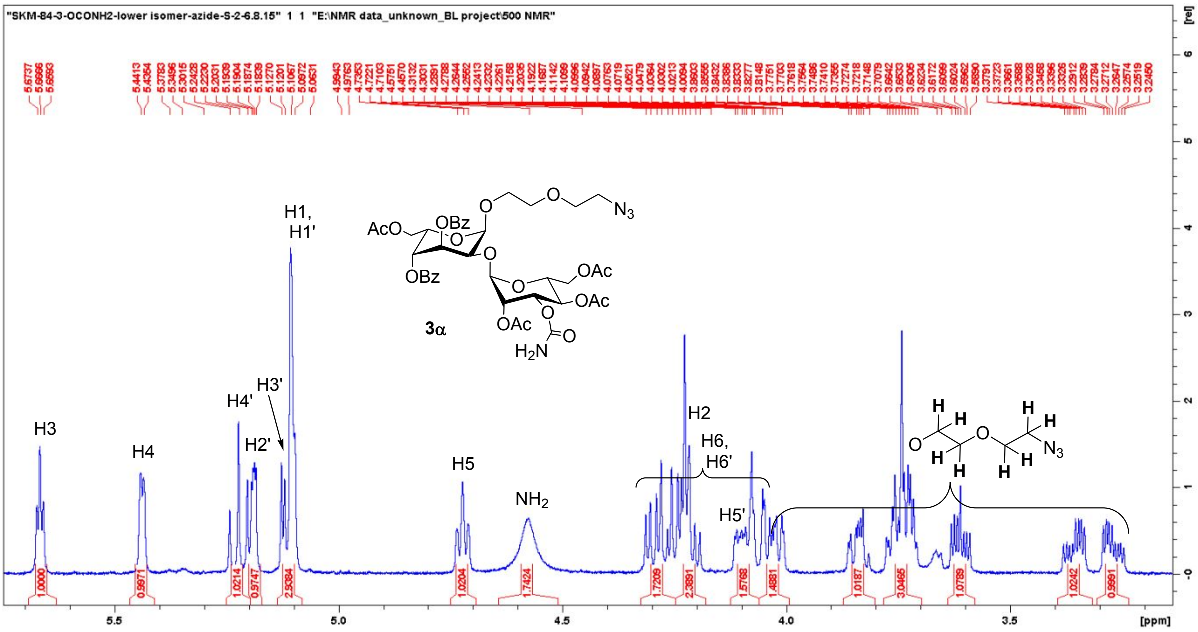

Figure S17. ^{1}H NMR (500 MHz, $CDCl_3$) spectrum of compound **3α**.

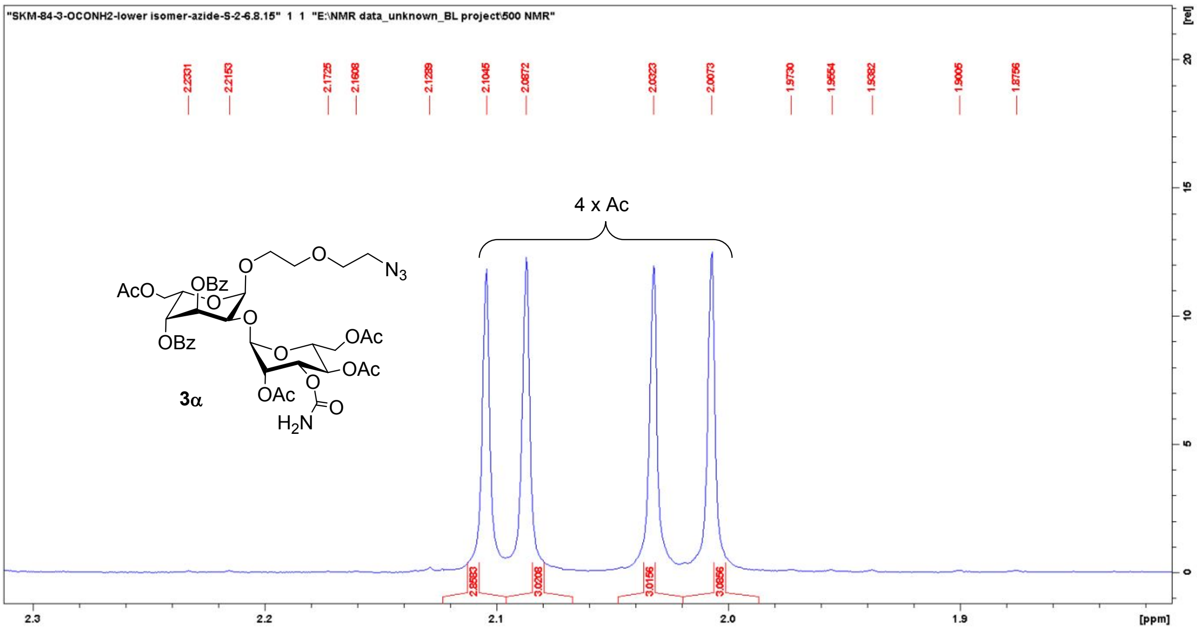

Figure S18. ^{1}H NMR (500 MHz, $CDCl_3$) spectrum of compound **3α**.

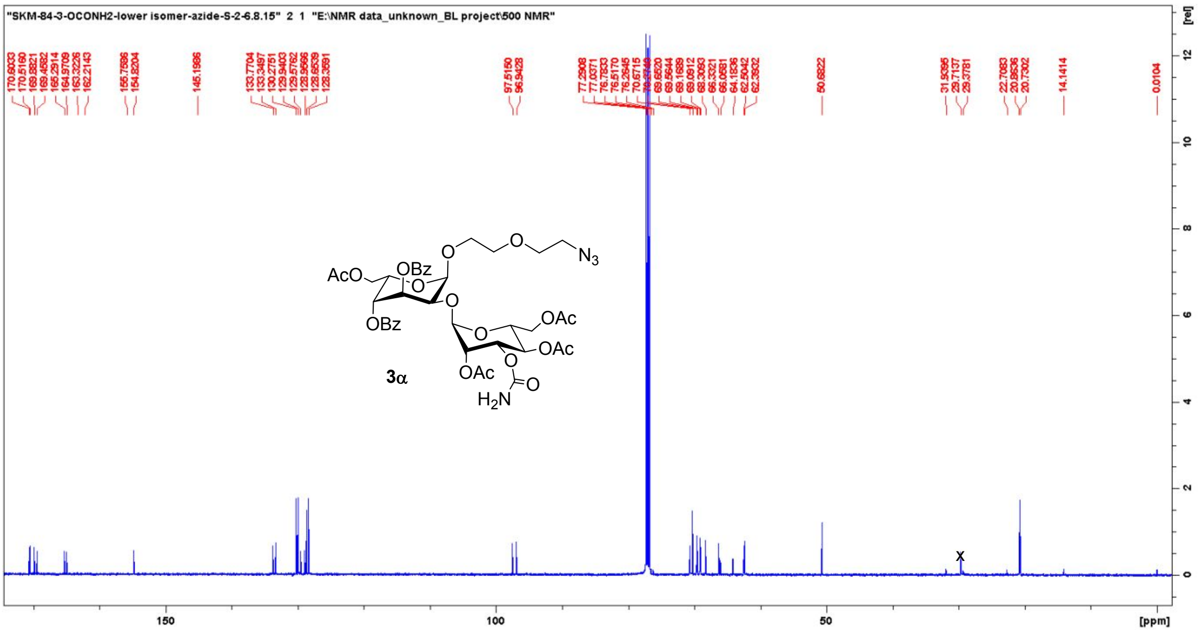

Figure S19. ^{13}C NMR (125 MHz, $CDCl_3$) spectrum of compound **3α**.

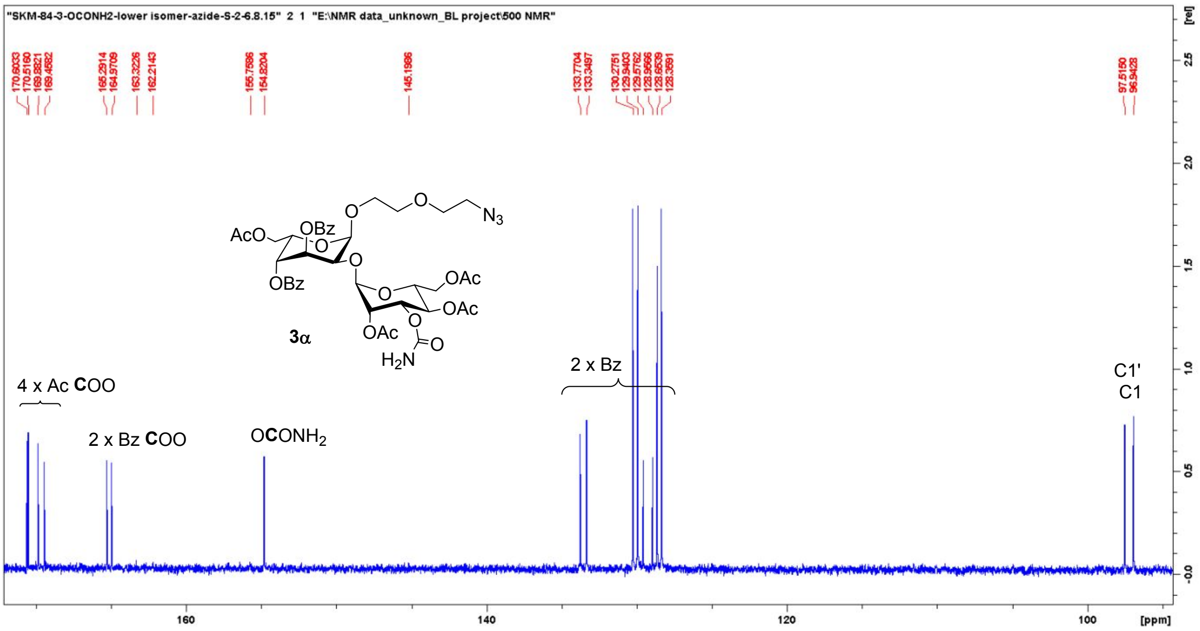

Figure S20. ^{13}C NMR (125 MHz, $CDCl_3$) spectrum of compound **3α**.

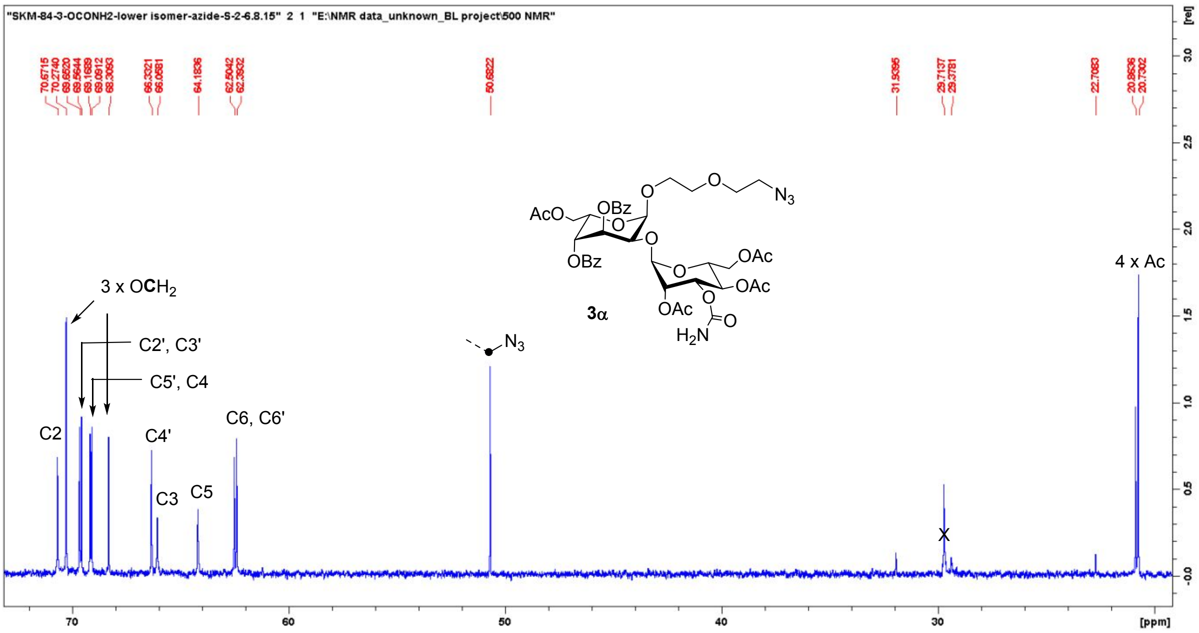

Figure S21. ^{13}C NMR (125 MHz, $CDCl_3$) spectrum of compound **3α**.

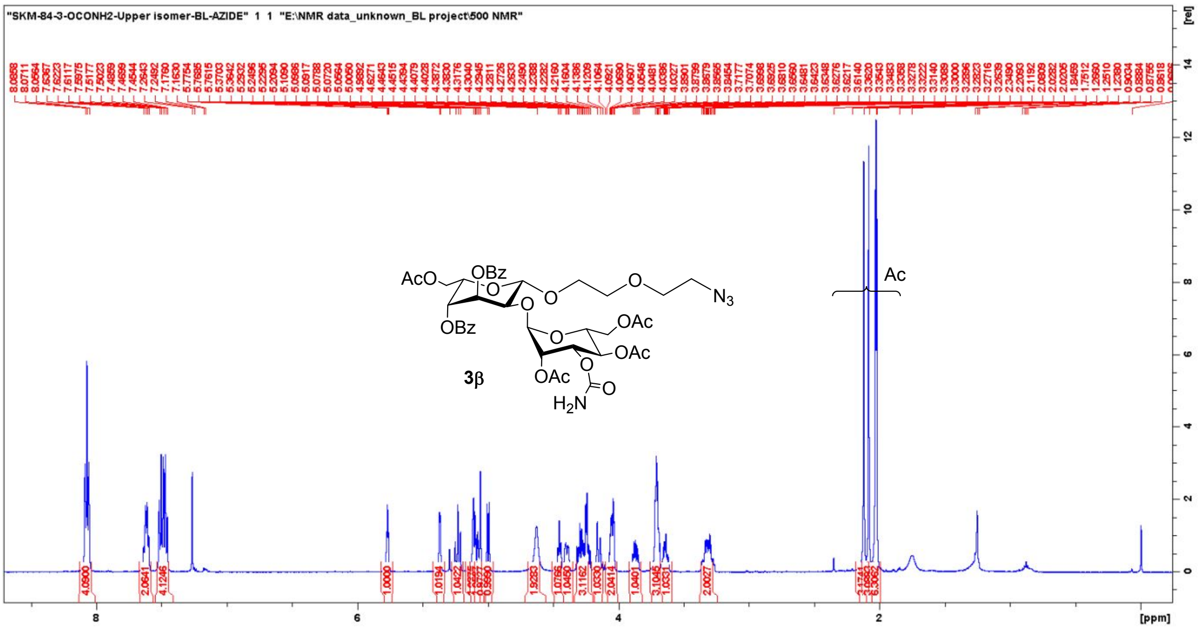

Figure S22. ^{1}H NMR (500 MHz, $CDCl_3$) spectrum of compound **3β**.

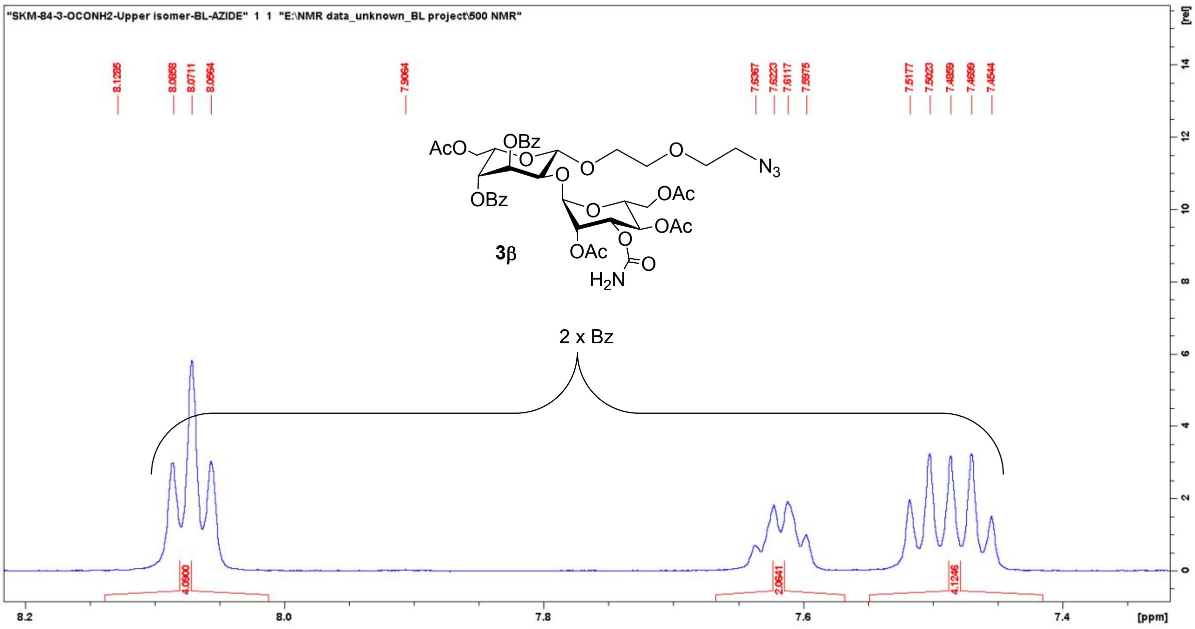

Figure S23. ^{1}H NMR (500 MHz, $CDCl_3$) spectrum of compound **3β**.

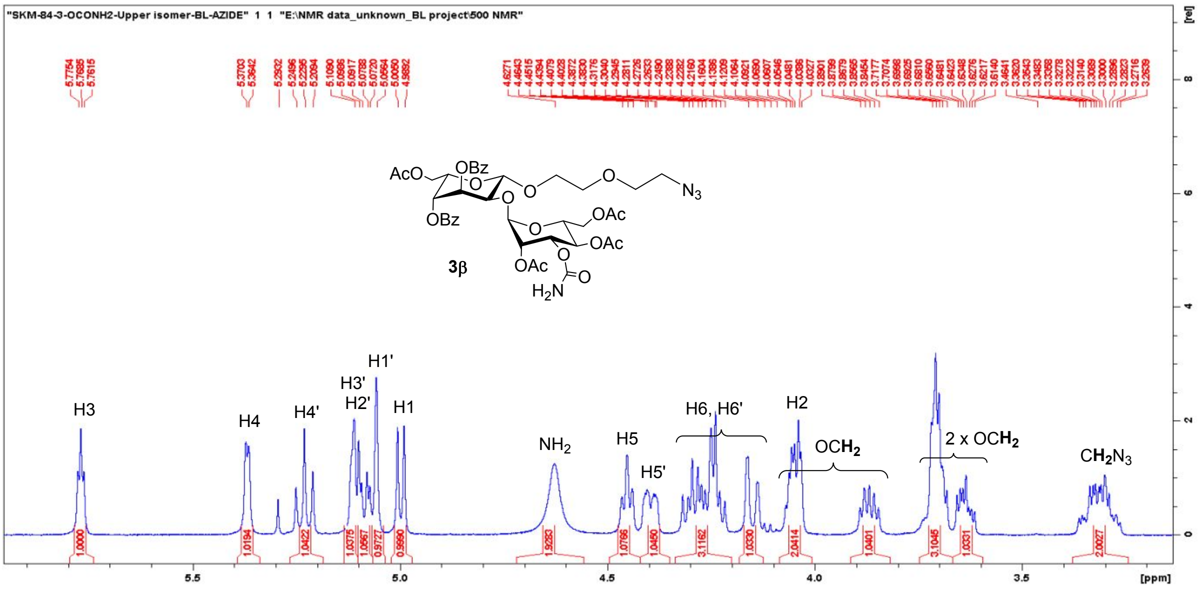

Figure S24. ^{1}H NMR (500 MHz, $CDCl_3$) spectrum of compound **3β**.

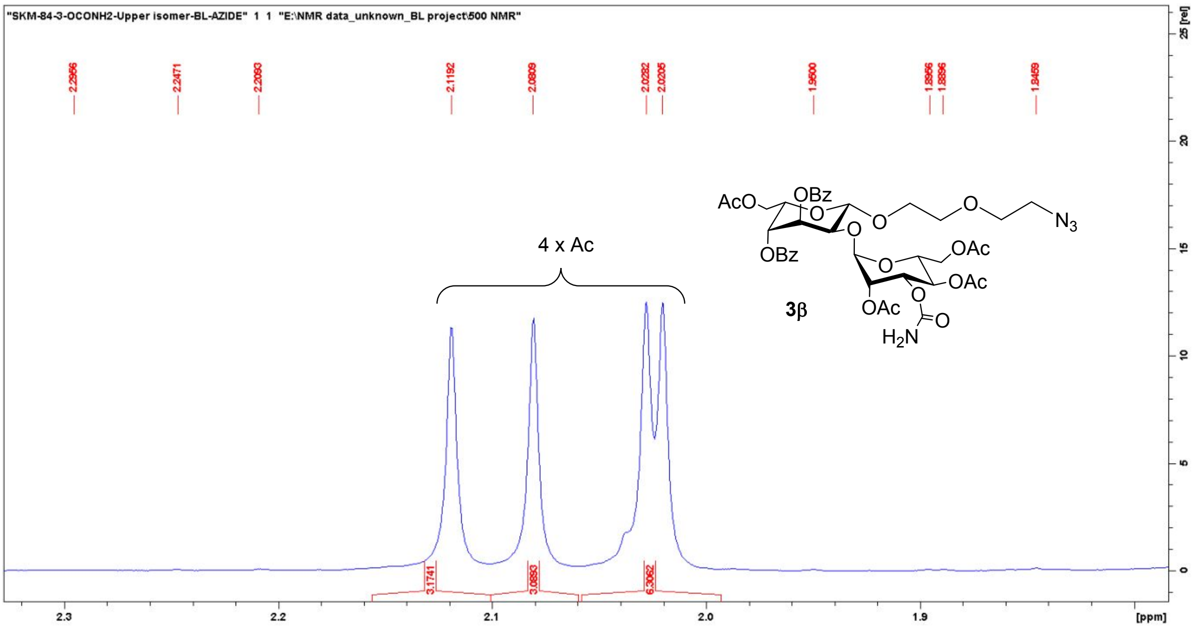

Figure S25. ^{1}H NMR (500 MHz, $CDCl_3$) spectrum of compound **3β**.

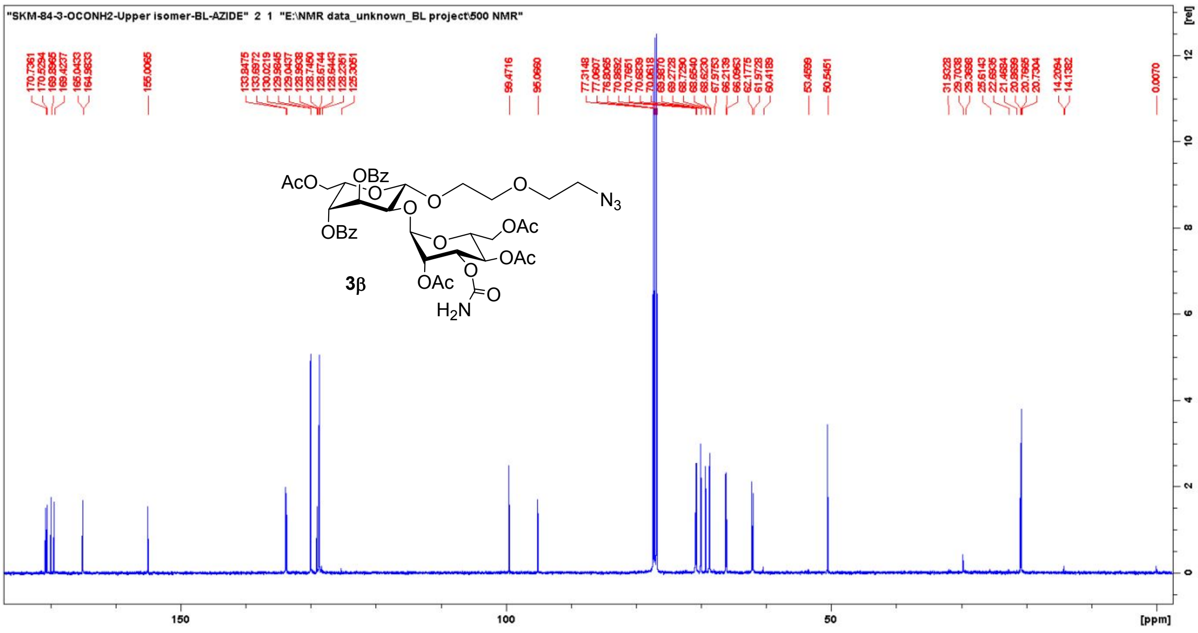

Figure S26. ^{13}C NMR (125 MHz, $CDCl_3$) spectrum of compound **3β**.

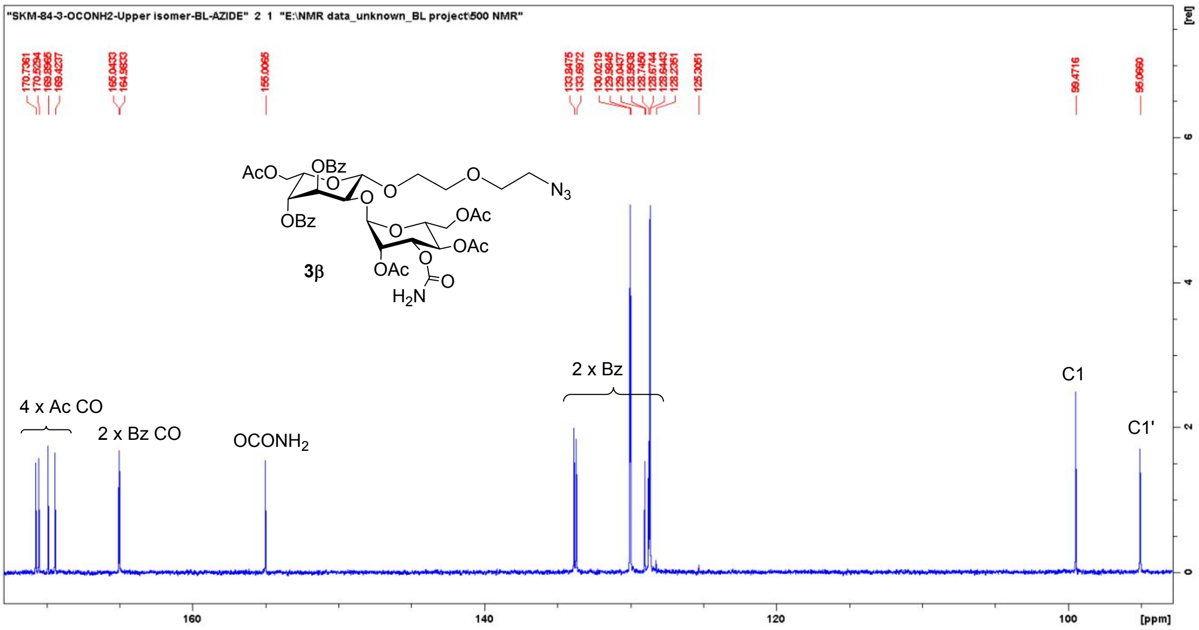

Figure S27. ^{13}C NMR (125 MHz, $CDCl_3$) spectrum of compound **3β**.

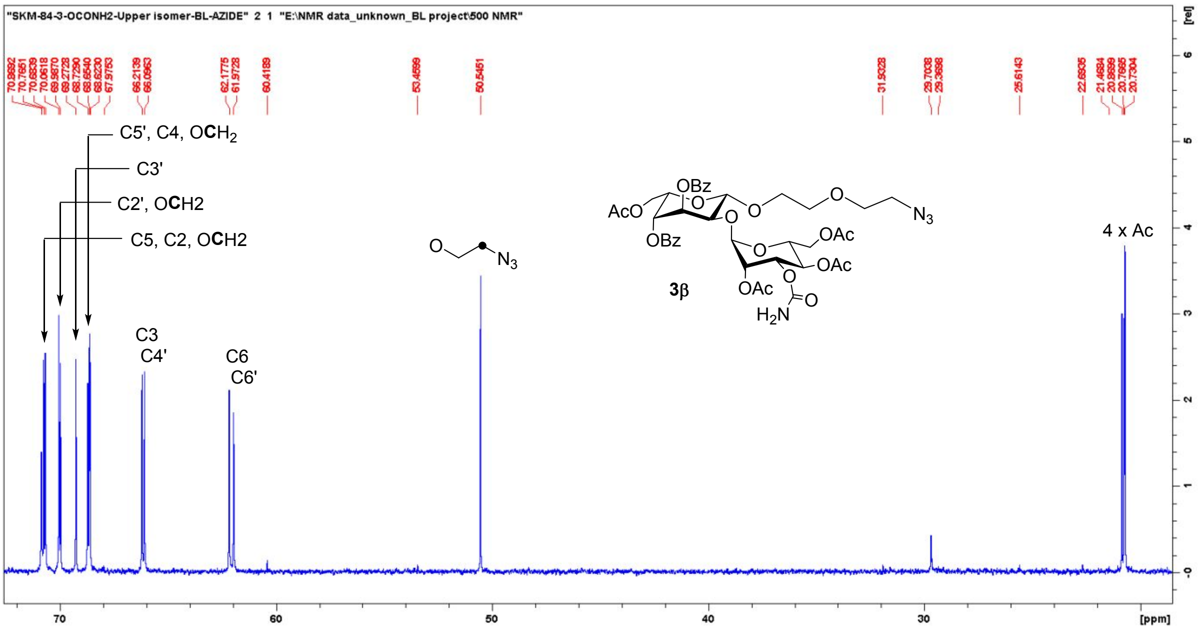

Figure S28. ^{13}C NMR (125 MHz, $CDCl_3$) spectrum of compound **3β**.

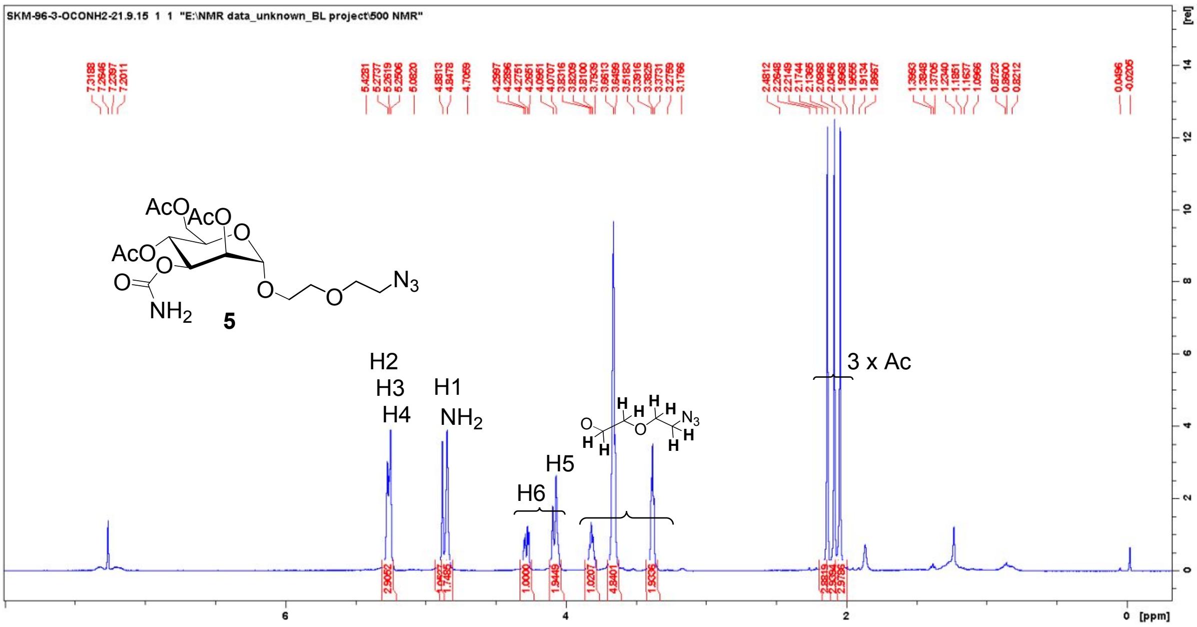

Figure S29. ^{1}H NMR (500 MHz, $CDCl_3$) spectrum of compound **5**.

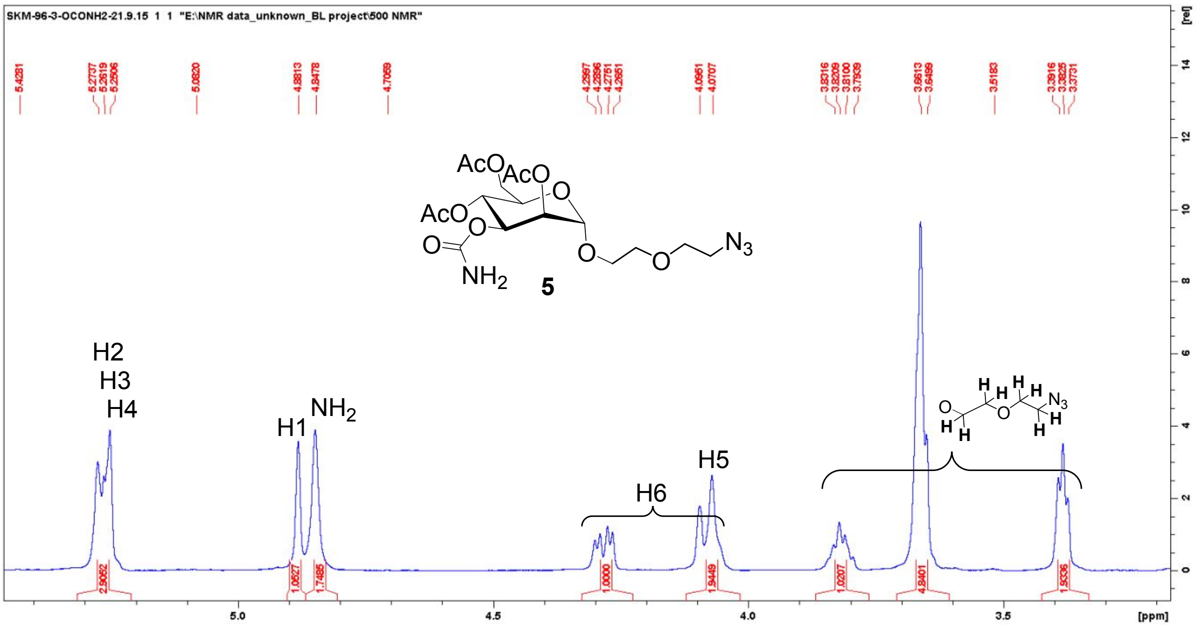

Figure S30. ^{1}H NMR (500 MHz, $CDCl_3$) spectrum of compound **5**.

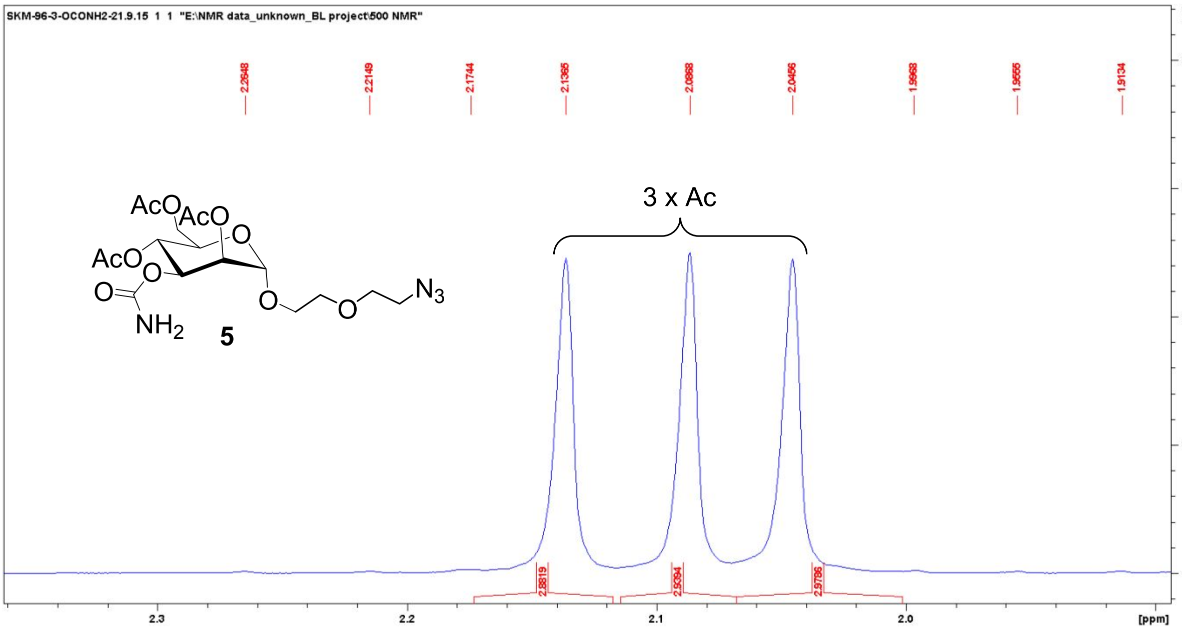

Figure S31. ^{1}H NMR (500 MHz, $CDCl_3$) spectrum of compound **5**.

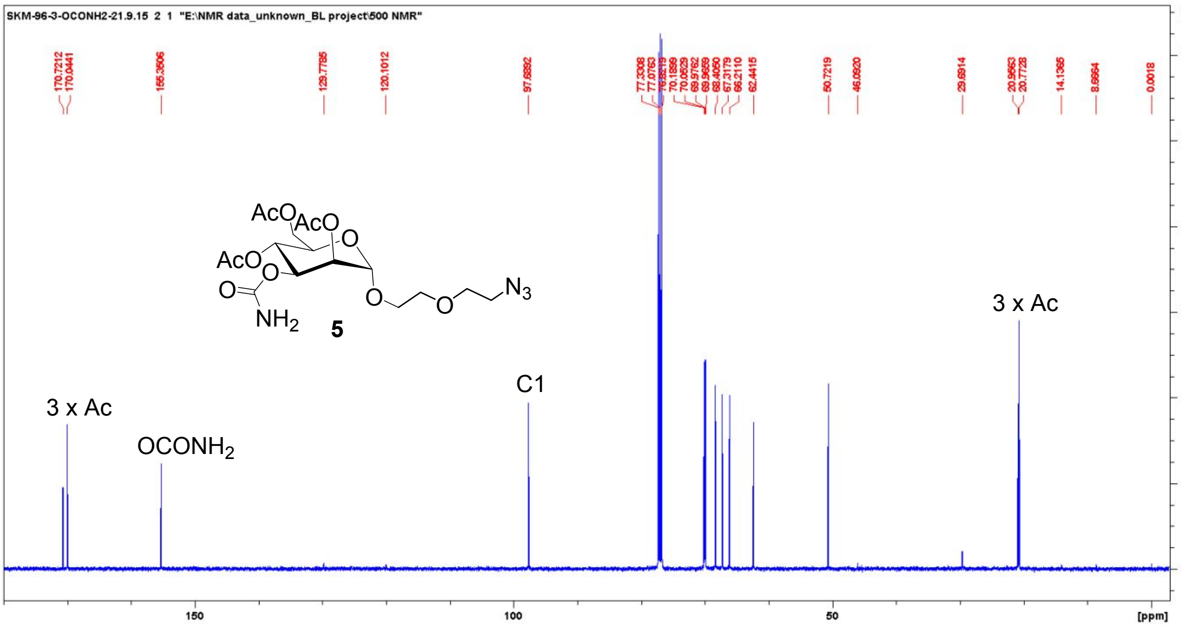

Figure S32. ^{13}C NMR (125 MHz, $CDCl_3$) spectrum of compound **5**.

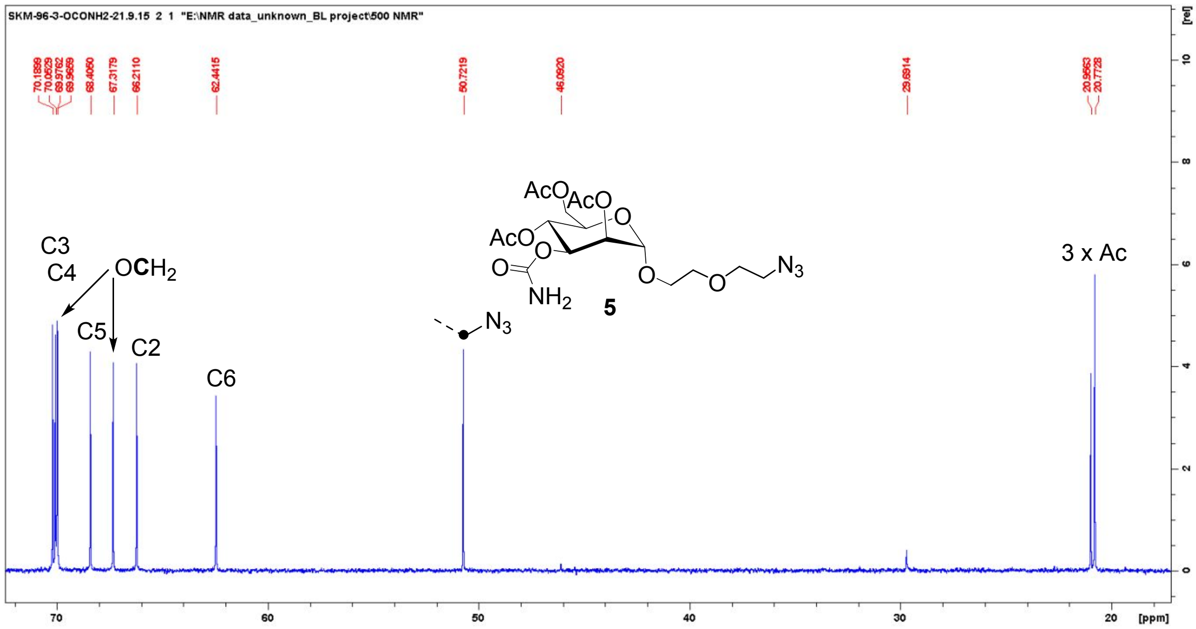

Figure S33. ^{13}C NMR (125 MHz, $CDCl_3$) spectrum of compound **5**.

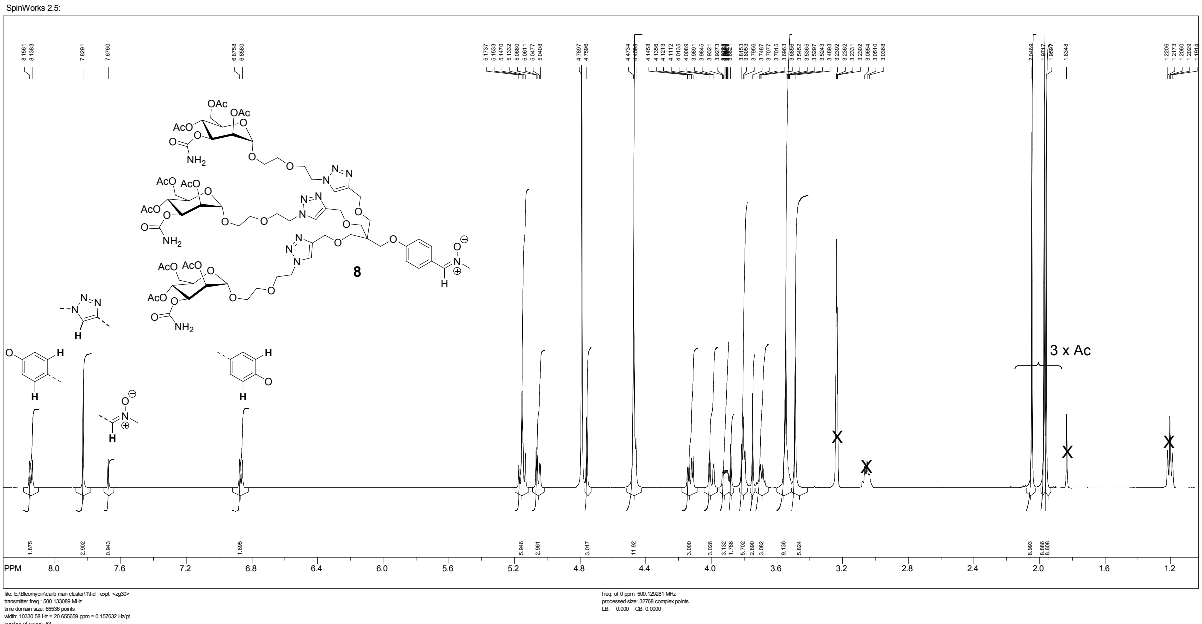

Figure S34. ^{1}H NMR (500 MHz, CD3OD) spectrum of compound **8**.

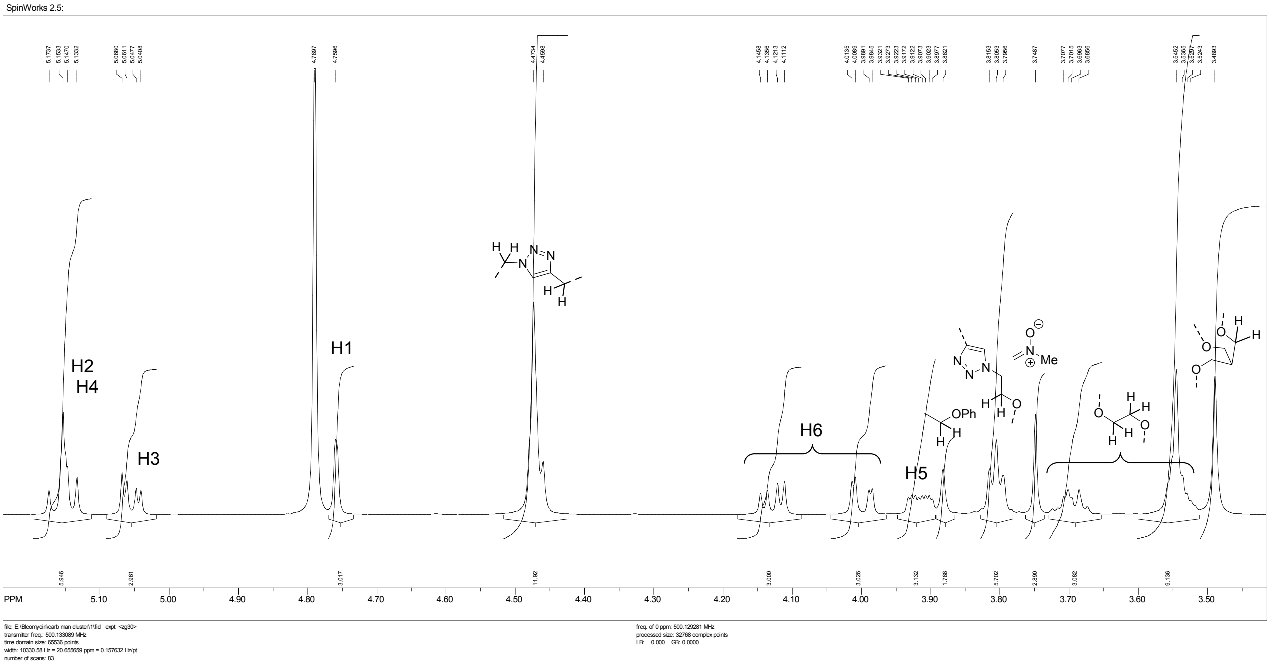

Figure S35. ^{1}H NMR (500 MHz, CD_3OD) spectrum of compound **8**.

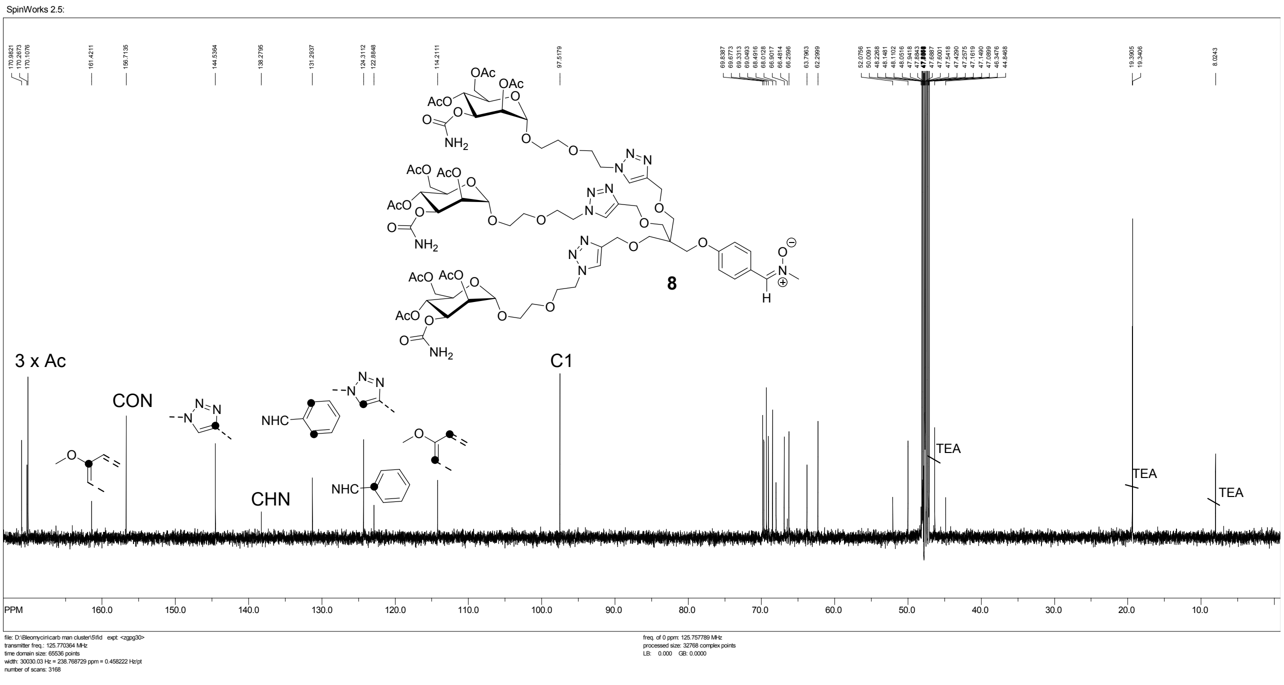

Figure S36. ^{13}C NMR (125 MHz, CD_3OD) spectrum of compound **8**.

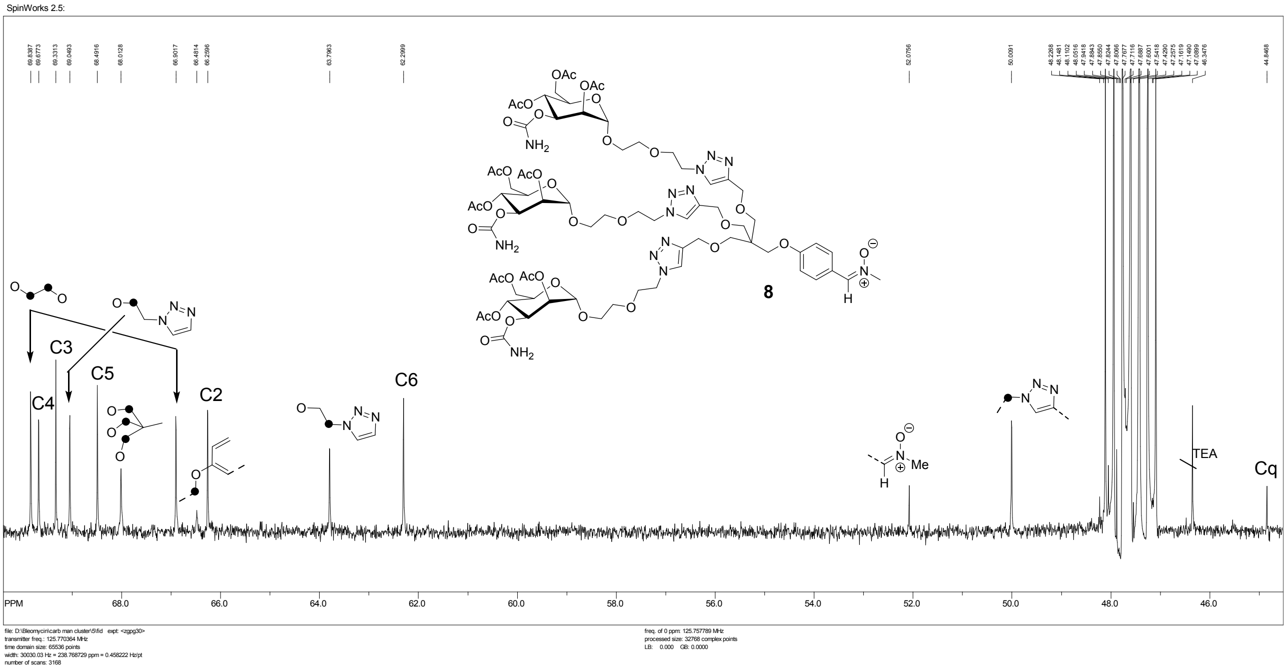

Figure S37. ^{13}C NMR (125 MHz, CD_3OD) spectrum of compound **8**.

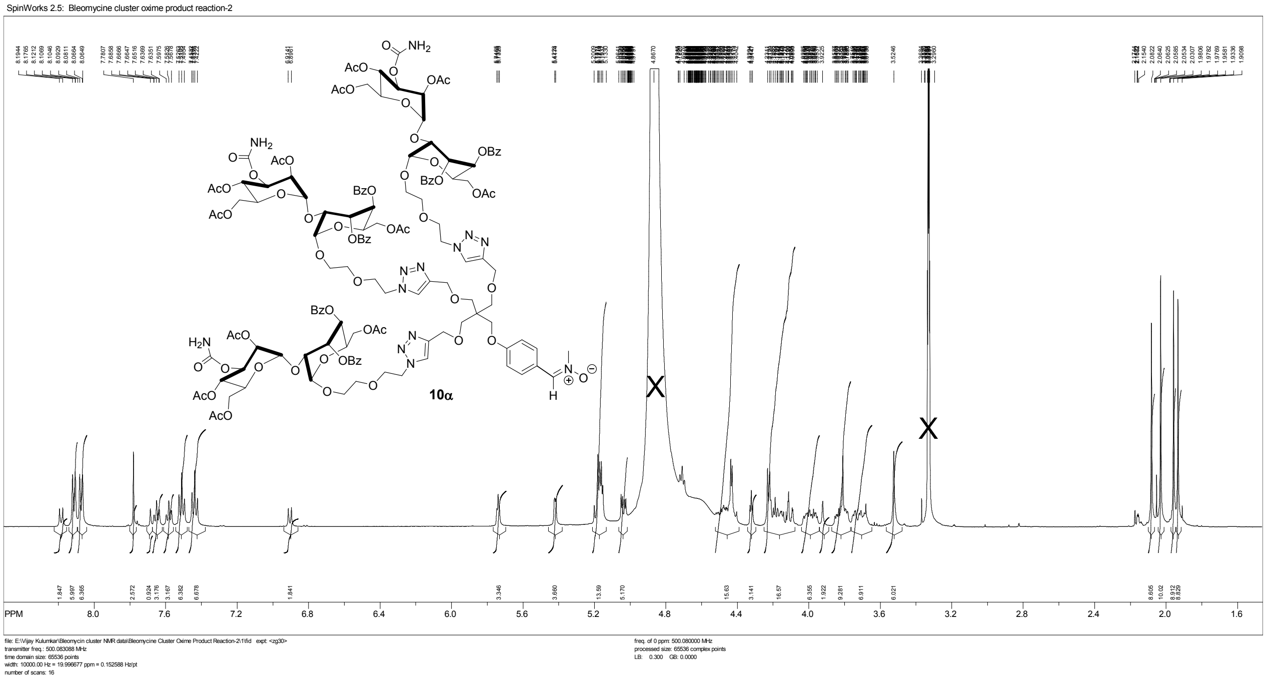

Figure S38. ^{1}H NMR (500 MHz, CD_3OD) spectrum of compound **10α**.

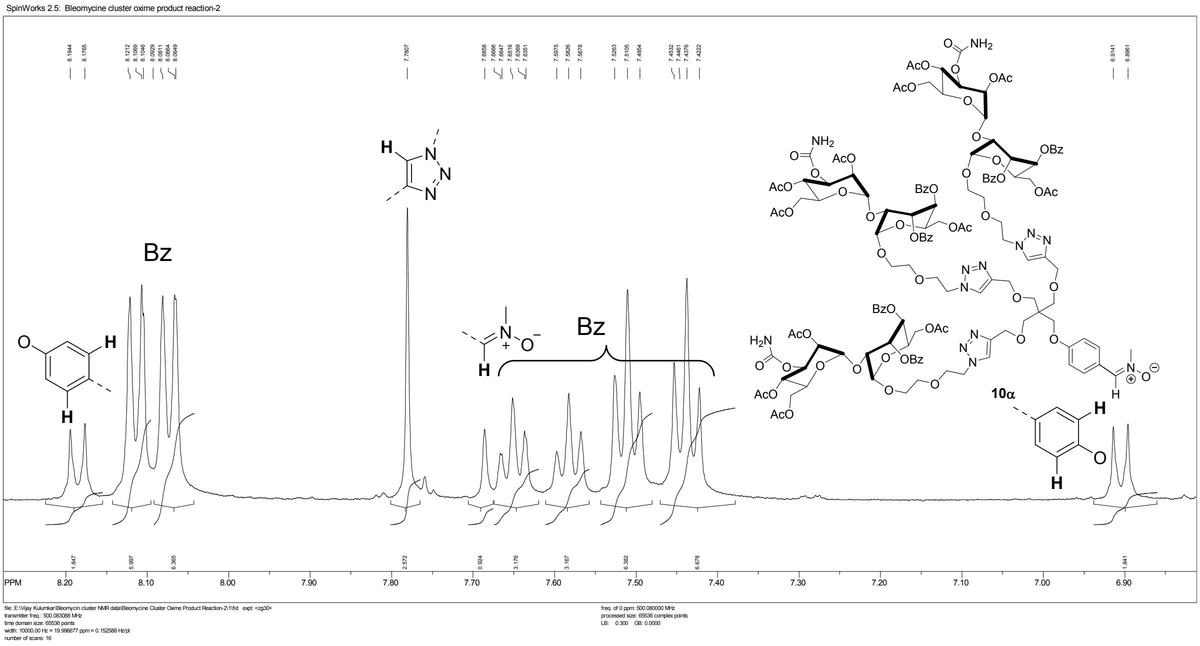

Figure S39. ^{1}H NMR (500 MHz, CD_3OD) spectrum of compound **10α**.

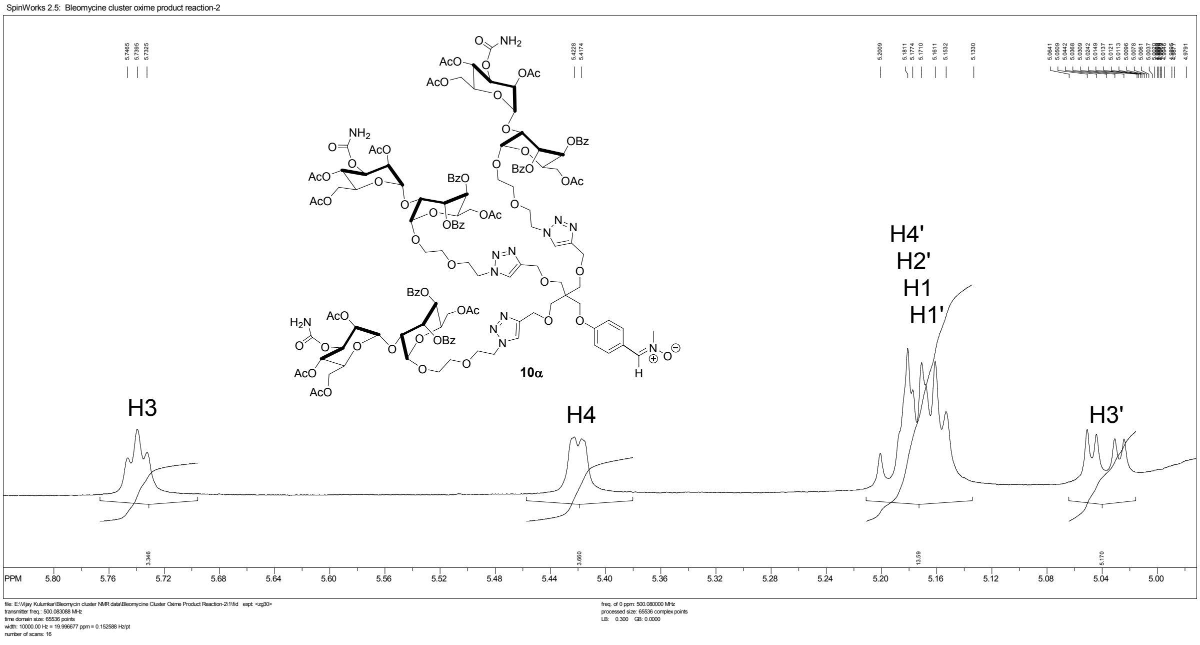

Figure S40. ^{1}H NMR (500 MHz, CD_3OD) spectrum of compound **10α**.

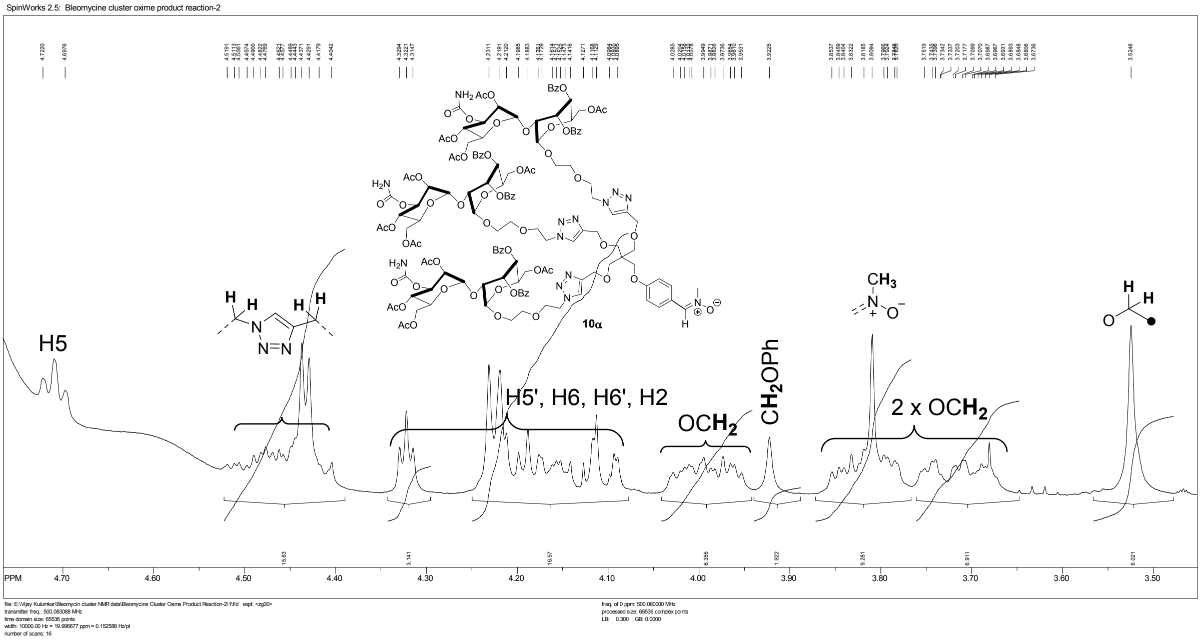

Figure S41. ^{1}H NMR (500 MHz, CD_3OD) spectrum of compound **10α**.

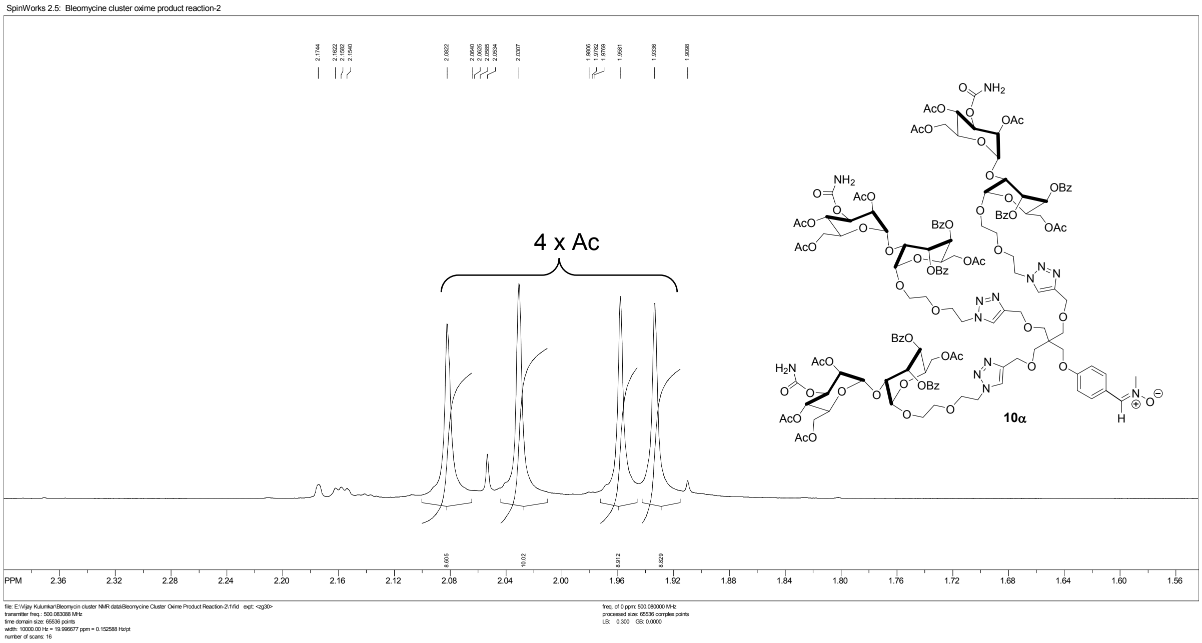

Figure S42. ^{1}H NMR (500 MHz, CD_3OD) spectrum of compound **10α**.

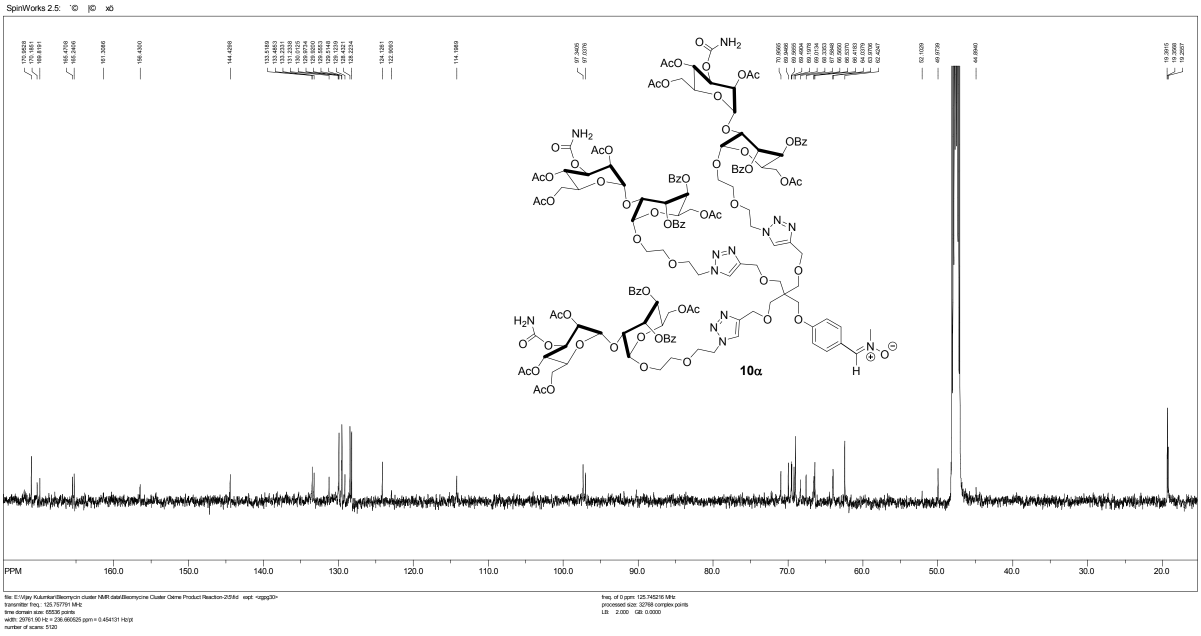

Figure S43. ^{13}C NMR (125 MHz, CD_3OD) spectrum of compound **10α**.

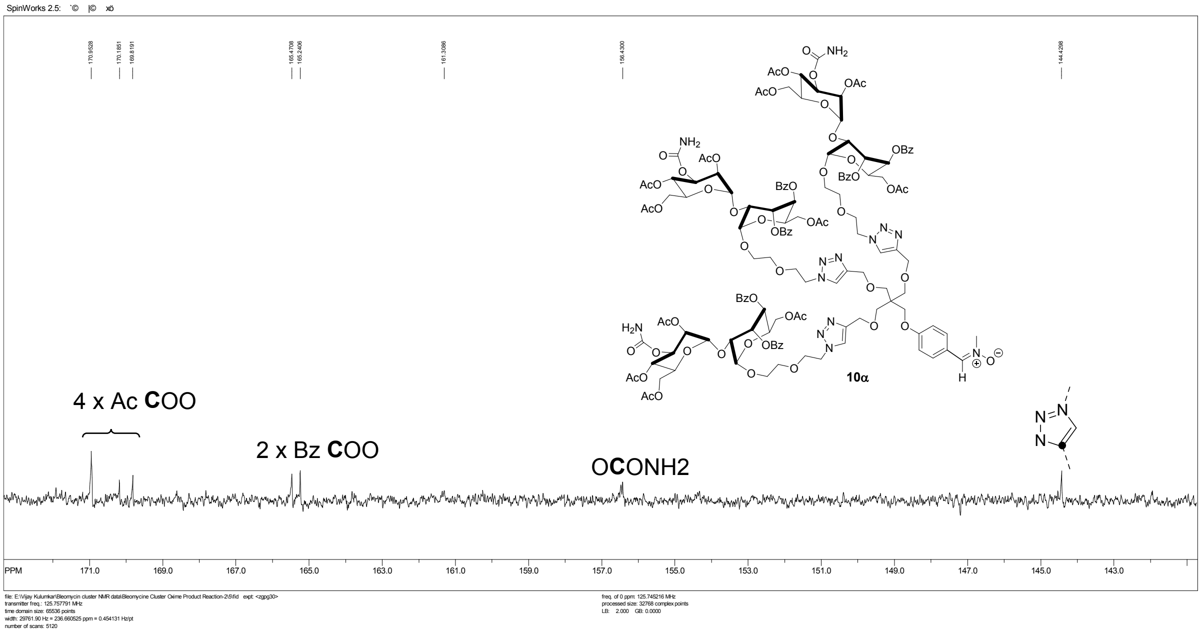

Figure S44. ^{13}C NMR (125 MHz, CD_3OD) spectrum of compound **10α**.

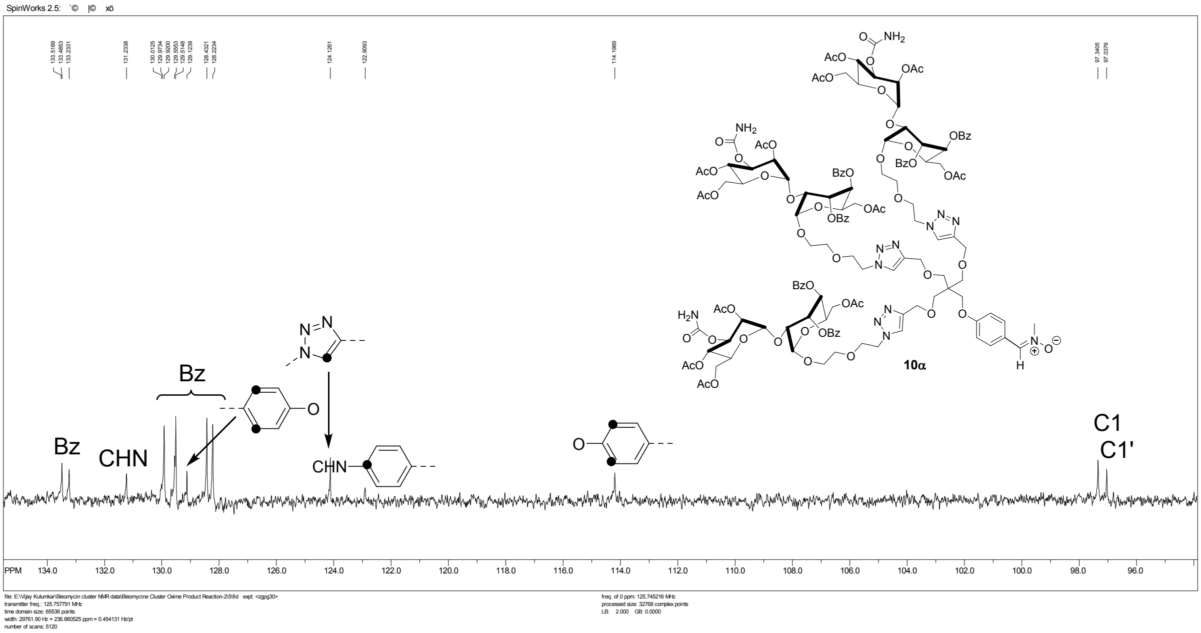

Figure S45. ^{13}C NMR (125 MHz, CD_3OD) spectrum of compound **10α**.

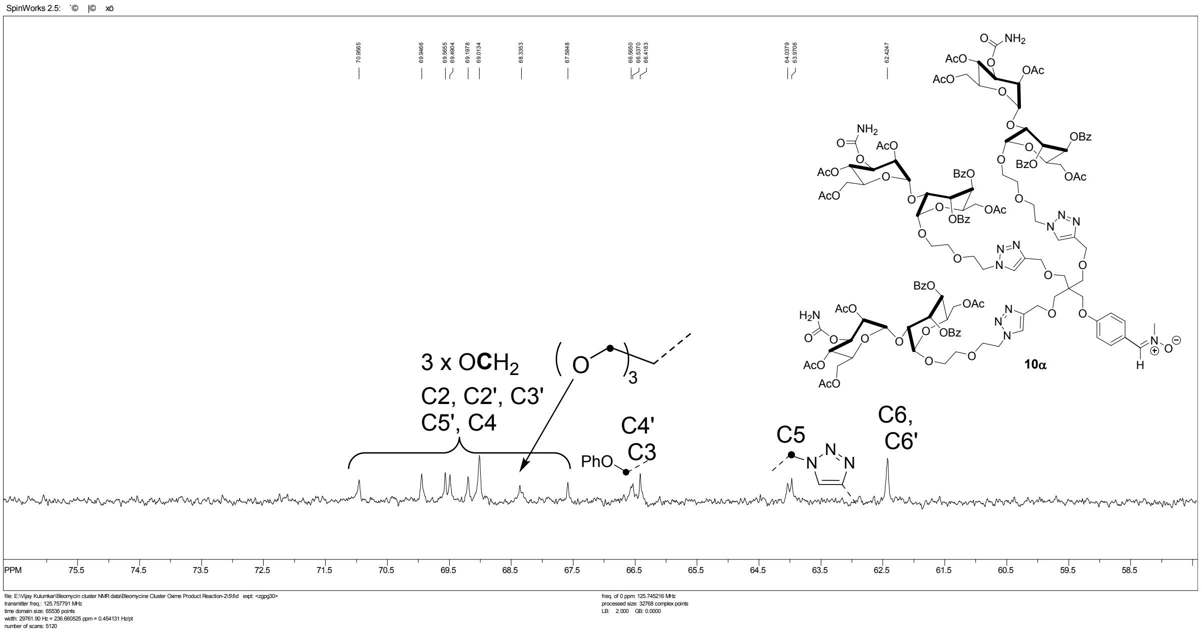

Figure S46. ^{13}C NMR (125 MHz, CD_3OD) spectrum of compound **10α**.

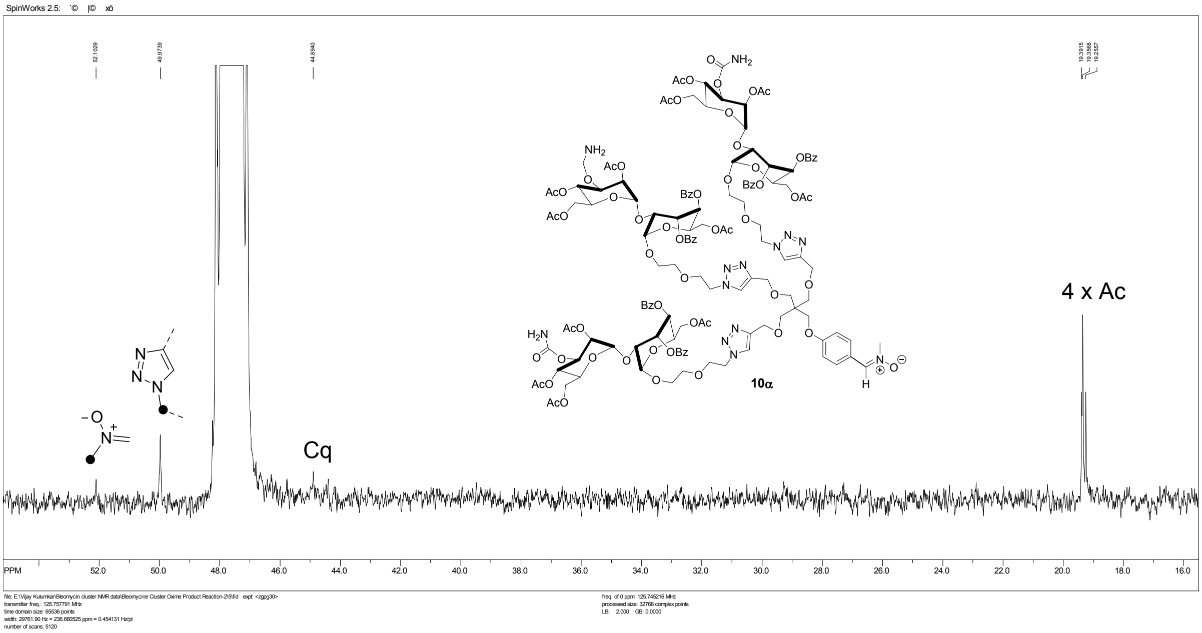

Figure S47. ^{13}C NMR (125 MHz, CD_3OD) spectrum of compound **10α**.

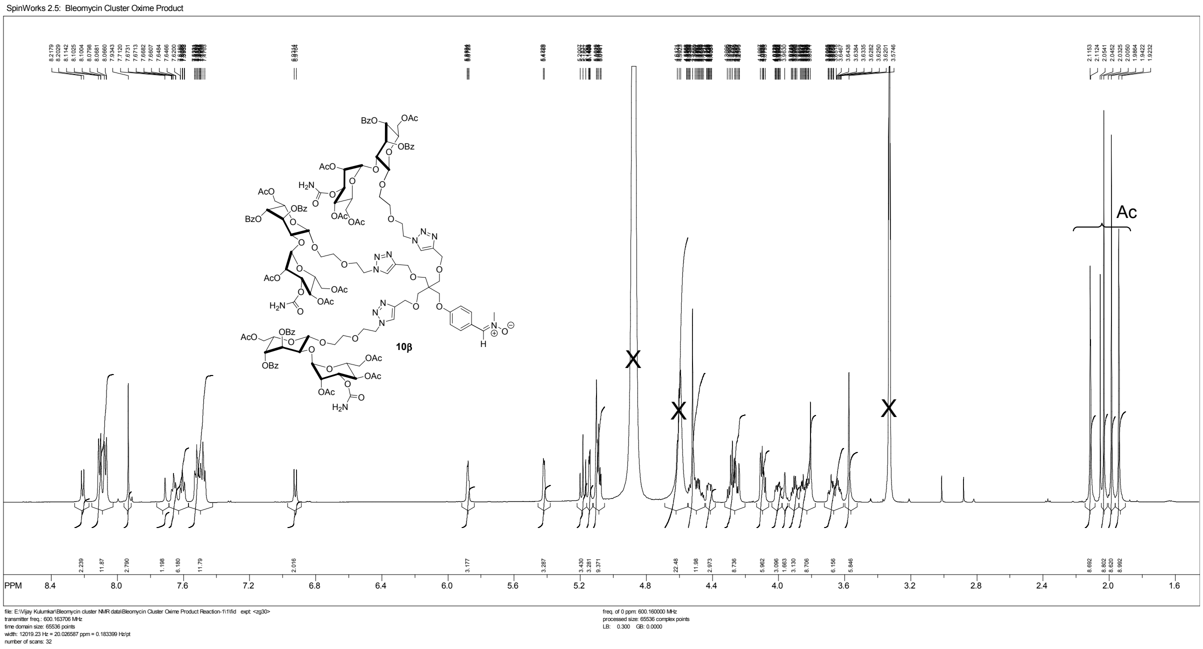

Figure S48. ^{1}H NMR (500 MHz, CD_3OD) spectrum of compound **10β**.

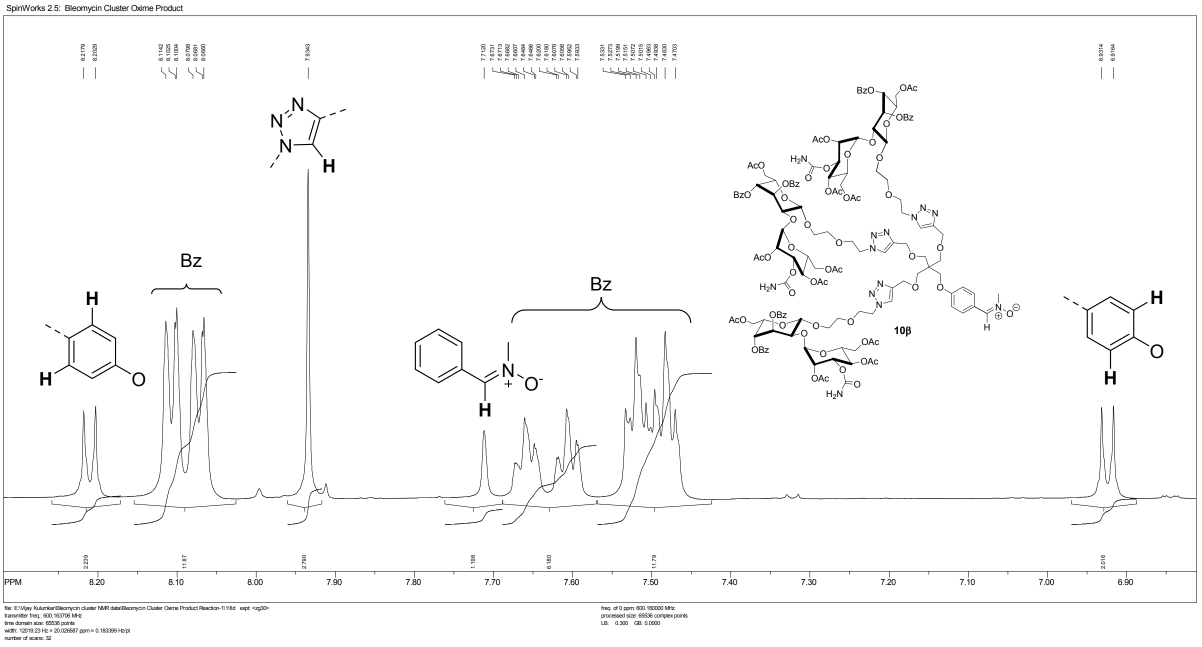

Figure S49. ^{1}H NMR (500 MHz, CD_3OD) spectrum of compound **10β**.

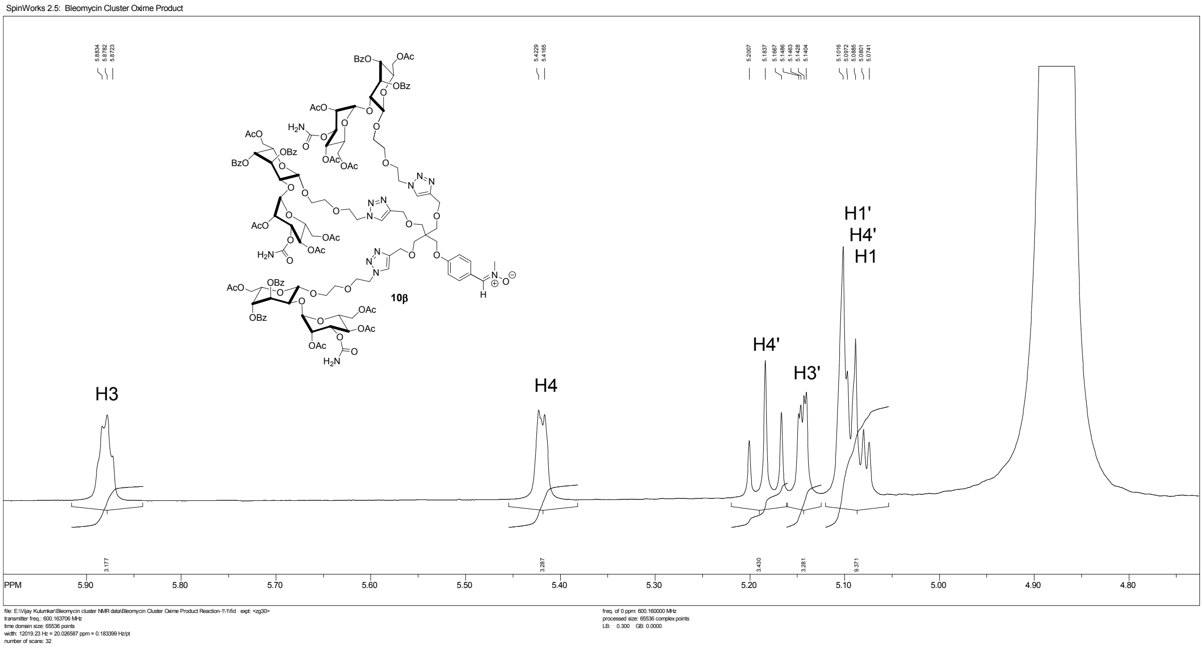

Figure S50. ^{1}H NMR (500 MHz, CD_3OD) spectrum of compound **10β**.

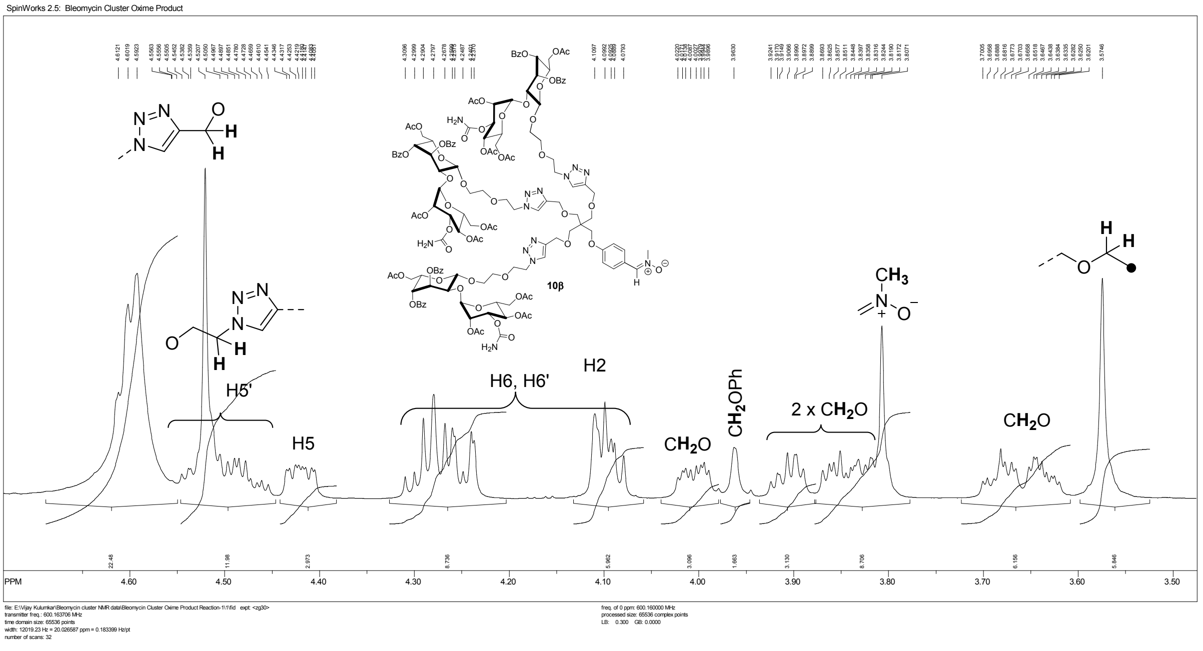

Figure S51. ^{1}H NMR (500 MHz, CD_3OD) spectrum of compound **10β**.

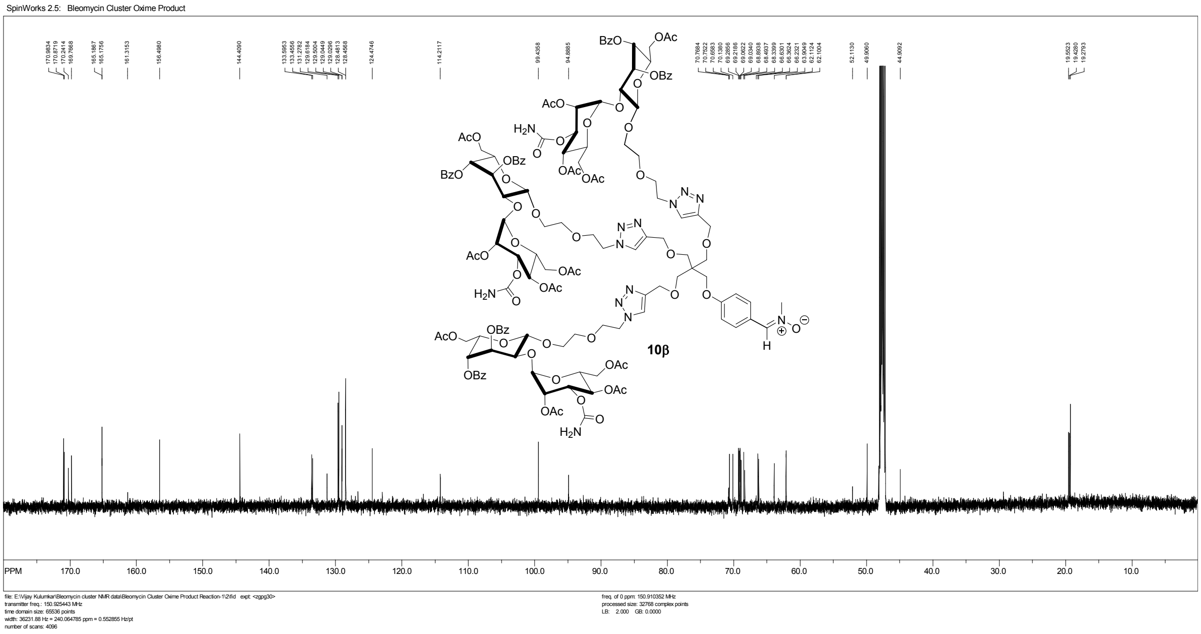

Figure S52. ^{13}C NMR (125 MHz, CD_3OD) spectrum of compound **10β**.

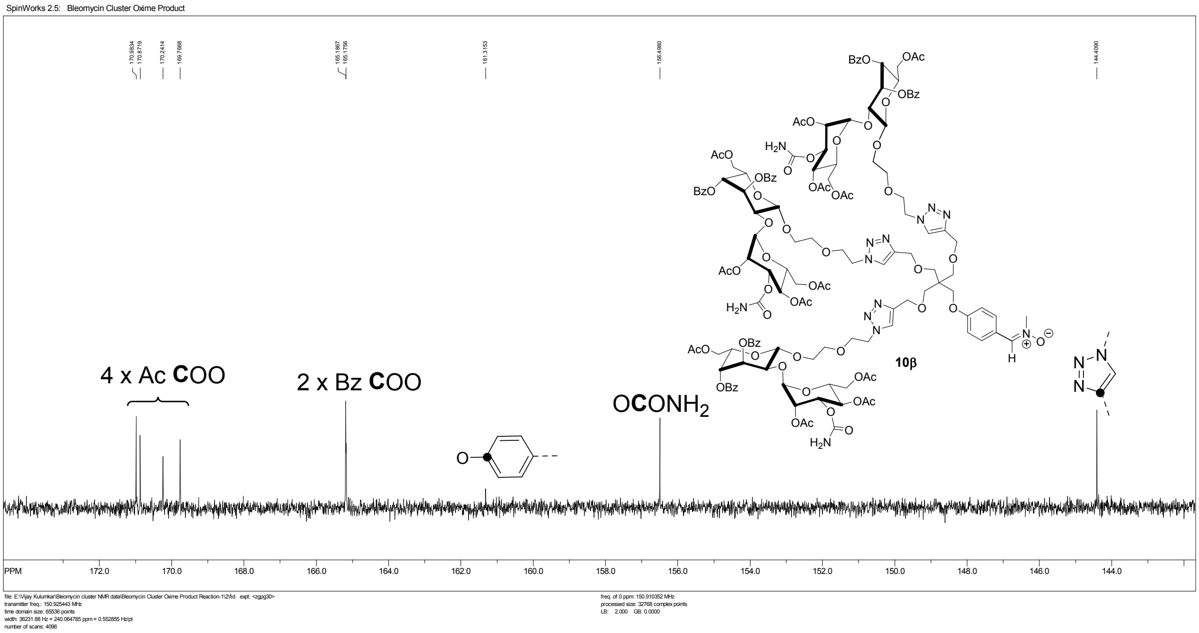

Figure S53. ^{13}C NMR (125 MHz, CD_3OD) spectrum of compound **10β**.

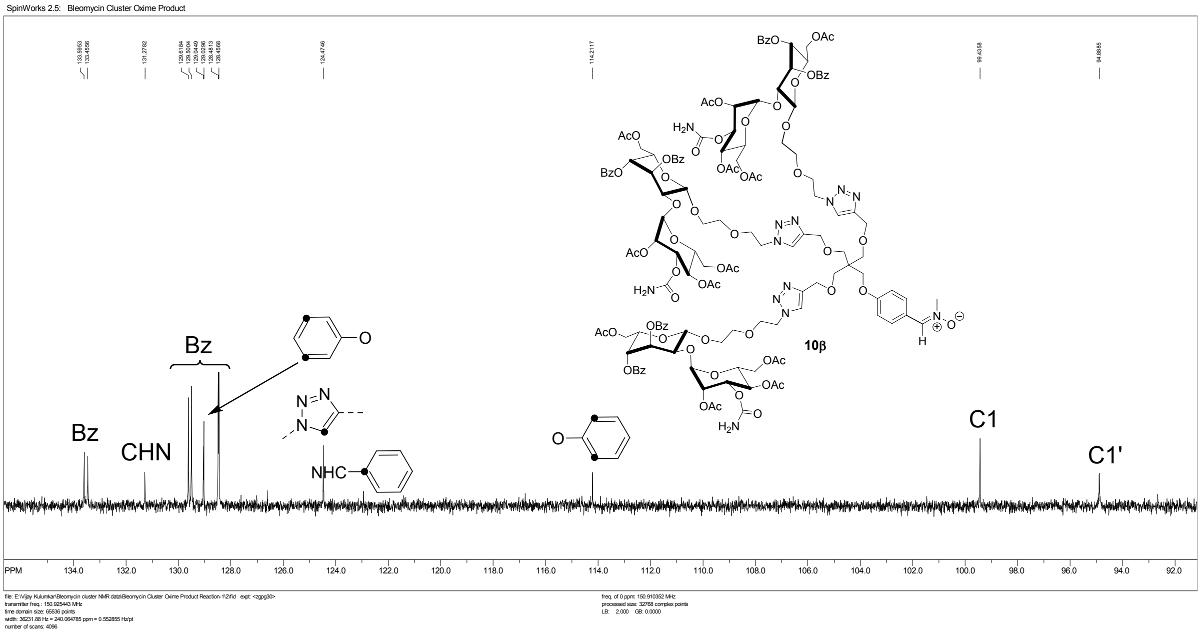

Figure S54. ^{13}C NMR (125 MHz, CD$_3$OD) spectrum of compound **10β**.

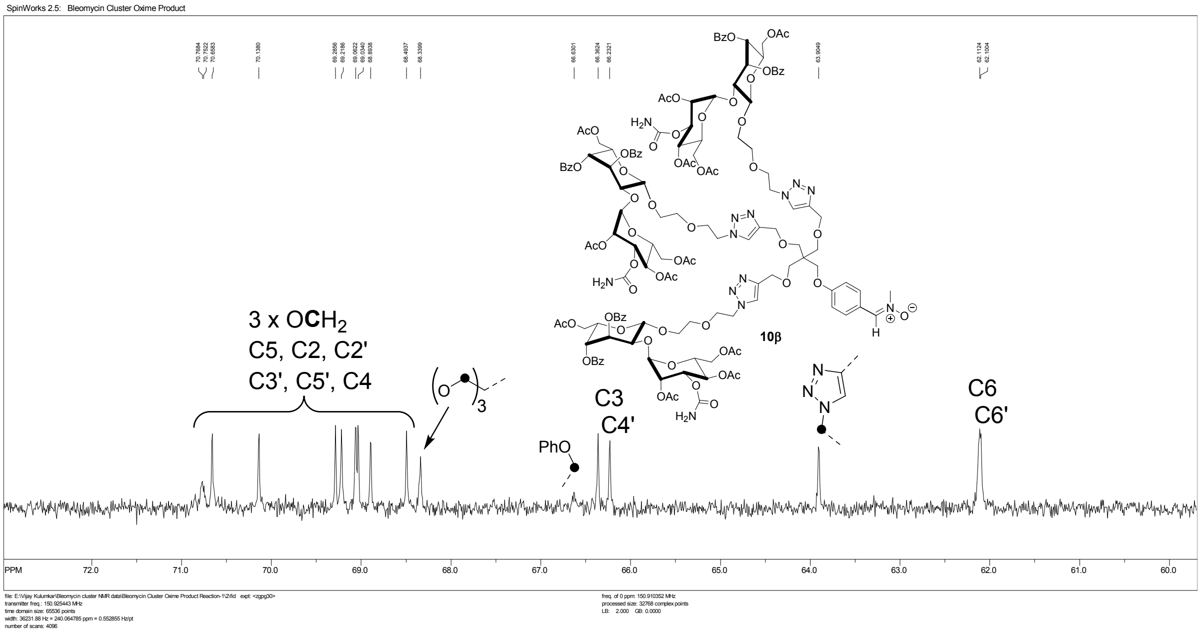

Figure S55. ^{13}C NMR (125 MHz, CD$_3$OD) spectrum of compound **10β**.

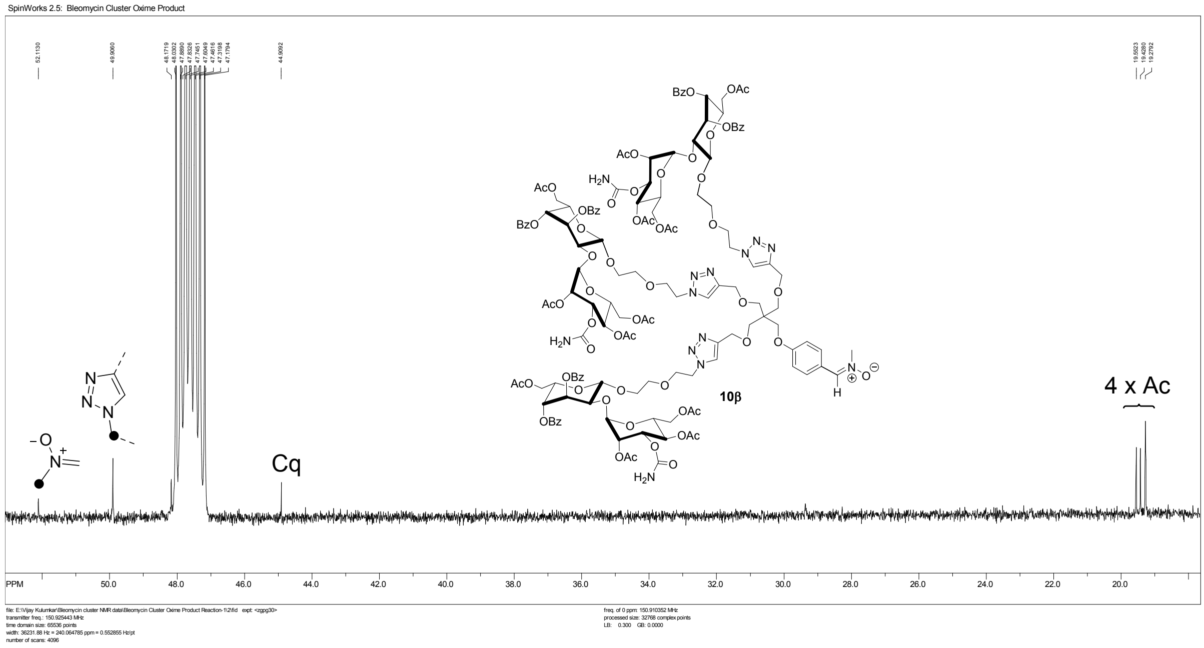

Figure S56. ^{13}C NMR (125 MHz, CD_3OD) spectrum of compound **10β**.

SPANC Labeling of Nitrone-Modified Glycoclusters 11, 12α and 12β with Rhodamine Dye (compounds 13, 14α and 14β)

To test the applicability of the nitrone-modified glycoclusters **11**, **12α** and **12β** for strain-promoted alkyne-nitrone cycloaddition (SPANC) ligation, small molecule trial with dibenzylcyclooctyne(DBCO)-PEG4-5/6-carboxyrhodamine dye was carried out (Scheme S1). To aqueous solutions of compounds **11**, **12α** and **12β**, the DBCO-modified dye (2 equiv.) in DMF was added. The mixture was incubated overnight at room temperature and the formation of the labeled glycoclusters **13**, **14α** and **14β** was confirmed by RP HPLC and mass spectrometry. **13:** HRMS (ESI-TOF): *m/z* $C_{105}H_{135}N_{18}NaO_{39}^{+}$ [(M + H + Na)/2]$^{2+}$ requires 1147.4567, found 1147.4623. **14α:** HRMS (ESI-TOF): *m/z* $C_{123}H_{165}N_{18}NaO_{54}^{2+}$ [(M + H + Na)/2]$^{2+}$ requires 1390.54, found 1390.57. **14β:** HRMS (ESI-TOF): *m/z* $C_{123}H_{165}N_{18}NaO_{54}^{2+}$ [(M + H + Na)/2]$^{2+}$ requires 1390.54, found 1390.56.

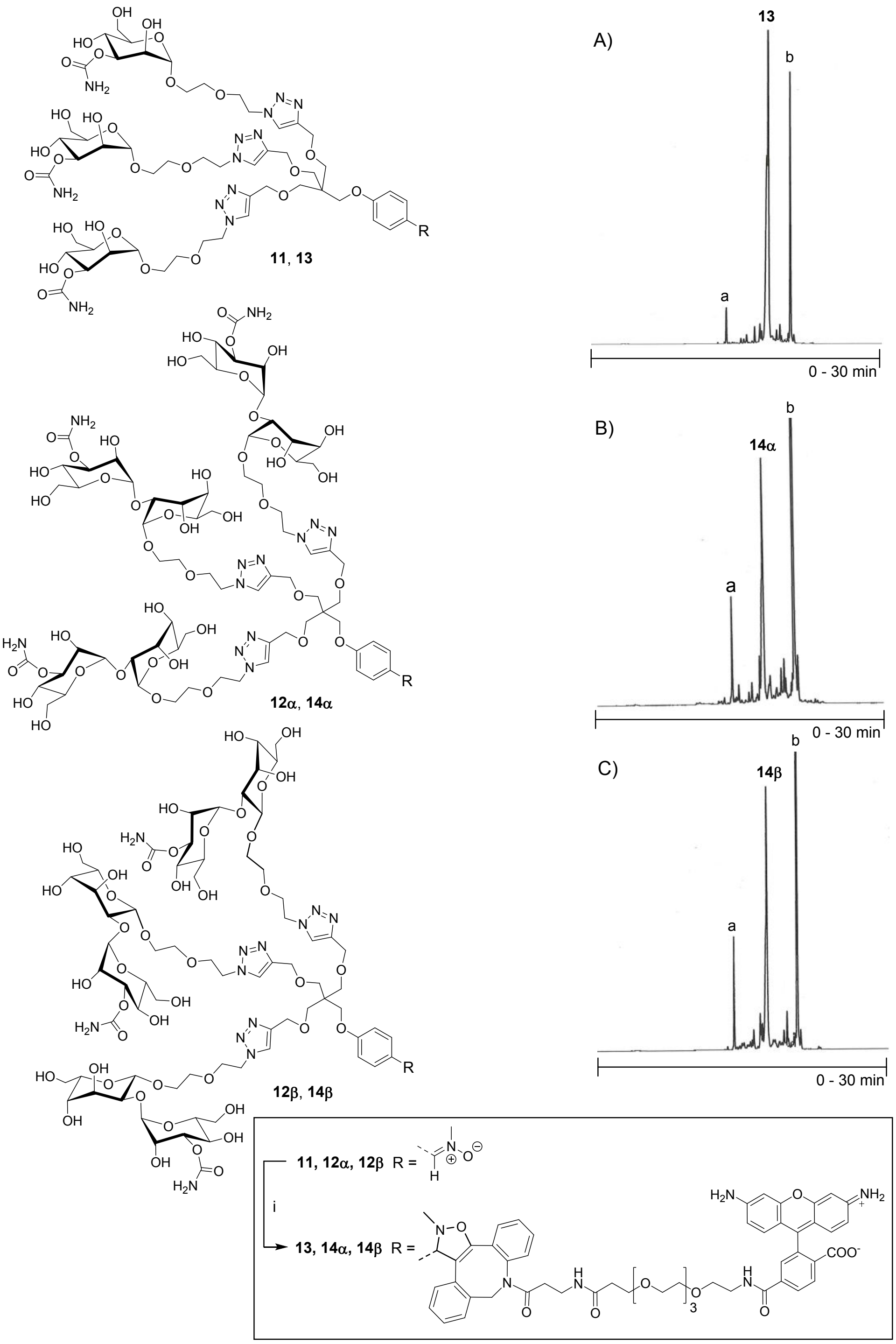

Scheme S1. SPANC labeling of nitrone-modified glycoclusters with a rhodamine dye. Reagents and conditions: i) dibenzylcyclooctyne-PEG4-5/6-carboxyrhodamine dye (2 eq), DMF-H_2O (2:1), overnight at room temperature; A-C: RP HPLC chromatograms of crude product (**13**, **14α** and **14β**) mixtures, a = byproduct of the dye, b = the dye. RP-HPLC conditions: An analytical C-18 column (250 × 4.6 mm, 5 µm), flow rate: 1.0 mL min^{-1}, detection at 501 nm, a gradient elution from 0 to 100% MeCN in 0.1 mol L^{-1} aqueous triethylammonium acetate over 30 min.

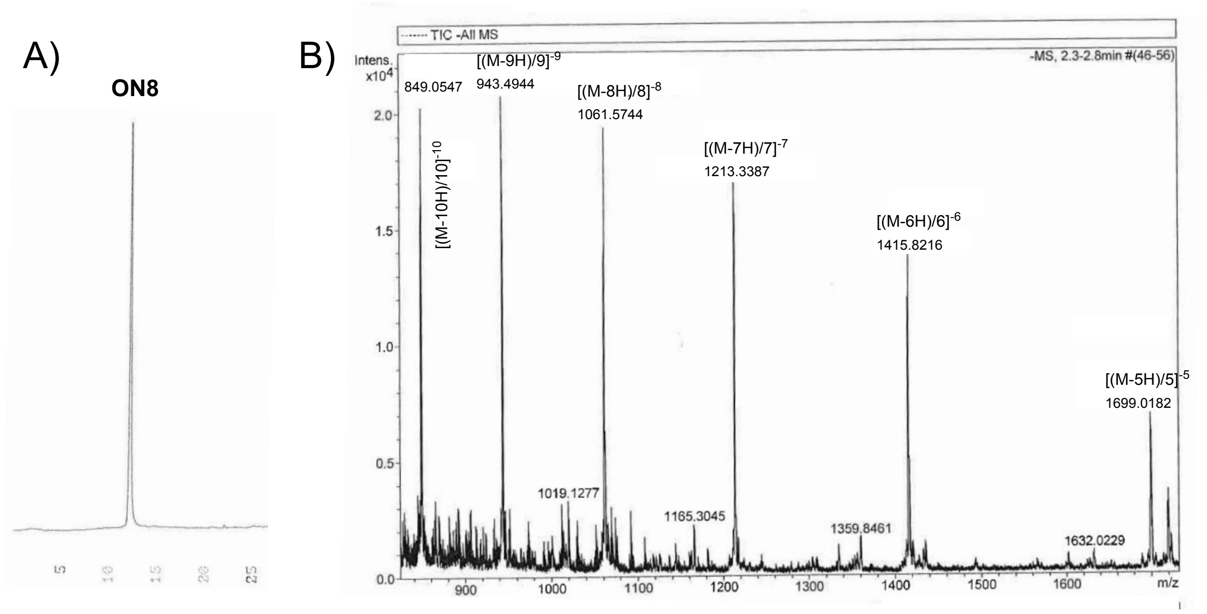

Figure S57. A) RP HPLC profile and B) MS (ESI-TOF) spectrum of **ON8**. RP HPLC conditions: An analytical RP column (250 × 4.6 mm, 5 µm), detection at λ = 260 nm, gradient elution (0– 25 min) from 5 to 95% MeCN in 0.1 M triethylammonium acetate, flow rate 1.0 mL min^{-1}.

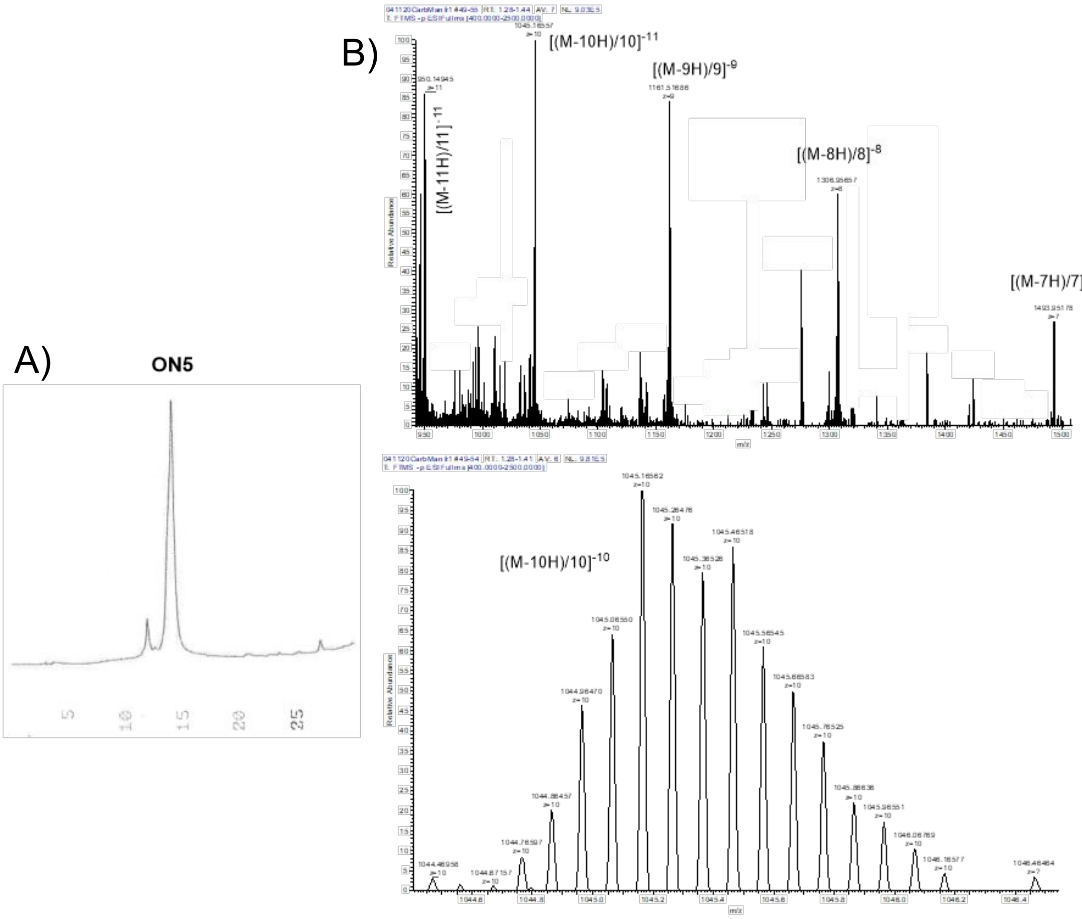

Figure S58. A) RP HPLC profile and B) MS (orbitrap) spectrum of **ON5**. RP HPLC conditions: An analytical RP column (250 × 4.6 mm, 5 µm), detection at λ = 260 nm, gradient elution (0– 25 min) from 5 to 95% MeCN in 0.1 M triethylammonium acetate, flow rate 1.0 mL min^{-1}.

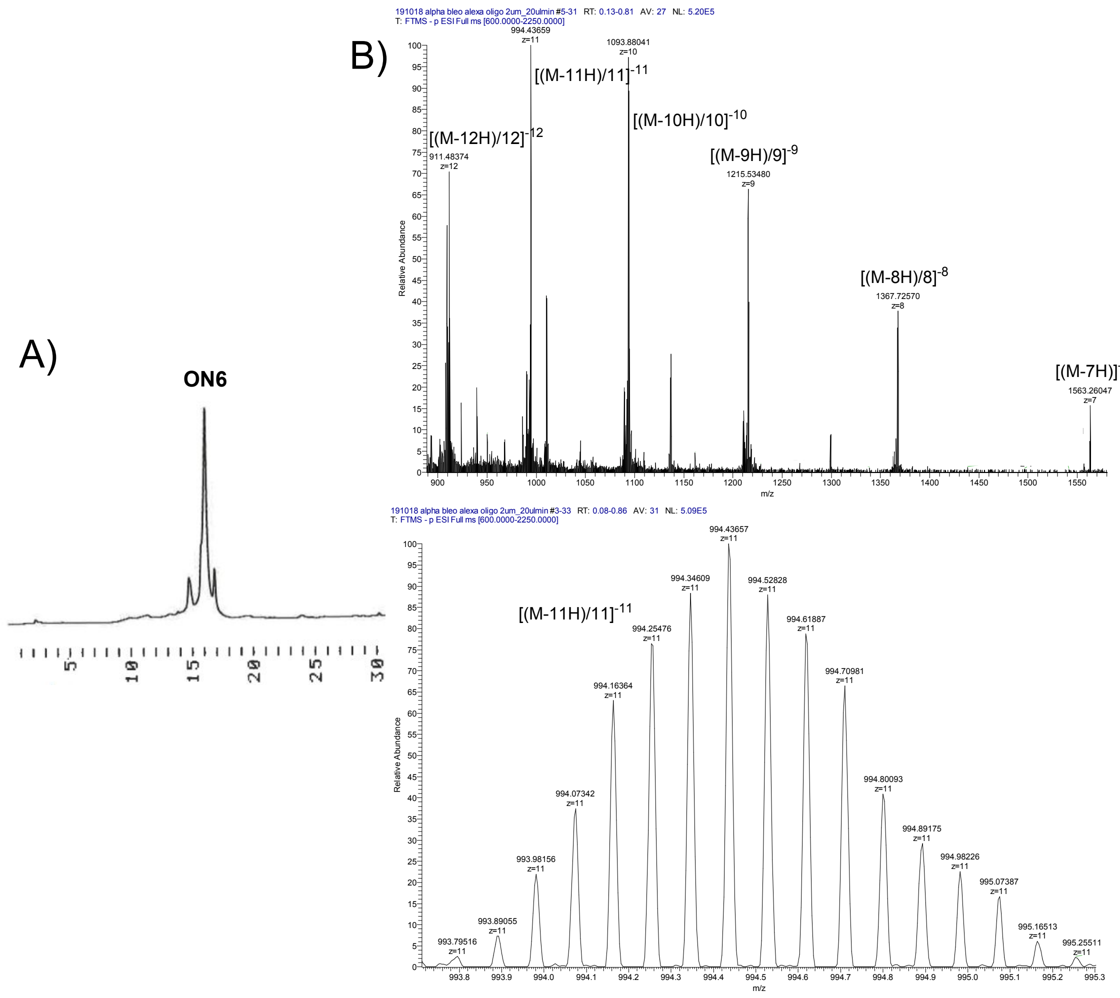

Figure S59. A) RP HPLC profile and B) MS (orbitrap) spectrum of **ON6**. RP HPLC conditions: An analytical RP column (250 × 4.6 mm, 5 µm), detection at λ = 260 nm, gradient elution (0– 25 min) from 5 to 95% MeCN in 0.1 M triethylammonium acetate, flow rate 1.0 mL min^{-1}. * = cleavage products of **ON6**.

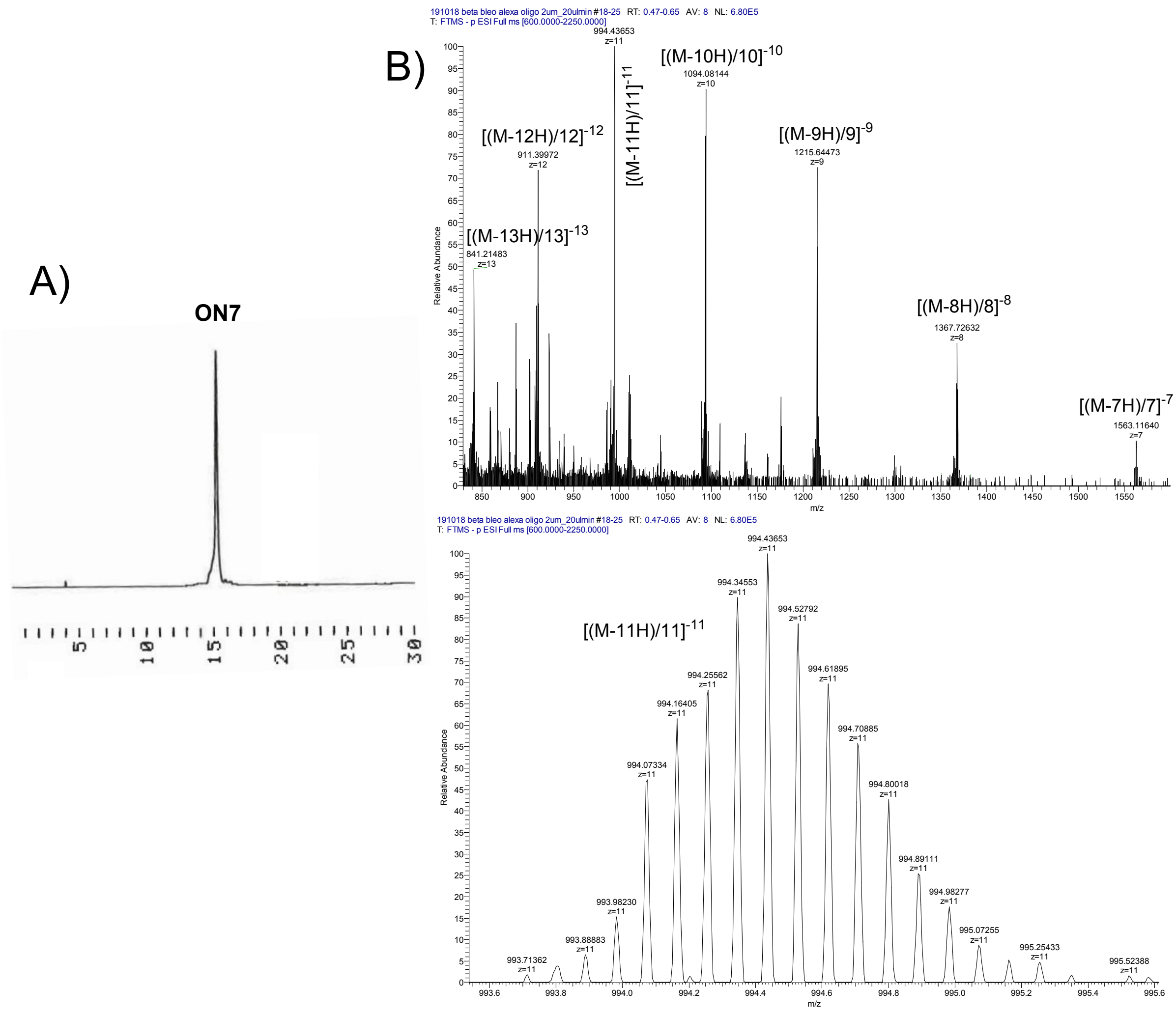

Figure S60. A) RP HPLC profile and B) MS (orbitrap) spectrum of **ON7**. RP HPLC conditions: An analytical RP column (250 × 4.6 mm, 5 µm), detection at λ = 260 nm, gradient elution (0– 25 min) from 5 to 95% MeCN in 0.1 M triethylammonium acetate, flow rate 1.0 mL min^{-1}.

Table S1. MS data of AF488-labeled oligoribonucleotides.

Oligonucleotide	observed mass	calculated mass
**ON8**	8500.9*	8501.0*
**ON5**	10457.7	10457.1
**ON6**	10943.9	10943.3
**ON7**	10943.9	10943.3

*The observed and calculated molecular mass values for **AF488-ISE** are reported according to average masses (ESI-TOF). For the glycocluster–oligonucleotide conjugates **ON5–7**, the molecular mass values are reported according to monoisotopic masses (orbitrap).

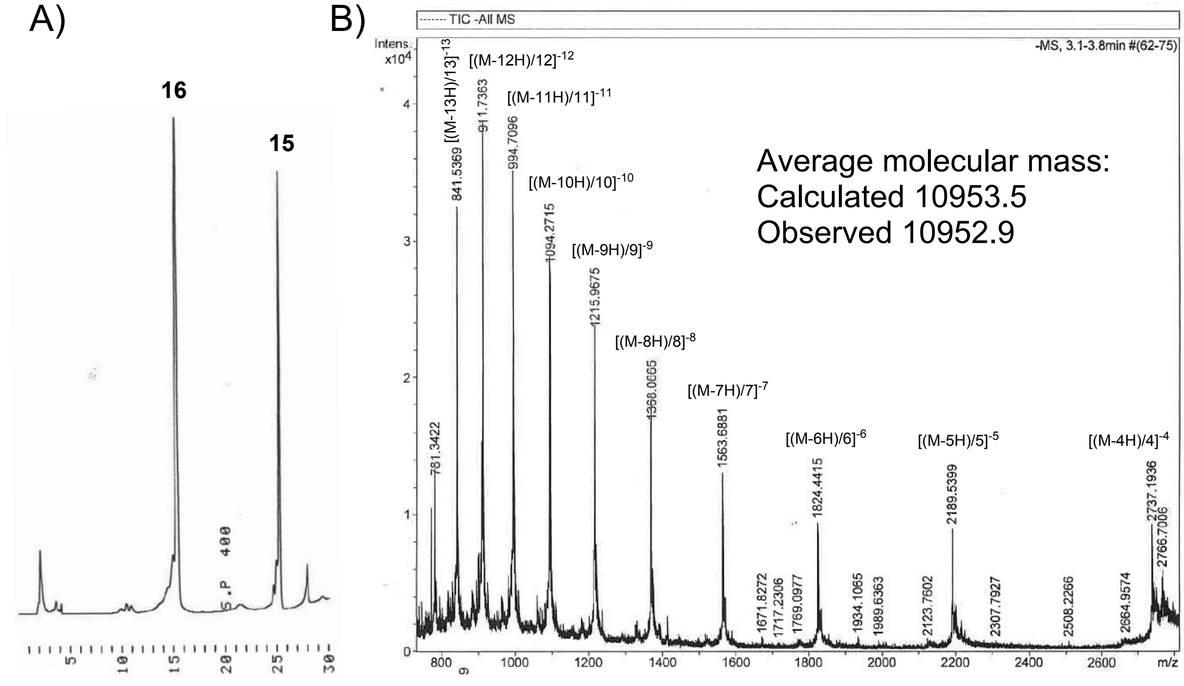

Figure S61. A) Crude RP HPLC profile of the monosubstituted SNA (compound **16**). B) MS (ESI-TOF) spectrum of homogenized monosubstituted SNA. RP HPLC conditions: A) An analytical RP column (250 × 4.6 mm, 5 µm), detection at λ = 260 nm, gradient elution (0–20 min) from 40 to 100% MeCN, 20–30 min 100% MeCN in 50 mM triethylammonium acetate, flow rate 1.0 mL min^{-1}.

Table S2. Fluorescence properties of **ON5–ON8** in the absence and presence of **SNA1**: maximum wavelengths from excitation ($\lambda_{max,ex}$) and fluorescence ($\lambda_{max,FL}$) spectra, lifetime of the short-living component (τ_1), long-living component (τ_2) and the average fluorescence lifetime (<τ>).

	$\lambda_{max,ex}$ (nm)	$\lambda_{max,FL}$ (nm)	τ_1 (ns)	τ_2 (ns)	<τ> (ns)
**In the absence of SNA**					
**ON8**	492	519	1.17	3.53	2.88
**ON5**	492	519	-	3.47	3.47
**ON6**	495	519	-	3.42	3.42
**ON7**	495	519	-	3.52	3.52
**In the absence of SNA**					
**ON8**	492	519	0.73	3.61	2.26 → 1.97
**ON5**	492	519	0.97	3.65	2.64 → 1.34
**ON6**	495	519	1.90 →* 1.37	3.85	3.09 → 2.72
**ON7**	495	519	2.67 → 1.52	4.30 → 3.95	3.52 → 3.12

* arrows show a change in the parameter caused by complex formation upon titration of SNA by increasing of ON concentrations from ON:SNA ratio 2:1 to 14:1.

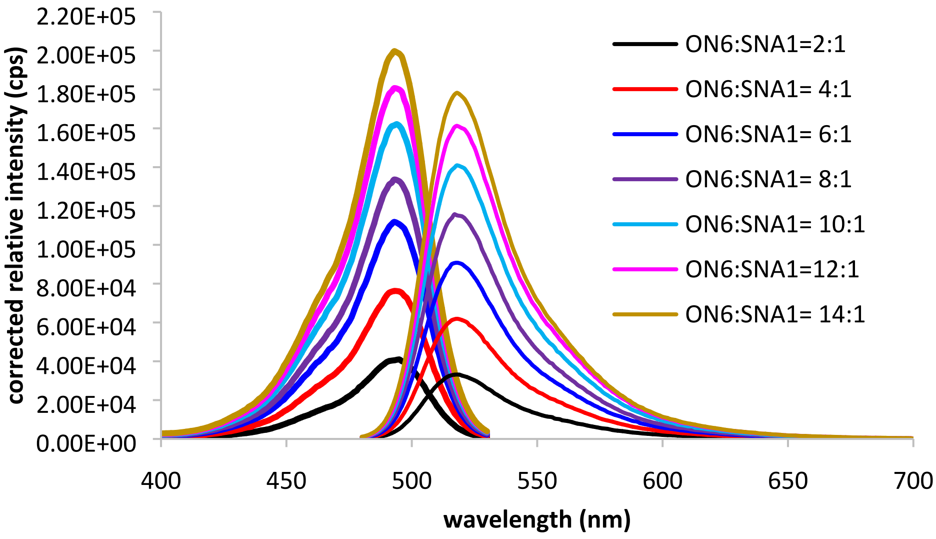

Figure S62. Fluorescence (solid lines) and excitation (dashed lines) spectra of **ON6/SNA1** complexes. The excitation wavelength was 470 nm and the excitation spectra were monitored at 540 nm.

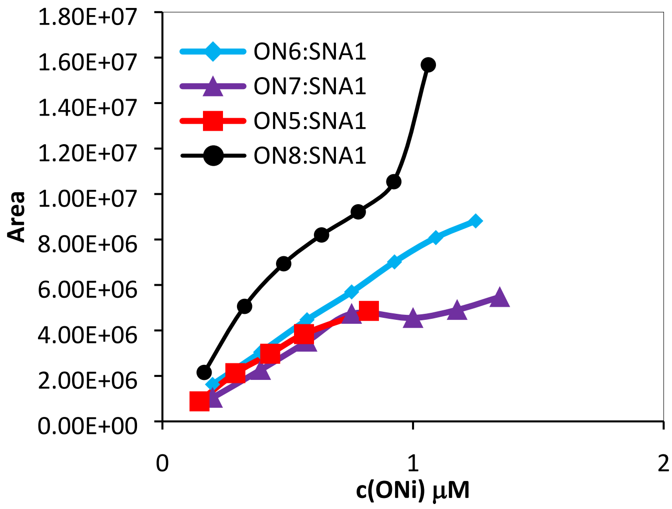

Figure S63. Area under the fluorescence spectra for **ON5-8/SNA1** complexes as a function of ON concentration.

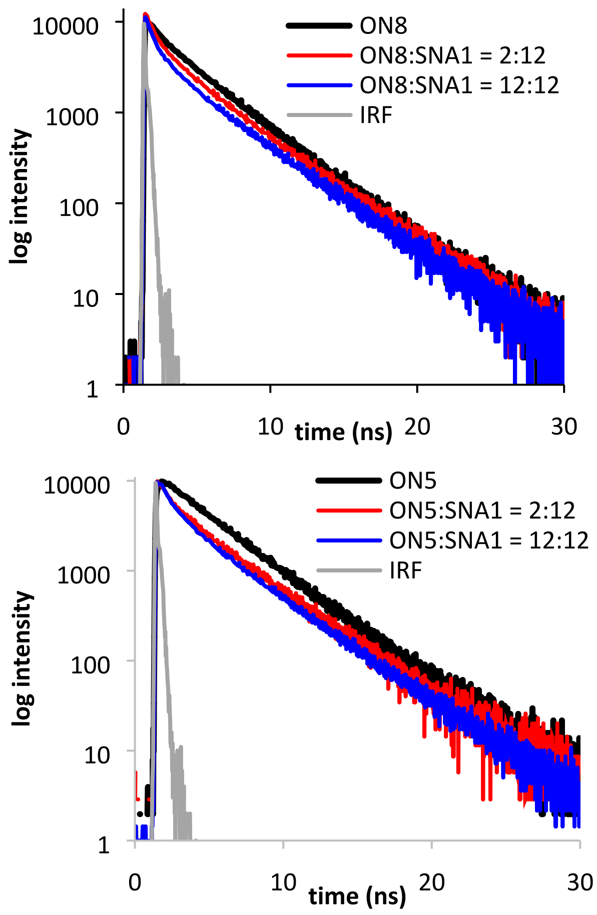

Figure S64. Fluorescence decays for **ON8** and **ON5** in the absence and presence of **SNA1**. The excitation wavelength was 483 nm and the decays were monitored at 520 nm. The instrumental response function in grey has a full width at half maximum of 130 ps.

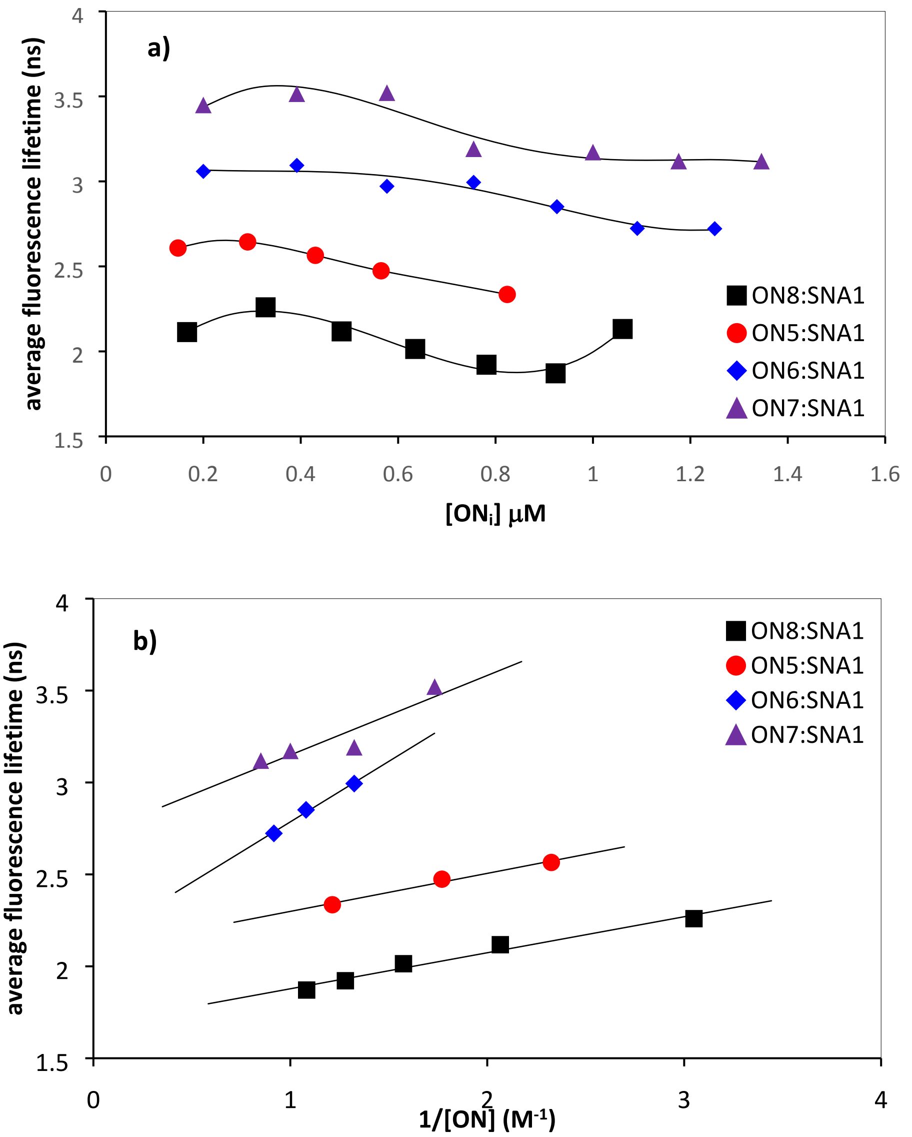

Figure S65. Plots of the average fluorescence lifetime <τ> of **ON5-8:SNA1** complexes as a function of a) ON concentration and b) inverse ON concentration. According to the independent binding model [1], the binding of a ligand (ONi) to a site on a macromolecule (SNA1) has no impact on simultaneous or subsequent binding to other unoccupied sites. The proportion of the ON_i bound by the **SNA1**, *B*, can be correlated to the association constant K by $B = \frac{K[ON_i]}{1+K[ON_i]}$. Taking the reciprocal of this equation, we obtain $\frac{1}{B} = 1 + \frac{1}{K[ON_i]}$. Thus, plotting $\frac{1}{B}$ as a function of $\frac{1}{[ON_i]}$ should yield a linear dependence with the association constant equal to the inverse of the slope. As the <τ> decreases with increasing ON_i concentration, it corresponds to $\frac{1}{B}$. Hence the association constants listed in Table 1 were determined from the slopes of the linear parts of the curves.

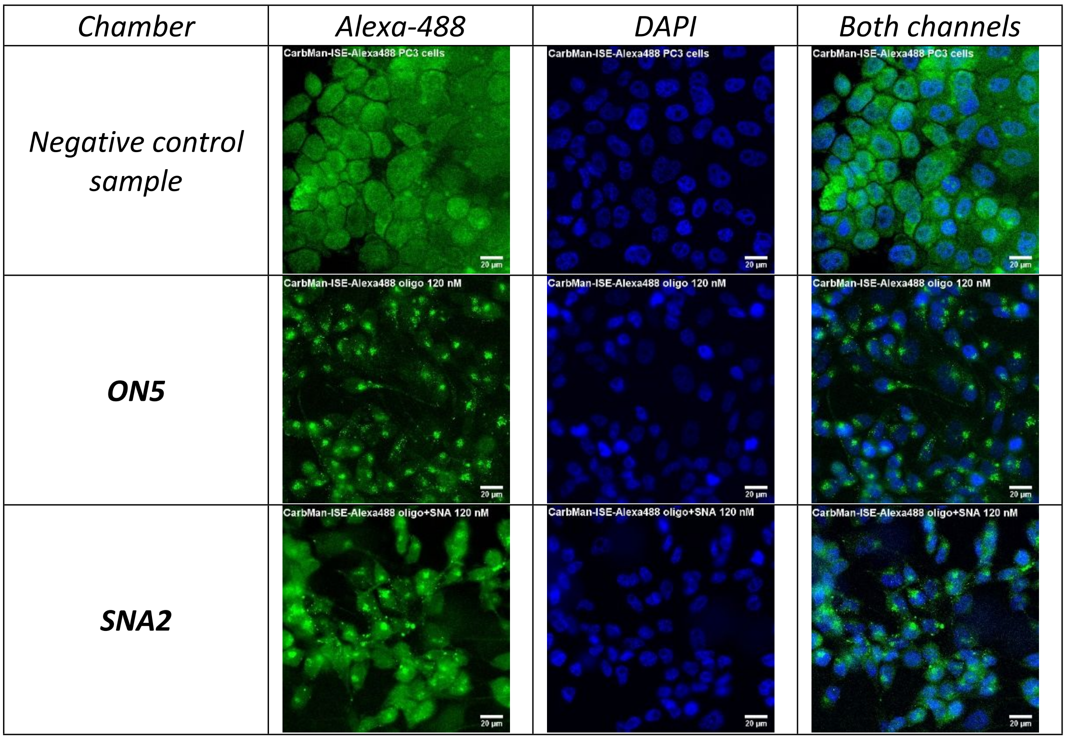

Figure S66. Uptake of AF488-labeled carbamoyl mannose conjugate **ON5** and **SNA2** by PC3 cells.

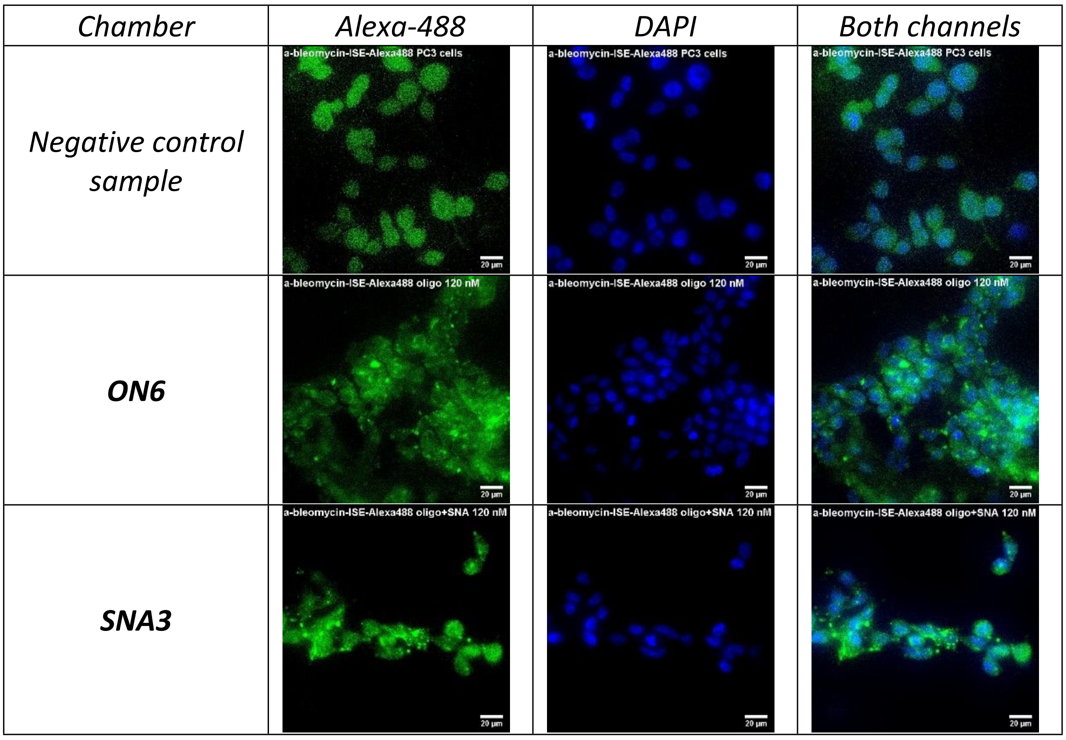

Figure S67. Uptake of AF488-labeled α-bleomycin disaccharide conjugate **ON6** and **SNA3** by PC3 cells.

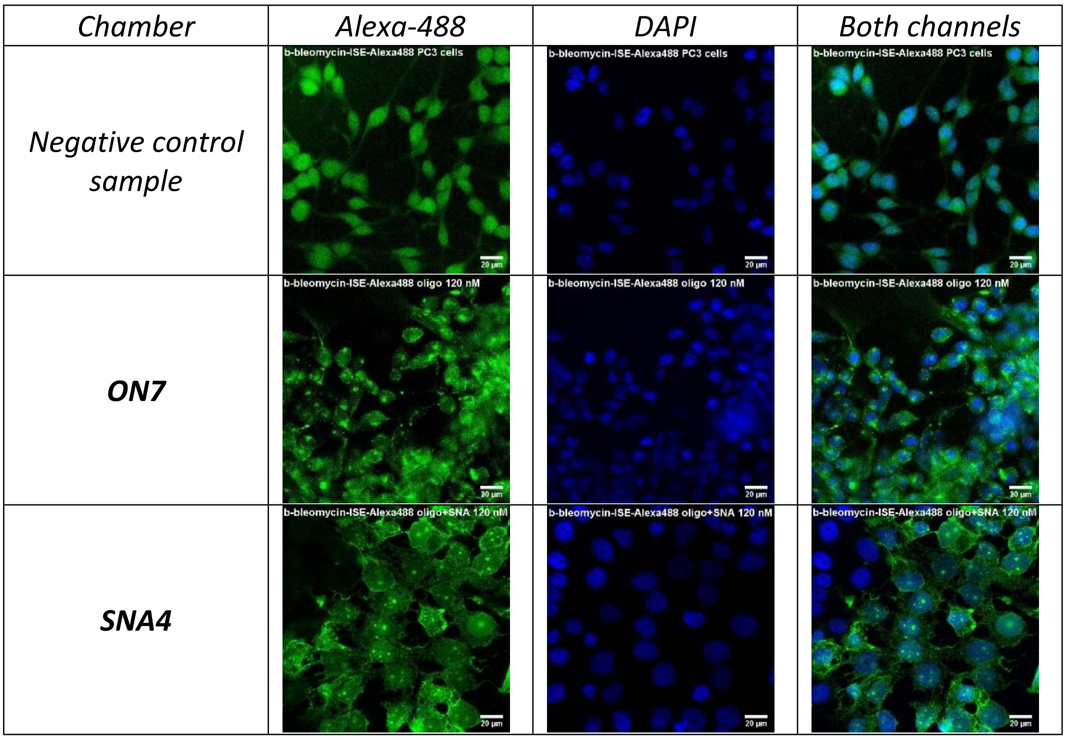

Figure S68. Uptake of AF488-labeled β-bleomycin disaccharide conjugate **ON7** and **SNA4** by PC3 cells.

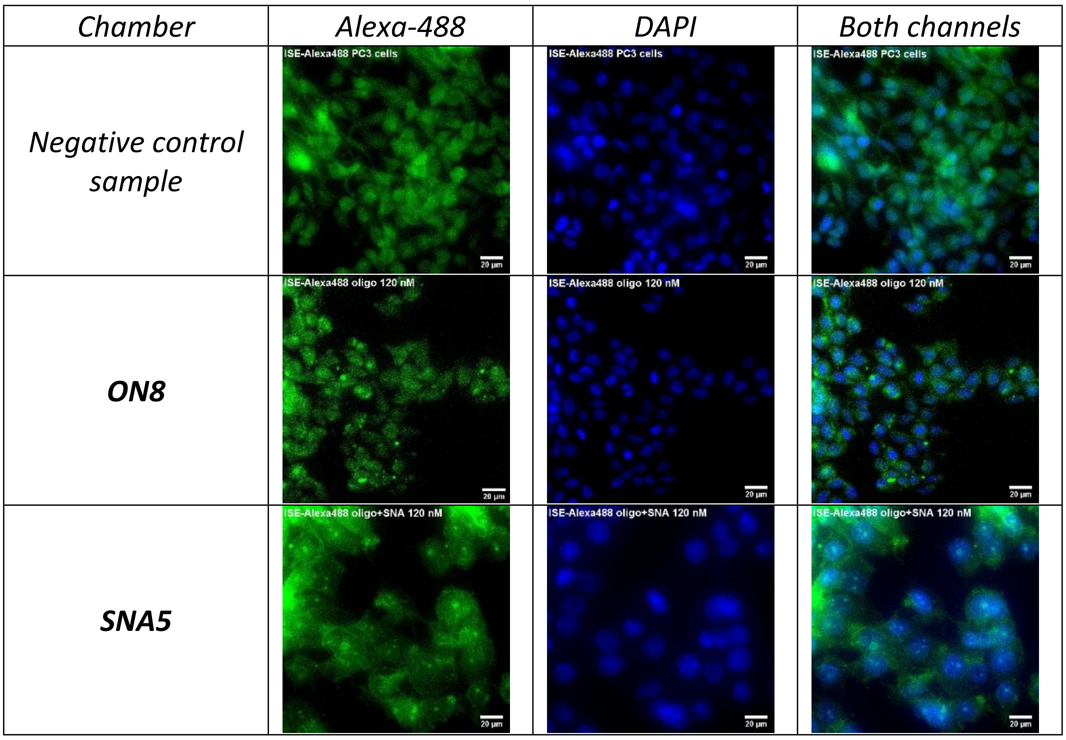

Figure S69. Uptake of non-conjugated reference oligonucleotide **ON8** and **SNA5** by PC3 cells.

References:

[1] Vuorimaa, E.; Urtti, A.; Seppänen, R.; Lemmetyinen, H.; Yliperttula, M. (2008) Time-resolved fluorescence spectroscopy reveals functional differences of cationic polymer-DNA complexes, *J. Am. Chem. Soc*. **2008**, *130*, 11695-11700.
